# Volatile Memristive Devices With Tunable Temporal Dynamics For Event‐Based Sensing

**DOI:** 10.1002/adma.74813

**Published:** 2026-08-30

**Authors:** Dimitrios Spithouris, Johannes Hellwig, Hugh Greatorex, Clemens Wittberg, Panagiotis Koutsogiannis, César Magén, Elisabetta Chicca, Regina Dittmann

**Affiliations:** ^1^ Peter Gruenberg Institute (PGI‐7) Forschungszentrum Juelich GmbH Juelich Germany; ^2^ Bio‐Inspired Circuits and Systems (BICS) Lab Zernike Institute for Advanced Materials University of Groningen Groningen Netherlands; ^3^ Groningen Cognitive Systems and Materials Center (CogniGron) University of Groningen Groningen Netherlands; ^4^ Instituto de Nanociencia y Materiales de Aragón (INMA) CSIC‐Universidad de Zaragoza Zaragoza Spain

**Keywords:** area‐dependent switching, event‐based sensing, ionic dynamics, neuromorphic computing, tunable temporal dynamics, volatile memristive devices

## Abstract

Volatile memristive devices with controllable temporal dynamics enable adaptation to diverse temporal coding tasks, making them attractive for low‐power neuromorphic edge applications that process asynchronous sensory streams. However, most reported volatile memristors rely on abrupt filamentary switching, suffering from high variability, unreliable operation, and the need for current compliance and forming steps. In this work, an area‐dependent volatile memristive device based on a Pt/α‐${\mathrm{SrTiO}}_{3}$/${\mathrm{TaO}}_{x}$/Ta stack is presented, exhibiting ionic‐based volatility and a CMOS BEOL‐compatible process flow. The device combines low variability with forming‐free, self‐compliant, rectifying operation and gradual, analog‐like switching, achieving an ON/OFF ratio of $∼10^{3}$. An in‐depth experimental and physical analysis of the mechanisms governing current transport and volatile behavior is provided. By exploiting the device's rich ionic dynamics, its volatility can be systematically tuned through applied stimulus and stack engineering, yielding decay time constants from tens of milliseconds to several seconds. A reliability study, including endurance and multilevel operation, demonstrates reproducible access to distinct volatile states. Finally, it is shown how a complete system, combining the device with spiking neuron models and CMOS circuitry, could exploit its programmable temporal dynamics for event‐based vision, with the wide range of decay constants supporting multiple sensory modalities.

## Graphical abstract

Tunable volatile memristive devices can serve various neural‐inspired tasks that require different time windows of information retention. The ionic‐based volatility of the presented Pt/a‐STO/TaOx/Ta device stack can be reproducibly and controllably tuned in multiple ways. The device's programmable temporal dynamics are exploited for event‐based vision applications, with the wide span of decay time constants enabling applicability across multiple sensory modalities.

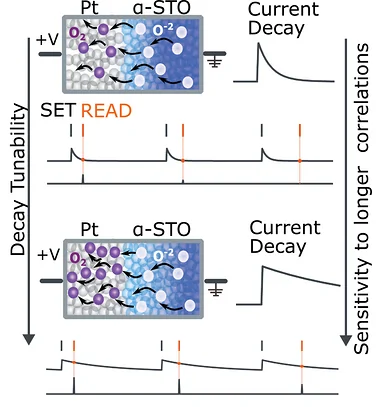

## 1 Introduction

Memristive devices have emerged as a highly promising candidate technology for the implementation of new computing paradigms like neuromorphic computing [1] and edge computing [2]. Memristors are categorized as either nonvolatile or volatile, depending on whether they retain their programmed state after the bias is removed. In the past years, the majority of efforts have focused on the development of nonvolatile devices to enable energy‐efficient in‐memory computing [3], as well as to exploit their excellent scalability, low fabrication costs, and the Back‐End Of Line (BEOL) compatibility with Complementary Metal Oxide Semiconductor (CMOS) technology [4]. Volatile memristive devices, on the other hand, show high potential for bio‐inspired neural network applications, as their intrinsic dynamics can be harnessed for sensing and learning. Various applications reported in the literature, like artificial neurons [5, 6], synaptic emulators [7, 8, 9, 10], artificial nociceptors [11, 12], artificial dendrites [13, 14], reservoir computing [12, 15, 16, 17, 18], eligibility traces [19] True Random Number Generators (TRNGs) [20, 21], and selectors for crossbar arrays [22, 23] make use of the dynamic nature of these elements.

While filamentary switching volatile devices have attracted interest for their fast switching and high scalability, they are affected by issues like inherent high variability and noise level, unreliable filament formation, need for current compliance, CMOS‐incompatible forming process, and very low ON‐state that yields high energy consumption [24, 25, 26, 27]. There have been studies showing that volatile filamentary devices can showcase a tunable current relaxation behavior [28, 29], but such behavior is obtained without resolving the underlying device‐level challenges. Although the inherent variability of these devices can be utilized for TRNGs and stochastic computing applications [30, 31], reliable and reproducible operation is a big necessity in the majority of cases.

Unlike their filamentary counterparts, area‐dependent volatile devices exhibit intrinsically low variability and gradual switching with rich temporal dynamics, enabling tunable current relaxation behavior for analog computing applications [32]. In addition, these devices exhibit forming‐free, current compliance‐free, and self‐rectifying properties that minimize the peripheral circuit needed, making the fabrication of passive crossbar arrays possible [6, 33]. Although most studies on this type of device report gradual switching behavior with low variability, systematic investigations on the variability and reproducibility of current decay, as well as the accessibility and controllability of distinct decay states, remain limited [6, 8, 9, 11, 12, 15, 16, 17, 18, 34, 35]. This lack of systematic analysis is especially evident in area‐dependent volatile devices with ionic‐based volatility. Many studies focus on the conductance state modulation during the SET process, while the full current decay transient—more sensitive to ionic dynamics and kinetics—is rarely analyzed. Furthermore, a number of reported devices display incomplete relaxation to the baseline, considerably high switching voltages and non‐CMOS‐compatible for BEOL‐integration processes, aspects that need to be considered for practical applications [7, 8, 9, 11, 36].

Moreover, existing studies often lack clear experimental evidence and a comprehensive physical framework for the observed decay behavior [6, 8, 12, 16, 17, 18, 35], and physical models capable of describing the current decay dynamics remain scarce [6, 8, 9, 11, 12, 15, 17, 35]. In addition to this, comprehensive transport studies that elucidate the underlying conduction mechanisms in the volatile regime are still scarce. Many reports rely on simple fits of the *I*–*V* characteristics to standard models, often without assessing the physical realism of the extracted values or performing targeted experiments to disentangle the contributions of other processes, like ionic motion [9, 10, 13, 15, 36]. A detailed understanding of the physical mechanisms, both in terms of decay dynamics and charge transport, is crucial, as it provides a rational basis for engineering area‐dependent volatile devices and optimizing their properties to meet the requirements of specific applications.

Volatile memristive devices with tunable decay dynamics could serve various neural‐inspired tasks that require different time windows of information retention while maintaining low circuit complexity [37]. The proliferation of distributed sensors at the network edge generates vast streams of data that must be processed locally and under stringent power and often latency constraints, rendering conventional *von Neumann* architectures increasingly impractical [38]. Event‐based sensing [39, 40] offers an elegant solution to this challenge: rather than sampling the environment at fixed intervals, event‐driven sensors respond only when meaningful changes occur, significantly reducing data redundancy and power consumption. This paradigm aligns naturally with biological sensory systems, where neurons encode information through the precise timing and temporal correlation of spikes. Extracting task‐relevant features from such asynchronous event streams requires processing elements with inherent temporal dynamics, devices whose response depends on the history and timing of input stimuli. In this context, volatile memristive devices with tunable time constants are particularly attractive, as they can natively perform temporal correlation and short‐term memory functions essential for efficient event‐driven processing at the edge. By adjusting the decay dynamics of a single device, the same hardware element can be adapted to different temporal windows, enabling flexible deployment across diverse sensing and learning scenarios without additional circuit overhead.

In this work, we demonstrate a volatile, area‐dependent memristive device of the Valence Change Memory (VCM) type [41] based on amorphous ${\mathrm{SrTiO}}_{3}$ (α‐STO) using a Pt/α‐STO/${\mathrm{TaO}}_{x}$/Ta stack compatible with BEOL CMOS integration. The devices combine low variability with gradual switching, a high on/off ratio ($∼10^{3}$), endurance beyond $1.2\times 10^{6}$ cycles, programmable multi‐state volatility, and forming‐free, self‐compliant, and self‐rectifying operation. Through deliberate stack engineering and temperature‐dependent measurements, we identify the transport mechanism and determine the role of each layer of the stack. Thereafter, the ionic nature of the decay is discussed and a physical model is used to describe it. We explore ways to tune the current decay via the SET stimulus, read‐out conditions, and variations in layer thickness or electrode materials, enabling precise control of temporal dynamics. Moreover, we conduct a systematic study on the reproducibility of the decay and the stability of different decay states. Finally, we demonstrate system‐level relevance by employing the device in an event‐based neuromorphic sensing task, where its programmable time constants enable spatio‐temporal correlation of asynchronous signals and tunable temporal sensitivity in conjunction with CMOS neuron circuits.

## 2 Results and Discussion

### 2.1 Device Characteristics of the α‐STO‐Based Volatile Memristor

The area‐dependent volatile α‐STO‐based device stack schematic is shown in Figure 1a. The devices are fabricated on a ${\mathrm{SiO}}_{2}$/Si substrate using CMOS BEOL‐compatible process temperatures $\leq 400^{\circ }C$. First, 40 nm of Ta is sputtered as the bottom electrode. Then, 5nm of α‐STO is grown by Pulsed Laser Deposition (PLD), followed by electron beam evaporation of 30 nm Pt top electrode. Further fabrication details are provided in the Methods. Due to the elevated temperature during the α‐STO deposition, a native ${\mathrm{TaO}}_{x}$ layer of approximately 3.5 nm is formed. The ${\mathrm{TaO}}_{x}$ thickness has been found to remain stable in different samples with the same α‐STO thickness by X‐Ray Reflectivity (XRR) measurements on same stacks fabricated in a time period of 1.5 years (see Supporting Information S1), highlighting the reproducibility of the device fabrication. As can be seen from the High‐Angle Annular Dark‐Field (HAADF) image and the Energy‐Dispersive X‐ray Spectroscopy (EDX) elemental maps obtained by Scanning Transmission Electron Microscopy (STEM) in Figure 1b and Figure 1c, respectively, the ${\mathrm{TaO}}_{x}$ layer is uniform and well defined, with a thickness slightly smaller than that of the α‐STO layer, in agreement with the XRR predictions. The corresponding EDX line profile, presented in Supporting Information S2, shows that the interfaces are gradual, primarily due to interface roughness. Although some interdiffusion is observed, it remains minimal. In addition, no crystalline structure is observed in the STO layer. The amorphous structure of the STO is confirmed by the absence of peaks in the Grazing Incidence X‐ray Diffraction (GIXRD) measurement, see Supporting Information S3.

**FIGURE 1:**
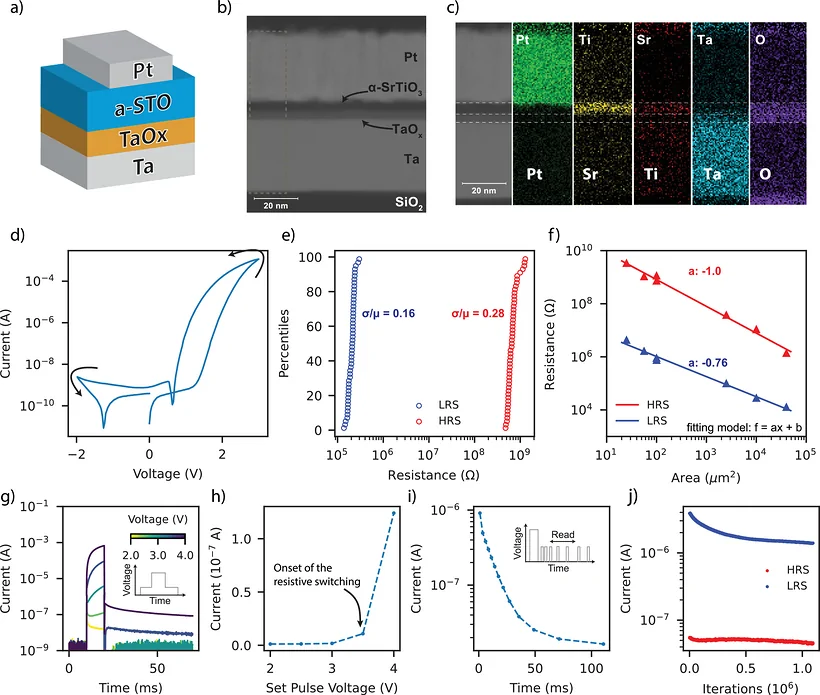
The volatile α‐STO‐based device stack and its electrical characteristics. (a) The Pt/α‐STO/${\mathrm{TaO}}_{x}$/Ta device stack. (b) Cross‐sectional HAADF‐STEM image from the device stack. (c) EDX elemental maps collected from the scanned region marked in (b). (d) Characteristic *I*–*V* sweep curve from a $50\times 50\;\mu m^{2}\;$ device pad size. The voltage sweep (0 to 3 V to −2 to 0 V) is applied on the Pt top electrode. (e) Cumulative Distribution Function (CDF) plot of Low Resistive State (LRS) and High Resistive State (HRS) from 50 different 50 × 50 $\mu m^{2}$ devices, see Supporting Information S4, with resulting variation coefficients of 0.28 and 0.16 for the LRS and HRS, respectively. The current values are obtained at 1.4 V. (f) Area‐dependency of the LRS and HRS from normal pad devices with sizes of 10×10, 50×50, 100×100, and $200\times 200\;\mu m^{2}\;$ and single crossbar devices of 5×5, 7.5×7.5, and $10\times 10\;\mu m^{2}\;$ device area. The resistance is linearly scaling with the pad size and the slopes of the linear fittings are shown in the plot. Optical microscope images of the fabricated samples as well as the characteristic *I*–*V* plots from the different device areas are provided in Supporting Information S5. (g) Current evolution during a voltage sequence consisting of a 10 ms READ period of 1.2 V, a 10 ms voltage pulse ranging from 2 to 4 V followed by a 50 ms READ period of 1.2 V. (h) Current as a function of SET pulse voltage, extracted from the post‐SET READ period at t=40ms of the sequence presented in (g). A switching activation threshold behavior is observed between 3.5 V and 4 V. (i) Current decay response after a 4.2 V and 20 ms SET pulse with a logarithmically spaced pulsed‐read‐out voltage of 1.2 V. The measurement pulse scheme is shown in the inset. (j) Endurance measurement showing $>1.2\times 10^{6}$ consecutive SET and RESET cycles. SET pulses of 4 V and 10 ms and RESET pulses of −0.9 V and 6 ms were applied. Read‐out takes place directly after each SET and RESET pulse at 1.1 V for both cases.

Strontium titanate (STO), a multifunctional model material oxide with well‐understood defect chemistry [42, 43, 44, 45], has been widely studied for its tunable physical properties and demonstrated as a high‐*k* dielectric [46] and in various other applications [47, 48, 49, 50, 51]. In addition, numerous studies have reported the use of both crystalline and amorphous STO films in memristive devices exhibiting volatile and nonvolatile behavior, as summarized in Table S1. However, similarly to many oxides, its crystalline form typically requires epitaxial growth at temperatures exceeding 400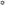 C, which is incompatible for CMOS–BEOL integration. In this regard, amorphous oxide counterparts are more suitable for integration in practical device architectures.

In Figure 1d, the *I*–*V* characteristic of the α‐STO‐based device is shown. The device exhibits an eightwise switching, which stems from the migration of oxygen vacancies toward and inside the Pt top electrode and the modulation of the Schottky barrier in the Pt/α‐STO interface [52, 53]. Further details of the underlying switching mechanism are provided in the next Section 2.2. Gradual switching behavior, and a high ON/OFF ratio exceeding $10^{3}$ is attained, enabling the realization of multiple distinct programmable states. Considering the high rectifying ratio of $>10^{4}$ and no requirements for a forming step and compliance current, the devices demonstrate a significant potential for implementation of passive crossbar arrays with minimal peripheral circuitry [6, 33]. The inherent low variability of area‐dependent devices is confirmed from the low variation coefficients of the CDF plots in Figure 1e. In Figure 1f, the linear dependence of the current levels on electrode area, in combination with the forming‐free operation, validates the area‐dependent nature of the switching.

To probe the SET dynamics of our device, we applied a voltage step consisting of a pulse with an amplitude varied between 2 and 4 V and a fixed width of 10 ms; the device state was read before and after the pulse employing a continuous voltage of 1.2 V. The corresponding current response is shown in Figure 1g. During the SET pulse, the current gradually increases for all voltages exceeding 2 V, indicating a change in resistive state. After the pulse, the current returns to its initial level for amplitudes below 3.5 V, but exhibits an abrupt jump between 3.5 and 4V. Beyond this threshold, the post‐pulse current continues to rise, as seen for the 4 V pulse. This behavior is demonstrated in Figure 1h showing the extracted current during the post‐SET READ period at t = 40 ms plotted against the SET pulse voltage. Here, a strong increase in current is observed between 3.5 and 4V. Once the threshold is reached, a volatile resistive state is observed, which decays back to its initial pre‐SET baseline within milliseconds.

A complete current decay after an applied SET pulse of 4.2 V and 20 ms, measured using a logarithmically spaced pulsed READ protocol (inset), is shown in Figure 1i. The current decay is characterized by between one and three distinct time constants, which are indicative of the complex ion‐transport dynamics occurring at the Pt/α‐STO interface, as elaborated in Section 2.2.

Moreover, the devices demonstrate an endurance beyond $1.2\times 10^{6}$ cycles with minimal degradation, as shown in Figure 1j. Here, the LRS corresponds to the current measured immediately after the voltage SET pulse, while the HRS denotes the equilibrium current value. While the devices are volatile, RESET pulses are used to accelerate the measurement, as waiting for the device to completely relax back to the HRS would significantly prolong the measurement time. A shorter endurance measurement consisting of 2000 cycles of a SET pulse followed by complete device relaxation is presented in Supporting Information S7 for comparison. Owing to the simpler pulsing scheme and the complete relaxation of the device between cycles, highly stable operation with an increased ON/OFF ratio is observed. The excellent endurance is further reflected in the decay variability measurements, shown in Section 2.3, an essential characteristic that paves the way for event‐based computing applications.

Our devices show highly desirable characteristics necessary to function as dynamic elements in neuromorphic computing applications. However, to effectively modulate their properties and employ them in diverse applications, a deep understanding of the physical mechanisms underlying their operation is crucial.

### 2.2 Physical Mechanisms

#### 2.2.1 Switching and Transport Mechanism

It is spectroscopically verified in VCM crystalline‐STO memristive devices that switching in eightwise polarity (e.g. SET at positive voltage and RESET at negative voltage) arises from an oxygen exchange with the Pt electrode and the resulting modulation of the Pt/STO Schottky barrier [52, 53, 54, 55]. As depicted in Figure 2a,b, during SET oxygen ions migrate from the STO into the Pt electrode, where the undergoing oxygen exchange reaction leads to the release of molecular oxygen and generation oxygen vacancies near the Pt/STO interface [41]. The increased oxygen vacancy concentration narrows the Schottky barrier resulting in enhanced tunneling current. Conversely, during RESET oxygen vacancies are annihilated, reducing the space charge and broadening the depletion region [56].

**FIGURE 2:**
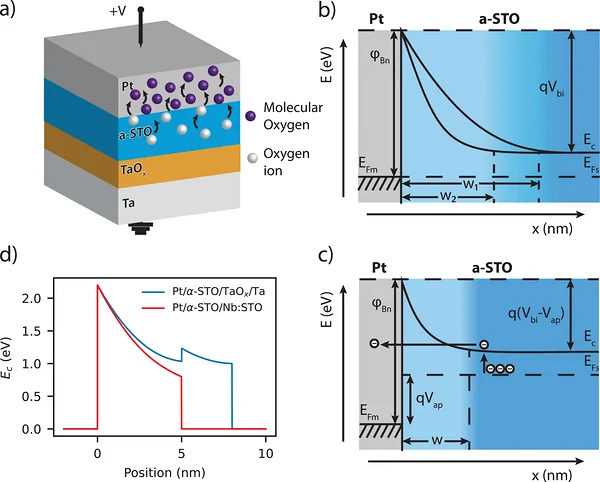
Transport mechanism. (a) Device stack schematic illustrating oxygen migration at the current‐dominating Pt/α‐STO interface under a positive voltage bias on the Pt electrode. The oxygen ions toward the Pt electrode, undergoing a redox‐reaction at the interface and leaving behind a reduced α‐STO. (b) Exemplary energy band diagram of the current‐dominating Schottky barrier for the LRS and HRS at zero bias. The depletion region width $w_{1}$ of the HRS is bigger than the depletion region width $w_{2}$ of the LRS. Here, $\phi_{Bn}$ is the Schottky barrier height, $E_{Fm}$ and $E_{Fs}$ the metal and semiconductor Fermi levels, respectively, $V_{bi}$ the built‐in potential, $V_{ap}$ the applied bias and $E_{c}$ the conduction band minimum. (c) Comparison between the simulated energy band diagrams of the Pt/(5nm)α‐STO/($3\mathrm{nm}){\text{TaO}}_{x}$/Ta and Pt/(5nm)α‐STO/Nb:STO devices calculated by solving the Poisson equation in equilibrium using parameters listed in Table S2, with adjustments to match the fitting parameters reported in Table S3. (d) Exemplary energy band diagram of the Schottky barrier under forward bias, showing the Tsu–Esaki tunneling current from α‐STO into the Pt electrode.

Given the comparable switching polarity, volatility (but with shorter decay times), gradual switching dynamics (Figure 1g and Supporting Information S8) and SET characteristics (threshold activation in Figure 1h,i) of the presented α‐STO devices, it is reasonable to infer that analogous ionic mechanisms govern the resistive switching and state relaxation, as in the case of c‐STO devices. However, what sets the α‐STO devices apart from their crystalline counterparts is the presence of the ${\mathrm{TaO}}_{x}$ layer, and it is therefore critical to clarify its contribution to the conduction and, consequently, to the switching behavior.

By careful stack engineering experiments and by analyzing the electrical transport, we can prove that the current limiting barrier and the one that defines the switching is the Schottky barrier at the Pt/α‐STO interface. First, we investigated the bulk conduction through the α‐STO, by fabricating a sample with a Pt/Ta/α‐STO/${\mathrm{TaO}}_{x}$ stack in order to suppress the formation of the Pt/α‐STO Schottky barrier, see Supporting Information S9 and Methods. This is possible because the work function of STO and Ta are comparable, see Table S2. This device exhibited highly leaky conduction with no switching, indicating that the bulk conduction of α‐STO is not current limiting in the small to medium bias regime. Therefore, we can exclude its contribution to the switching.

To probe the conduction exclusively through the ${\mathrm{TaO}}_{x}$ layer, we fabricated a Pt/Ta/${\mathrm{Ta}}_{2}O_{5}$/Ta stack incorporating a 3.5 nm fully oxidized Ta layer, see Methods. The band diagram of the Ta/${\mathrm{Ta}}_{2}O_{5}$/Ta barrier is assumed to closely resemble that of the α‐STO/${\mathrm{TaO}}_{x}$/Ta, owing to the comparable work function of STO and Ta, see Table S2. The measured *I*–*V* characteristics are accurately described by the trap‐assisted tunneling (TAT) model, proposed in Supporting Information S10. The model accurately fits the data and yields physically reasonable parameters, including an effective oxide thickness of ∼3.6 nm and a oxygen vacancy‐related trap density of $N_{t}\approx 4\times 10^{16}cm^{-3}$. Despite the comparatively low currents of this stoichiometric ${\mathrm{Ta}}_{2}O_{5}$ barrier, the naturally formed ${\mathrm{TaO}}_{x}$ layer is expected to exhibit a higher oxygen vacancy concentration, acting as trap states [57]. Simulations using this higher concentration predict current densities several orders of magnitude higher than those measured experimentally, even at low bias. These results indicate that ${\mathrm{TaO}}_{x}$ with realistic stoichiometry is intrinsically highly conductive and therefore cannot be the current‐limiting element of the device stack. To correctly describe the measured I–V characteristics of the full stack, it is therefore necessary to focus on the Pt/α‐STO Schottky barrier. This naturally raises the question: what role does the ${\mathrm{TaO}}_{x}$ layer play?

The ${\text{TaO}}_{x}$ layer, although not being the current‐limiting barrier, still plays an essential role: it acts as a protective layer and limits the change in the potential barrier across the Pt/α‐STO Schottky junction, enabling the devices' self‐compliance behavior. To verify the role of the ${\mathrm{TaO}}_{x}$ layer, we examine the device behavior in its absence. To this end, we fabricated a Pt/α‐STO/Nb:STO stack to avoid the formation of a protective oxide between the bottom electrode and the α‐STO layer, see Methods. As a result, the devices incorporating a Nb:STO bottom electrode do not exhibit the same self‐compliance behavior. Instead, they undergo an initial electroforming step, followed by subsequent filamentary resistive switching, as demonstrated in Supporting Information S11.

This difference can be rationalized by comparing the corresponding energy band diagrams. Figure 2d presents the calculated band diagrams of the two device stacks. In this configuration, the ${\mathrm{TaO}}_{x}$ layer modulates the band bending within the α‐STO, leading to a reduction of the local electric field. This limitation of the electric field indirectly dictates the voltage dependence of the total current traversing the stack, while simultaneously enabling controlled oxygen‐ion migration at the expense of the switching rate.

Due to the highly leaky ${\text{TaO}}_{x}$ layer, the current is fully dominated by the Schottky barrier. To further investigate the electrical transport mechanism, we performed temperature‐dependent pulsed *I*–*V* measurements. In this approach, a series of voltage pulses of varying amplitude, long enough to probe the electronic conduction, yet short enough to avoid resistive switching, are applied to the device, and the resulting conductivity is measured [58]. In this way, we can disentangle electron transport from ion migration‐related effects.

The corresponding experiments are presented in Supporting Information S12. We observed a strongly temperature‐dependent, nonlinear *I*–*V* response, which is a characteristic of Schottky barrier tunneling typically observed in VCM‐type memristive devices. Indeed, the observed behavior is well described by the Tsu–Esaki tunneling model, see Supporting Information S13, which we confirmed by quantitative fits to our experimental data shown in Supporting Information S12 [56].

Figure 2c shows this tunneling mechanism through the Schottky barrier under forward bias. The applied voltage leads to narrowing of the depletion region. Electrons are thermally excited from the Fermi level and are injected into the Pt electrode via a tunneling process, which depends exponentially on the depletion region width and accounts for the strong nonlinearity observed in the *I*–*V* characteristics.

Understanding this tunneling mechanism is essential for interpreting the device operation and the relaxation measurements, since during the decay the Schottky barrier is actively probed by applying READ pulses. The READ amplitude must be high enough, typically between 0.9 and 1.2V, to achieve a sufficient tunneling current to probe the depletion zone width. At these voltages the oxygen ion migration can be influenced and the decay time becomes a function of the READ pulse sequence. As we will show in Section 2.3, the READ sequence can be engineered to modulate the relaxation time constant which may be exploited in various neuromorphic applications.

#### 2.2.2 Current Decay Dynamics and Microscopic Insight

The physical origin of the current relaxation is reflected in its decay dynamics, in particular in the distribution of characteristic time constants. In many memristive systems, the decay cannot be described by a single exponential process but instead exhibits multiple characteristic time scales [59, 60, 61]. In c‐STO‐based devices, this behavior has been attributed to the complex ionic dynamics at the Pt/STO interface [62].

Based on the analysis in Section 2.2 and the large activation energy of the current decay, we exclude electronic contributions as the origin of these dynamics [63]. The absence of current decay below the threshold of resistive switching indicates that a sufficiently large activation energy is required to activate the relaxation processes governing the decay. This stands in contrast to electronic processes, which rely on the charging and discharging of trap states within the depletion region and should occur largely independently of the applied voltage.

In addition, the emission of electrons after the SET pulse would yield negative currents, that is, the opposite polarity to the ionic decays presented in Figure 3 [63]. To further support the ionic origin of the decay, cyclic voltammetry measurements were conducted (see Supporting Information S14). The switching window decreases with increasing stimulus frequency, consistent with the limited ability of ions to follow rapidly varying voltage signals.

**FIGURE 3:**
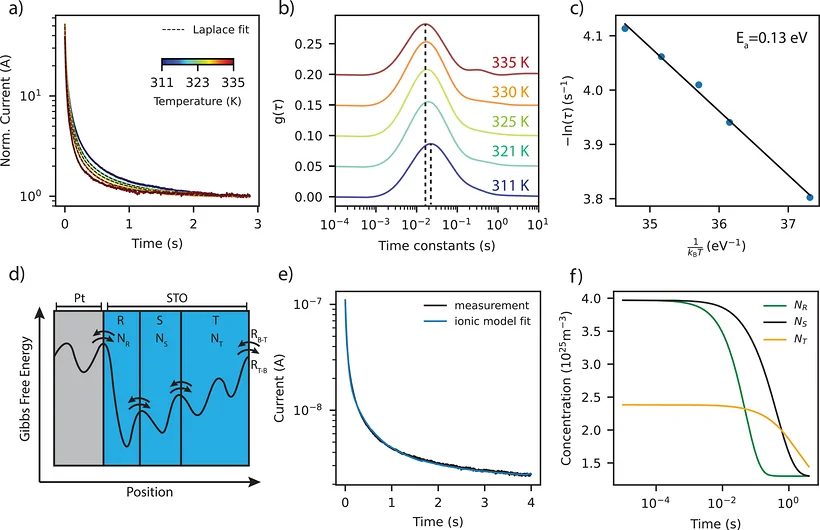
Physical current decay model. (a) Temperature‐dependent current decay measurements between 300 and 335 K. The SET pulse is fixed at 3.7 V and 10 ms for all temperatures. (b) Time‐constant spectrum extracted from the current decay measured after a 3.7 V, 10 ms voltage pulse using an inverse Laplace transformation (see Supporting Information S15). The dashed lines show the drift to longer time constants at lower temperatures. (c) Arrhenius plot of the peak positions of the spectra, showing an exponential relationship with inverse temperature. The fitting details are described in Supporting Information S16. (d) Schematic illustration of the Gibbs free‐energy landscape of the device based on the relaxation model proposed by Hellwig et al. [62]. The STO is divided into three coupled regions, recombination (R), supply (S), and transition (T), between which ionic exchange can occur. (e) Measured current decay together with the fit based on the physical decay model. (f) Evolution of the oxygen‐vacancy concentration in the three slabs shown in (d).

To investigate the decay dynamics of our α‐STO devices, we extracted the time‐constant spectrum of the current decay by approximating the transient as the Laplace transform of a spectral density function g(τ) using an inverse Laplace transformation [64]. The current decay was measured at temperatures between 300 and 335K (Figure 3a), and the corresponding time‐constant spectra g(τ) were extracted (dashed black lines). Each decay is characterized by a single broad peak (Figure 3b), which shifts with increasing temperature. From the Arrhenius plots in Figure 3c, a surprisingly small activation energy of 0.13 eV is obtained, which appears inconsistent with the ionic picture introduced above.

To resolve this apparent discrepancy between measured and intrinsic activation energies, we employ the ionic model proposed by Hellwig et al. [62]. In this model, the current decay is described as an ionic hopping process within the complex Gibbs free‐energy landscape at the Pt/STO interface.

During the SET process, oxygen ions are incorporated into the Pt electrode, and during relaxation they recombine with oxygen vacancies at the interface. The Gibbs free‐energy landscape at the Pt/STO interface is illustrated in Figure 3d. This incorporation process is accelerated by the steep electrochemical potential gradient and the high density of oxygen vacancies in this region. Meanwhile, vacancies must be replenished from the oxide bulk to sustain ongoing reincorporation of oxygen, introducing a slower dynamical component. This refill process transiently alters the interfacial vacancy concentration, thereby modulating the tunneling current through the Schottky contact. Together, these coupled processes naturally give rise to multiple decay time constants.

In the decay model, ion migration within the depletion region is represented by three coupled slabs (R, S, T), as shown in Figure 3d. The Pt electrode and the STO bulk act as oxygen reservoirs supplying oxygen ions to the depletion region. R and S are of fixed width on the order of the lattice parameter of c‐STO (0.39 nm), whereas T covers the remaining bulk‐facing part of the depletion region. Oxygen vacancies are exchanged between adjacent slabs via thermally activated hopping processes characterized by distinct activation energies and transition rates (for details see Supporting Information S17 and Ref. [62]). The time evolution of the vacancy concentration in each slab, $N_{R}$, $N_{S}$, and $N_{T}$, governs the depletion width and thus the transient tunneling current.

Following the analysis of Section 2.2, the current transport is described by the current‐limiting Schottky barrier. Due to the high leakiness of the ${\mathrm{TaO}}_{x}$ layer and the STO bulk, this approximation is valid and yields transport parameters of the same order of magnitude as the more complex current‐transport model that includes the modulation of the STO band bending by the ${\mathrm{TaO}}_{x}$ layer (Supporting Information S12). In addition, due to the leaky nature of ${\mathrm{TaO}}_{x}$ and the resulting small voltage drop across it, oxygen ion migration and vacancy redistribution within this layer are expected to be negligible compared to those occurring in the STO layer.

A detailed description of the model and the parameter extraction procedure is provided in Ref. [62] and Supporting Information S17. The initial vacancy concentrations, migration barriers, Schottky barrier height, and dielectric constant $\varepsilon_{r}$ were matched to the experimental data.

Using this model, we reproduce the decay dynamics at 300K shown in Figure 3a, as indicated by the dashed line in Figure 3e. The corresponding evolution of the oxygen‐vacancy concentration in the individual slabs is shown in Figure 3f. The migration barrier responsible for the initial fast decay is estimated to be 0.68eV. The subsequent barriers are 0.76, 0.78, and 0.9 eV for deeper regions of the oxide. Only the initial vacancy concentrations and migration barriers were treated as adjustable parameters, while geometric and electronic transport parameters were fixed.

Due to the lower activation barrier, the recombination slab fills most rapidly, governing the fast decay observed at the beginning of the transient. All slabs are coupled, and oxygen ions migrate progressively from the interface toward the bulk until all regions reach the HRS vacancy concentration of 1 × 10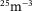.

The slab closest to the interface, $N_{R}$, represents the interface reaction, while $N_{S}$ and $N_{T}$ exhibit larger time constants reflecting bulk activation processes. The first two slabs were initialized with an oxygen‐vacancy concentration of 4 × 10^25^ $m^{-3}$, while the third was initialized with 2.4 × 10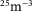. Matching the electronic transport parameters to the experimental results suggests a Schottky barrier height of 2.0 eV, which is close to the values obtained from the more comprehensive physical model. A list of all parameters used in the simulations is provided in Supporting Information S17.

The model reveals the origin of the small activation energy extracted from the Arrhenius fit. This discrepancy arises from the nonlinear coupling between electronic and ionic transport that is characteristic of VCM‐type memristive devices, specifically tunneling conduction through the Schottky barrier [65]. In such systems, even small changes in the electronic configuration of the depletion region are exponentially amplified in the tunneling current. Consequently, the experimentally measured time constants do not directly reflect the intrinsic ionic processes but instead appear reduced.

The individual time constants originate from the different migration barriers and their transient evolution, while the effective decay time constant results from a superposition of these contributions. In Section 2.3, we show that the decay dynamics are tunable, a property particularly relevant for event‐based sensing applications, where different time constants enable distinct sensing modalities.

### 2.3 Tunability of Current Relaxation

The current decay can be deliberately modulated either by tuning ion migration through biasing conditions or by engineering the device stack. In the first case, different biasing protocols enable access to distinct decay times, while in the second, modifications to the stack determine the accessible range of decay times. This dual approach enables a wide range of decay times to be realized within a single device. Figure 4 summarizes different strategies to tailor the decay. To track these and given the complex time constant spectrum, we introduce the effective time constant $\tau_{\mathrm{eff}}$ as the time required for the current to drop to 1 % of the total decay.

**FIGURE 4:**
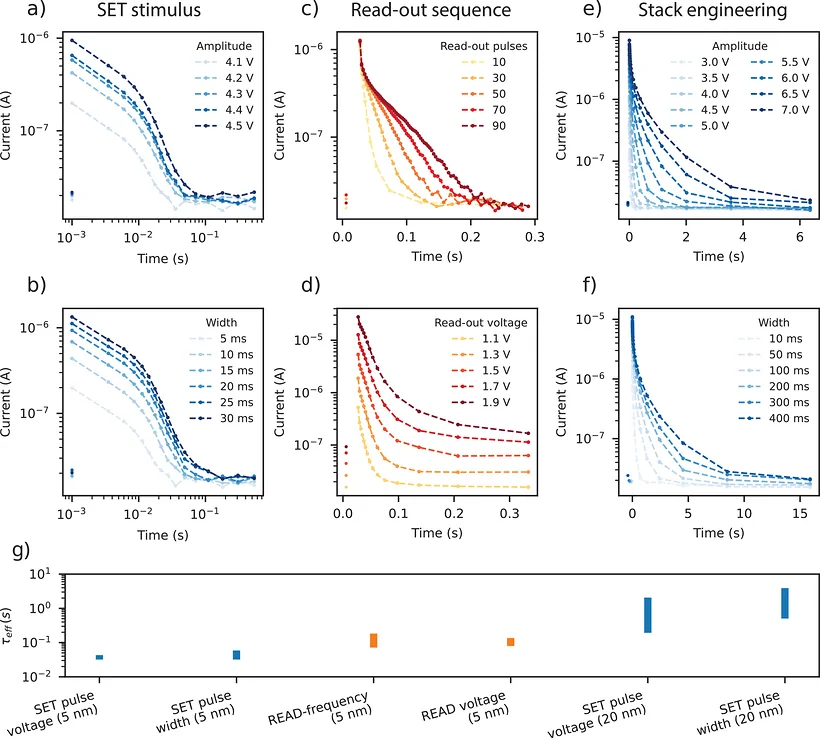
Tunability of the current decay. Stimulus‐dependent volatility for (a) varying amplitude ranging from 4.1 to 4.5 V with a fixed width of 5 ms, and (b) varying pulse width between 5 and 30 ms with a fixed amplitude of 4.2 V. The read‐out voltage is set to 1.2 V. The *x*‐axis is logarithmic to make the differences in decay times clear. TheHRS of the device before each measurement is monitored by a read‐out pulse prior to the SET pulse. (c) READ‐sequence‐dependent volatility for varying numbers of read‐out pulses ranging between 10 and 90 with 1.2 V amplitude, and (d) varying read‐out amplitude from 0.9 to 1.9 V with a fixed number of read‐out pulses. The HRS read‐out points are shown in (d) to account for the different baseline stemming from different READ voltages. The read‐out pulse width is fixed at 1ms. The SET pulse before each decay is always 4.2 V and 20 ms. The normalized current plot from (d), where the difference in the decays are clearly visible, can be found in Supporting Information S18. Current decay measurements from a device with thicker α‐STO (20 nm instead of the 5 nm used in previous measurements) for (e) varying amplitude ranging from 3.0 to 7.0 V with a fixed width of 50 ms, and for (f) 6.0 V SET pulses with different widths ranging from 10 up to 500 ms. The read‐out voltage used is always 1.8 V. (g) Effective time constant range map acquired by the different methods from (a–f). A range of effective time constants from 32 ms to 4.1 s are achieved.

In Figure 4a,b, the stimulus‐dependent relaxation response of the α‐STO devices is shown. By varying the width and amplitude of the SET pulse applied prior to the relaxation, effective decay constants from range 32 to 59 ms are achieved. This tunability arises directly from the ionic nature of the relaxation [62]. In particular, stronger and longer SET pulses drive more oxygen ions out of the oxide layers of the stack, thereby requiring longer times for them to return to equilibrium and resulting in extended current decay.

In Figure 4c,d, we demonstrate how the READ sequence can be used to modify the current relaxation of the devices. Figure 4c depicts the current decay for varying numbers of read‐out points. By increasing the number of read‐out pulses from 10 to 90, the decay becomes longer. The reason behind this behavior is that every read‐out pulse decelerates the backward movement of oxygen ions because a voltage of the same polarity as the SET pulse is applied. Hence, a higher read‐out frequency leads to slower oxygen ion recombination and longer decay times. Similarly, increasing the read‐out pulse voltage results in longer current decay, as illustrated in Figure 4d. It can be observed that higher current levels are also reached at higher read‐out voltages due to the inseparability of ionic motion and electronic conduction. This occurs because of the induced Schottky barrier lowering with increasing voltage. Similar results have also been demonstrated for crystalline STO by [7], where they showed that the decay time could be modulated by applying a constant voltage bias between SET pulses. The current relaxation could be even accelerated under negative applied voltage bias [7].

These results demonstrate that the read‐out sequence can be used to further modulate the device volatility, enabling enhanced behavioral and dynamical complexity. In Supporting Information S18, the normalized currents are depicted to make the difference in decay times more visible for Figure 4d. By varying the READ sequence, effective time constants from range 71 to 184 ms are acquired. As mentioned in Section 2.5, in the event‐based vision application the operation is based on only a single read‐out point, the trigger pulse, after the SET pulse (facilitation pulse). There, the READ‐sequence effect can be ignored, but it becomes crucial once multi‐pulse read‐out sequences are applied. Exactly this property was utilized in Yang et al. [66], where the here presented α‐STO devices were demonstrated as hardware elements for memristive dendrites.

Another way to modulate the current decay is through stack engineering. By increasing the thickness of the α‐STO, much longer decay times can be achieved. A thicker α‐STO can act as a larger oxygen reservoir allowing oxygen ions to move from deeper regions during the SET process. The band diagram and the electric field distribution inside the thicker α‐STO are presented in Supporting Information S19. As it can be seen from the band diagram, the depletion region for the 20 nm α‐STO device extends deeper into the STO, resulting in changes in the electric field that can drive oxygen migration, as calculated in Figure S17b. In [62], the ionic model suggests that longer time constants arise from oxygen migration in the bulk‐facing part of the depletion region. In contrast to the oxygen incorporation process from the Pt into the STO, oxygen migration inside the oxide is much slower due to higher saddle point energies of oxygen migration. To achieve such long time constants, therefore, oxygen must have been depleted from these regions during the resistive switching process.

The longer migration distance may then slow down recombination during relaxation, which can lead to extended decay time constants. In Figure 4e,f, the current decays from a 20 nm thick α‐STO device are presented. By varying the SET pulse parameters as in Figure 4a,b, a large range of effective time constants from 193 ms to 4.1 s is achieved on a single device. A key difference between Figure 4e and Figure 4f, is that the SET pulse width affects the decay time more strongly than the SET pulse amplitude. This could be explained if we consider that the voltage required to switch the device already lowers the oxygen ion migration barriers significantly [62]. Therefore, longer SET pulses allow ion migration to continue for a longer duration, resulting in longer decay times. In addition, these findings provide further insights into the nature of the decaying behavior. This large range of decay times observed in a single device provides additional concrete evidence for the ionic nature of the α‐STO devices, as a purely electronic mechanism cannot account for such behavior. Simply increasing the thickness of the α‐STO is not expected to modify the energetic landscape sufficiently to explain such pronounced differences in decay times.

Further exploring the impact of stack engineering on the current decay, we investigate the role of the top electrode (TE) material by fabricating a device with an Au TE. The comparison between Au and Pt TE devices' switching curves is depicted in Supporting Information S20. The Au TE device exhibits similar switching behavior as the Pt TE devices, with the switching starting at lower voltage while higher currents are observed under reverse bias. This is attributed to the lower work function of Au compared to that of Pt, resulting in a reduced Schottky barrier height at the interface with the α‐STO. As can be seen in Figure S19 (light blue), the Au TE device showcases a long decay for >40 s, after a SET pulse of 3.5 V and 50 ms. We assume that this effect could stem from different oxygen incorporation activation energies or from different migration speeds of oxygen inside the Au compared to Pt [67, 68].

To further investigate the physics behind this behavior, we fabricated a device with a stacked TE consisting of 1nm Pt and 30 nm Au. The current decay from this device is also shown in Figure S19 (dark blue). Interestingly, we observe that the decay now starts with the sharp drop within the first ∼ 200 ms (See inset of Figure S19), resembling a decay from the Pt TE electrode devices, followed by a longer decay for 40 s like in the Au TE devices. This hybrid decaying behavior is achieved by simply stacking different electrode materials, without bringing the Au in contact with the underlying layers. These observations support the hypothesis that ion migration within the electrode plays a key role in the observed behavior and indicate that the TE can be engineered to realize more complex decay dynamics. Overall, these findings open up further possibilities for engineering versatile and tunable volatile memristive devices with short‐ and long‐term memory capabilities [12]. Moreover, these findings provide evidence for the ionic nature of the decay, and indicate that oxygen migration within the TE can be influenced. Consequently, both the recombination rate and the back‐diffusion rate of oxygen should be taken into account when engineering device volatility.

The devices reported here exhibit the broadest range of achievable time constants reported for STO‐based volatile memristive devices (Table S2). Combined with their CMOS BEOL‐compatible fabrication process, this makes them particularly attractive for neuromorphic and edge‐computing applications. After establishing the different ways to tune the current decay of our devices, it is important to demonstrate that this property can be harnessed in a controllable and reproducible manner.

### 2.4 Reproducibility of Current Relaxation

Variability and reproducibility are critical for the event‐based sensing concept introduced in Section 2.5, as it relies on a gradual and highly reproducible current relaxation. Figure 5 presents a detailed reproducibility analysis of our area‐dependent α‐STO devices, revealing their excellent stability and consistency across repeated switching cycles.

**FIGURE 5:**
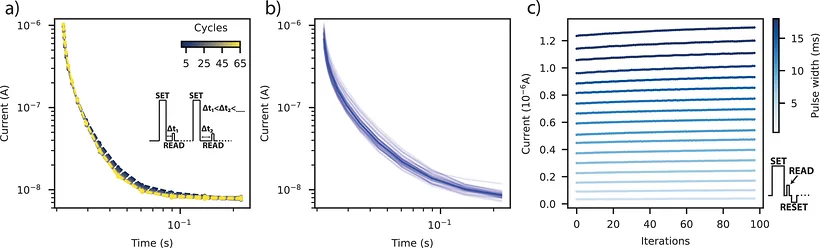
Decay reproducibility of the volatile α‐STO devices. (a) C2C true decay variability for 65 true decay cycles from a single device. The inset depicts the true decay measuring scheme. Instead of a continuous read‐out pulse train after a SET pulse, the decays are reconstructed by sequential single SET and READ pulse segments with a gradually increasing interval between them. Each cycle consists of 15 SET‐READ pulse segments resulting in a total of 975 total pulse segments across all 65 cycles. (b) True decays from 45 different devices on a single chip. The decays are acquired after SET pulses of 4.2 V and 20 ms. (c) Decay states variability measurement from one device. The measurement pulse scheme is depicted next to the plot. The measurement consists of a SET pulse of 4.1 V amplitude and width ranging between 1 and 20 ms, a READ pulse of 1.2 V and a RESET pulse of −0.9 V and 6 ms. The interval between the SET and the READ pulse is fixed at 10 ms and for each SET pulse width the measurement is repeated for 100 times. 17 distinct decay states are achieved and reproduced with no overlapping.

In a circuit, the devices are accessed only momentarily during the decay to assess their state after a defined delay time. To quantify Cycle‐to‐Cycle (C2C) and Device‐to‐Device (D2D) variability under realistic operating conditions, we implemented a “true decay” measurement protocol that closely mimics the readout scheme of an event‐based sensing circuit. Rather than using a continuous readout, we apply a sequence of identical single SET and READ pulse segments at predefined delays and reconstruct the current decay from the resulting discrete samples to test for any increase in variability. The pulse train applied during the measurement is depicted in the inset of Figure 5a. In Figure 5a and Figure  5b, the C2C and D2D true decay variability measurements are shown, respectively. Figure 5a depicts 65 true decay consecutive cycles, that is, 975 SET and READ cycles‐measured on one device, showcasing excellent C2C decay variability. The devices achieve stable operation after some initial cycling of 10 true decay cycles, see Supporting Information S21. We assume, that this behavior stems from the ionic nature of the decay. Specifically, when a device is cycled repetitively, the ionic migration and recombination rates are reaching an equilibrium, which is determined by the SET pulse parameters. The excellent reliability of our devices is confirmed by Figure 5b. The D2D true decay variability from 45 different devices shows tightly clustered behavior, making the α‐STO device suitable for neuromorphic and signal‐processing applications, where uniformity and reliability is a prerequisite.

As showed earlier, the devices can exhibit multiple effective time constants—that is, distinct decay states—and it is therefore important to demonstrate that these states can be selectively accessed, distinguished, and cycled within a single device without overlap. In Figure 5c, by modulating the SET pulse width between 1 and 20 ms, while keeping the SET pulse amplitude constant, we attain 16 different decay states, corresponding to 4 bits. These 16 decay states are cycled for 100 cycles each and monitored with a single READ pulse at a fixed time interval of 10 ms. The observed current difference for different decay states with higher SET pulse width, indicate that the decay becomes longer. Clearly separated decay states are attained, with no overlapping over the 1700 iterations of the measurement. These results, outline the sublime reliability and uniformity of our devices.

The ability to reproducibly access and maintain multiple, well‐separated decay states is a key requirement for employing volatile devices as functional elements in neuromorphic signal processing. In particular, for event‐based applications and implementations of the Time Difference Encoder (TDE), short‐term memory is commonly realized using time‐decaying signals, traditionally implemented with capacitive elements. The high reliability, low variability, and programmability of the decay dynamics demonstrated here indicate that the proposed α‐STO devices can provide a robust alternative substrate for realizing tunable temporal traces, enabling their use in event‐based processing systems explored in the following Section 2.5.

### 2.5 Demonstration of Event‐Based Sensory Processing With Volatile Memristive Devices

Event‐based sensing is a paradigm in which signals are sampled and communicated asynchronously only when changes occur [69], mimicking biological sensory processing [70], and eliminating redundant information transmission while maintaining high temporal fidelity. For example, event‐based cameras, operating in the visual domain, generate asynchronous streams of events in response to pixel‐level changes in luminance [39]. Processing such data for downstream applications like perception‐motor loops in autonomous systems is gaining attention [71, 72], yet remains challenging, as converting events back into frames sacrifices the inherent low‐latency and computation‐efficiency advantages of the paradigm. Spiking Neural Network s (SNNs) offer a natural processing domain, since their event‐driven communication directly matches the temporal characteristics of event camera output.

The TDE [73] has been proposed as primitive computational element that can be embedded within SNN architectures to enable local spatiotemporal sensitivity. This has been leveraged for applications such as obstacle avoidance [74], sound‐source localization [75], optical flow estimation [76], egomotion estimation [77], and odor localization [78]. The model leverages synaptic traces to detect spatiotemporal correlations in event‐based data and has been implemented across various hardware platforms including digital neuromorphic processors [74], Field Programmable Gate Arrays (FPGAs) [79], and analog subthreshold circuits [73, 80]. Analog implementations are particularly attractive for their low power consumption [81] and real‐time processing capabilities, as they exploit device physics to naturally emulate temporal dynamics rather than requiring continuous memory updates as in digital systems [82, 83]. However, scaling analog circuits is challenging, as achieving long time constants typically requires large capacitors, which do not scale favorably with process technology. Replacing these with devices that intrinsically provide long time constants allows for more compact implementations, achieving dynamics beyond what is possible with equivalently sized CMOS circuits.

Figure 6 illustrates how the memristive device introduced in this work can interface directly with the output of an event‐based sensor. This is a simplified representation; a fully realized system would require integration with CMOS technology and interface circuits to integrate the device [84]. Additional infrastructure would also be required to handle the resulting signals, for example circuits that emulate neurons [81] and perform event processing. In this scheme, each volatile device acts as a short‐term memory element whose conductance encodes the recent history of incoming events, naturally decaying over time. The instantaneous device state can therefore signal temporal correlations between events, while routing events across an array of such devices enables the detection of spatiotemporal correlations. In the visual domain, these correlations emerge from events originating from neighboring pixels, whereas in other sensory modalities they may arise across different input channels. Crucially, the programmable volatility of the device, driven by the controlled migration of oxygen ions, allows the correlation time window to be adjusted over a wide range, enabling sensitivity to both short‐ and long‐timescale dependencies. This capability is particularly relevant for modalities such as olfaction, where meaningful correlations can span significantly longer durations [78]. By providing tunable time constants within a single compact device, this approach eliminates the need for large capacitive elements typically required in analog CMOS circuits, avoiding inefficient area usage when long time constants are unnecessary and enabling scalable neuromorphic implementations.

**FIGURE 6:**
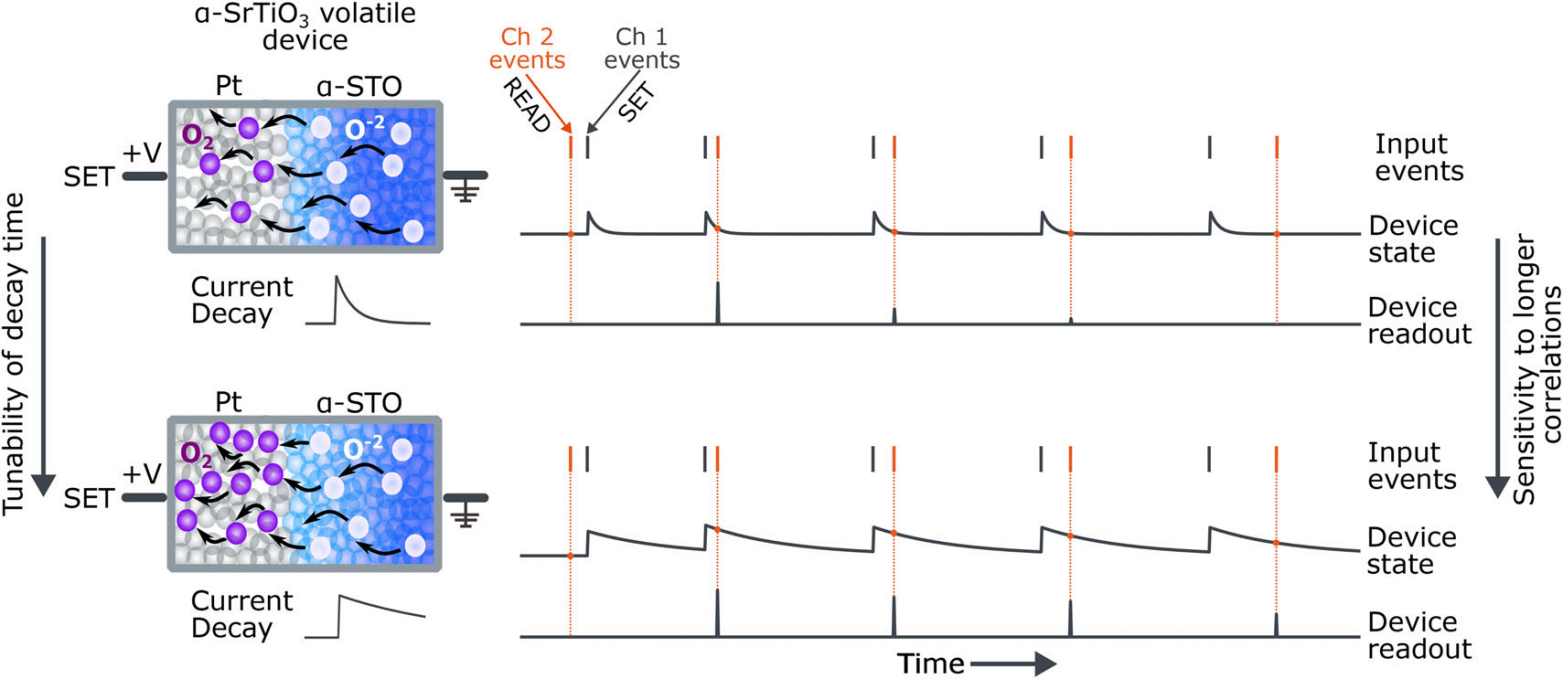
Event‐based sensing with tunable volatile α‐STO‐based devices. The ionic‐based tunable volatility of the device can be used to acquire different typical time correlations of input events. Increased oxygen ion migration into the Pt during SET leads to longer subsequent current decay and enhances the device's sensitivity to longer correlations.

Building on the temporal dynamics and reproducibility of the volatile memristive devices demonstrated above, we next investigate their use as functional elements in neuromorphic event‐based processing. Specifically, we implement a memristive realization of the TDE, hereafter referred to as the memristive TDE, in which the devices' intrinsic volatility is exploited to encode temporal correlations between asynchronous input events. The memristive TDE operates by communicating the time difference between two input events, this is achieved using two inputs: a *facilitation* (FAC) input that modulates the device state, and a *trigger* (TRG) input that probes this state (Figure 7a). The resulting current reflects the temporal separation between these inputs, such that correlations in time are transformed into a response (Figure 7b) proportional to their operation. This current can then be fed into a spiking neuron to elicit a spiking response, which encodes the time difference between the events input a spike rate. Figure 7c illustrates a single demonstrative example, where a randomly generated sequence of FAC and TRG input events was applied to the device and the system response was measured. It can be observed that correlated inputs, specifically TRG events occurring shortly after FAC events, elicit spiking activity from the simulated neuron. To apply the memristive TDE computational unit to an event‐based vision task, a bar was simulated moving across the sensor with increasing speeds. In this experiment, the time differences (Δt) between events occurring at neighboring pixels, generated by the moving bar, were not uniform but instead followed a distribution (Figure S21) as a result of simulated sensor noise and pixel‐level jitter. Figure 8a illustrates how this principle scales to a larger scene: a layer of many memristive TDE units aligned to left–right motion of the bar was distributed densely across the field, totaling 90 units. Initial experiments were conducted using a single device and a simulated 10 × 10 pixel sensor. The simulation was run sequentially on a single device, with different event inputs reflecting the location of the memristive TDE unit in the visual field. Figure 8b shows that the average current sourced by the device increased systematically with the bar's speed (measured in pixels per second).

**FIGURE 7:**
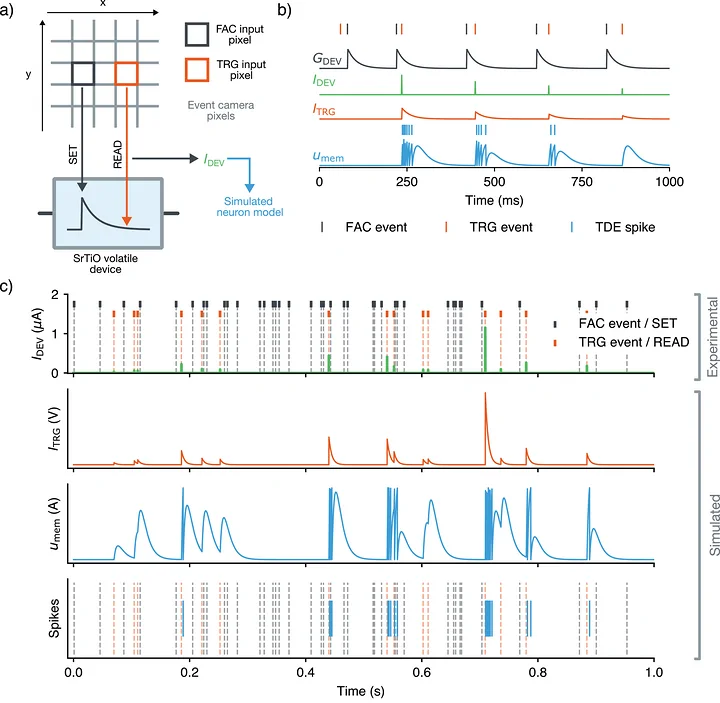
The use of the device as a circuit element to encode the spatiotemporal correlation of events from an event camera. (a) The facilitation (FAC) input elicits a SET operation on the device, whereas a trigger (TRG) input elicits a READ operation. (b) The current sourced by a TRG input (READ) is proportional to the device conductance $G_{\text{dev}}$ at that time, producing a current $I_{\text{DEV}}$. This current in turn generates a postsynaptic current $I_{\text{TRG}}$ (simulated), which stimulates a spiking neuron (also simulated). (c) A single example of this setup tested experimentally on a device.

**FIGURE 8:**
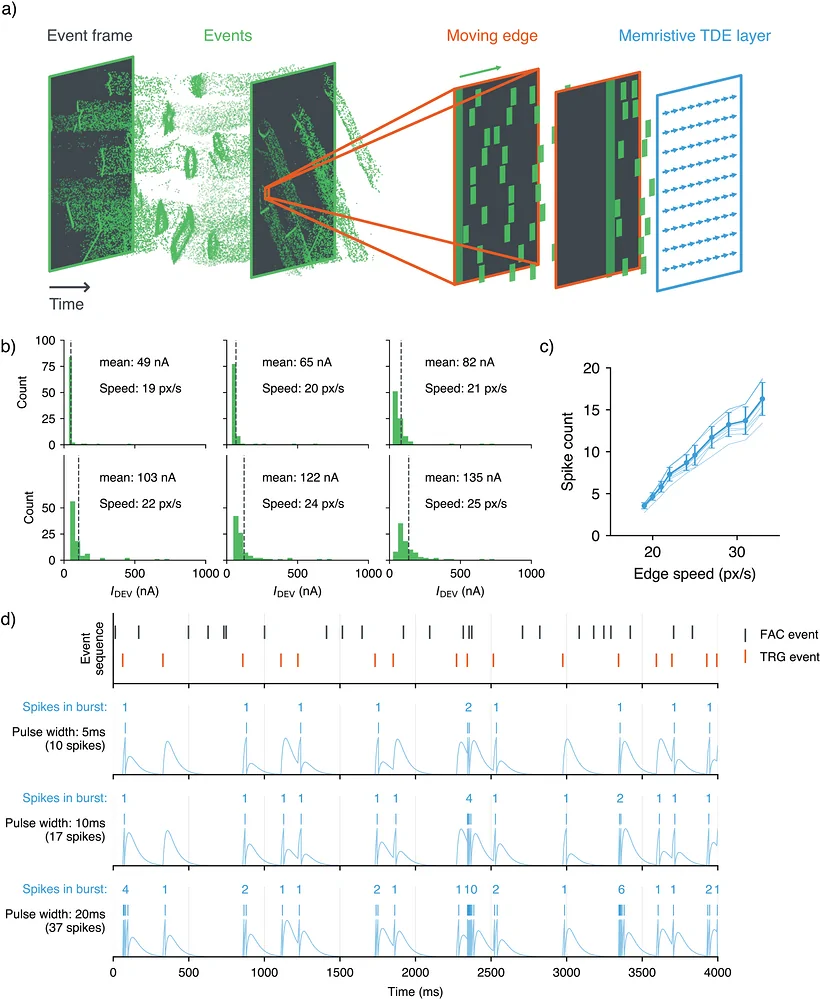
Event‐based sensing experiments. (a) Event‐based cameras produce an event stream that communicates pixel‐wise changes in relative brightness. This produces “moving edges” of objects in a dynamic scene, we can use a layer of memristive TDE units orientated to have a preference direction to be responsive to directions of motion. (b) Histograms showing experimental data of the current reads during the emulation of an edge moving in front of the event‐based camera for varying edge speeds measured in pixels per second (px/s). (c) The memristive TDE spike count against the edge speed for eight devices in an array. (d) Demonstration of device time constant tunability. The same input event sequence was applied to a single device while varying the pulse width of the FAC (SET) pulses. Longer pulse widths extend the temporal window over which the device can discern time differences, enabling tunable temporal sensitivity. At higher pulse widths, the neuron continues to elicit responses at longer time differences and produces a greater number of spikes per burst at shorter time differences.

This demonstrates that the device does not simply respond to isolated events, but instead encodes the relative timing of events across the scene, producing a signal proportional to motion velocity. To evaluate scalability and D2D variation, the experiment was repeated using eight devices on the same input event stream. Figure 8c shows individual device responses and their average, revealing modest variation across devices but consistent population‐level encoding of the bar's velocity. Together, these results show that a system composed of many memristive TDE processing units can be tiled across a field of view to form a distributed motion‐sensitive layer for encoding visual motion.

While the example above focuses on event‐based visual sensing with a stereotypical correlation time constant on the order of milliseconds, the same principle can be extended to other sensory modalities such as audition, olfaction, and touch. These modalities exhibit characteristic correlation times spanning a broad temporal range. To adapt the memristive TDE to different tasks, the SET (FAC) input can be modulated to tune the device's effective time constant, either increasing or decreasing it depending on the required temporal sensitivity. This flexibility applies even within the visual domain, where detecting slower motion would necessitate a longer integration time constant. To demonstrate this tunability, we generated a sequence of FAC and TRG events with a quasi‐uniform distribution of time differences between 0 and 200 ms, then applied this sequence to the same device three times while modulating the pulse width of the SET (FAC) pulse. As shown in Figure 8d, longer pulse widths render the memristive TDE more receptive to longer time differences, indicating that the temporal sensitivity can be input‐driven and modulated for different applications, see Figure S23 for full sequence.

The tunability of the device dynamics also introduces an inherent trade‐off between temporal integration and event discrimination. The device decay time determines the temporal integration window over which preceding FAC events influence the conductance sampled by subsequent TRG events. Therefore, when the average interval between FAC events is shorter than the device relaxation time, the sampled conductance reflects the cumulative influence of multiple recent events rather than only the most recent event. Longer decay times extend the correlation window and are beneficial for sparse or slowly varying event streams, whereas shorter decay times improve temporal selectivity for high‐rate event streams. Consequently, the ability to modulate the device dynamics through input parameters provides a means to tailor the temporal response to the event statistics of the target sensing application.

These results establish the use of volatile α‐STO‐based devices for event‐based sensing, and in particular highlight their capacity to modulate the temporal sensitivity of spiking neuron models through input pulse parameters. A promising direction for future work would be the realization of a CMOS‐integrated system incorporating BEOL‐compatible devices, enabling scaled experiments, further investigation of interfacing circuitry, and exploration of additional event‐based sensing modalities.

## 3 Conclusion

Overall, we presented an area‐dependent volatile Pt/α‐STO/${\mathrm{TaO}}_{x}$/Ta memristive device whose temporal dynamics are both physically understood and practically tunable. The device stack presented here is, to the best of our knowledge, the only established α‐STO‐based memristive system exhibiting volatile switching, making it particularly attractive for applications due to its CMOS BEOL‐compatible fabrication process, as summarized in Table S1. Through temperature‐dependent transport measurements and systematic stack engineering experiments, we identified the dominant transport and decay mechanisms. We confirm the applicability of the physical model previously used for c‐STO‐based devices, to interpret the current decay of our devices and support this with quantitative fittings. This understanding enables deliberate tuning of the decay using either stimulus parameters or stack design, allowing a single device to cover different temporal operating regimes. Importantly, the device exhibits low variability and strong reproducibility across multiple, distinct decay states, addressing a key barrier for deploying volatile memristive elements in circuits. Moreover, we demonstrated event‐based sensing functionality through a memristive time‐difference encoder and showed how tunable decay provides selectable temporal‐range sensitivity, enabling more efficient and capable temporal processing circuits.

Together, these results establish a systematic framework for controllable and reproducible volatility in area‐dependent memristive devices. The physical insights and demonstrated pathways to engineer the temporal dynamics could facilitate the development of a variety of devices exhibiting complex volatility. These findings open opportunities for designing versatile temporal processing elements and could potentially enable applications across a broader range of dynamic computing paradigms.

## 4 Methods

### 4.1 Device Fabrication

A commercially available thermally oxidized Si wafer with 430 nm. ${\mathrm{SiO}}_{2}$ was diced into $10\times 10\;mm^{2}\;$ substrates using a dice tape for surface protection. On top of a Si/${\mathrm{SiO}}_{2}$ substrate, a 40 nm Ta layer was first deposited by RF Magnetron sputtering using 30 W RF power, at room temperature and at 0.01mbar Ar chamber pressure, as the bottom electrode. Subsequently, 5nm of α‐STO were deposited by PLD at 300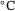 and 0.1 mbar $O_{2}$ pressure, by ablating a single crystal STO target with a laser energy of 1.05 J ${\mathrm{cm}}^{-2}$ and a repetition rate of 5 Hz. The elevated temperature and $O_{2}$ pressure resulted in the growth of a native ${\mathrm{TaO}}_{x}$ layer of around 3.5 nm between the Ta and the α‐STO layers. The 30 nm Pt top electrode was deposited by e‐beam evaporation through a shadow mask to achieve the square patterned pads of 10×10, 50×50, 100×100, and $200\times 200\;\mu m^{2}\;$ size. The sample was mounted on top of a carrier wafer with Pt pads and wire bonding was used to access the Ta bottom electrode.

The Pt/Ta/α‐STO/${\mathrm{TaO}}_{x}$/Ta was fabricated in the same way as the other α‐STO devices with 5 nm α‐STO. Instead of evaporating 30 nm Pt as the TE, 30 nm of Ta were sputtered using 30W RF power, at room temperature and at 0.01 mbar Ar chamber pressure and subsequently, without braking the vacuum, 20 nm Pt were sputtered on top using 20W DC power, at room temperature and at 0.01 mbar Ar chamber pressure. The Pt acts as a passivation layer to avoid the oxidation of the Ta. The patterning of the TE was done with positive lithography and Reactive Ion Beam Etching.

For the Pt/Ta/${\mathrm{Ta}}_{2}O_{5}$/Ta sample, 30 nm Ta were sputtered using 30 W RF power, at room temperature and at 0.01 mbar Ar chamber pressure on top of a thermally oxidized Si substrate. Subsequently, 3.5 nm of full ${\mathrm{Ta}}_{2}O_{5}$ were sputtered with 200 W RF power, at room temperature and at 0.05 mbar Ar:$O_{2}$ = 3:2 chamber pressure. Thereafter, another 30 nm Ta and 20 nm Pt were sputtered to form the top electrode. All the layer depositions were conducted without braking the vacuum. The patterning was done with positive lithography and Reactive Ion Beam Etching.

For the Pt/α‐STO/Nb:STO stack, 5 nm of α‐STO were deposited by PLD in the same way as for the previous samples, on top of a commercially available (100) single crystal Nb:STO (0.5 wt%) substrate. The 30 nm Pt top electrode was deposited by e‐beam evaporation through a shadow mask to achieve the square patterned pads of 10×10, 50×50, 100×100, and $200\times 200\;\mu m^{2}\;$ size.

### 4.2 Electrical Measurements

Both the DC and pulsed electrical measurements were conducted on a manual probe station. Tungsten tips were used to contact the top and bottom electrode. For the *I*–*V* DC sweeps, a single channel source‐measure unit (SMU) Keithley 2611A is utilized. The pulsed measurements are conducted using a high‐performer parameter analyzer Keithley 4200A‐SCS. It is equipped with two SMUs and two pulse‐measure units (PMUs) with remote amplifier and switch modules (4225‐RPM).

### 4.3 Structural Characterization

The crystal structure of α‐STO and thickness of the films were characterized by Grazing Incidence X‐Ray Diffraction and X‐Ray Reflectometry measurements, respectively, using the D8 DISCOVER multi‐purpose x‐Ray diffractometer from Bruker AXS GmbH. The system features a Goebel mirror and a Cu anode for $K_{\alpha 1}$ radiation with a wavelength of 1.54 Å, while for the measurements no monochromator was used.

### 4.4 STEM‐EDX Characterization

Local chemical characterization of cross sectional specimens of the device was performed using a Thermo Fisher Scientific Titan Low Base 60‐300 transmission electron microscope (TEM) operated at 300kV. It is equipped with a high‐brightness Schottky field emission gun (X‐FEG), a Wien‐filter monochromator, an Oxford Instruments Ultim Max TLE 100 EDX, and a CETCOR corrector for sub‐Ångström resolution in STEM mode. HAADF imaging in STEM was performed to image the device stack cross‐section, while elemental maps were extracted from STEM‐EDX spectrum images.

The cross sectional TEM specimen for the cross‐sectional STEM study was fabricated using a Thermo Fisher Scientific Helios 650 Nanolab dual beam microscope, equipped with an ion column. The final thinning and polishing of the TEM lamella was performed at a current of <7pA and a voltage of 5 kV to minimize sample amorphization and structural damage.

### 4.5 Memristive TDE

The TDE incorporates a gated synapse with two distinct traces: a Facilitatory Trace (FAC) and Trigger Trace (TRG) and a Leaky Integrate and Fire (LIF) neuron. The FAC input events are integrated onto the trace $I_{\text{FAC}}$, with dynamics given by,

(1) $$\frac{dI_{\text{FAC}}}{dt}=-\frac{I_{\text{FAC}}}{\tau_{\text{FAC}}}+\sum_{i}\delta \left(t-t_{i}^{\text{FAC}}\right)$$

where $\tau_{\text{FAC}}$ is the time constant of the decay, $\delta (t-t_{i}^{\text{FAC}})$ is a FAC input event at time $t_{i}^{\text{FAC}}$.

The TRG input events are similarly integrated, but the effective weight of TRG events is determined by the current value of the FAC trace,

(2) $$\frac{dI_{\text{TRG}}}{dt}=-\frac{I_{\text{TRG}}}{\tau_{\text{TRG}}}+I_{\text{FAC}}\left(t\right)\sum_{i}\delta \left(t-t_{i}^{\text{TRG}}\right)$$

where $\tau_{\text{TRG}}$ is the time constant of the decay, $\delta (t-t_{i}^{\text{TRG}})$ is a TRG input spike at time $t_{i}^{\text{TRG}}$, and $I_{\text{FAC}}\left(t\right)$ is the value of the FAC trace at time t. The TRG trace serves as an Excitatory Post Synaptic Current (EPSC) into a LIF neuron with time constant $\tau_{m}$ and firing threshold $u_{\theta }$,

(3) $$\frac{du_{\text{mem}}}{dt}=-\frac{u}{\tau_{m}}+\frac{I_{\text{TRG}}}{C_{\text{mem}}}$$

where u is the simulated neuron's membrane potential. When u exceeds the firing threshold $u_{\theta }$, a spike is produced and u is reset to 0. This mechanism enables the detection of temporal correlations between the two input channels, with the neuron's spiking activity encoding the temporal relationship between input events. We demonstrate a proof‐of‐concept implementation where our proposed devices replace the multiple transistors and large silicon real estate typically required for the TDE synapse [73]. The devices' natural decay characteristics act as the facilitation variable, while TRG events sample the device state through current sourcing. The $I_{\text{FAC}}$ trace is replaced by the dynamic resistance R(t) of the device. Upon the arrival of a TRG event at time $t_{i}^{\text{TRG}}$, a current,

(4) $$I_{\text{DEV}}\left(t\right)=G_{\text{DEV}}\left(t\right)V_{\text{TRG}}$$

is sourced, where $V_{\text{TRG}}$ is the applied trigger (READ) voltage. This current is fed into a simulated exponential synapse,

(5) $$\frac{dI_{\text{TRG}}}{dt}=-\frac{I_{\text{TRG}}}{\tau_{\text{TRG}}}+I_{\text{DEV}}\left(t\right)\sum_{i}\delta \left(t-t_{i}^{\text{TRG}}\right)$$

producing an exponentially decaying postsynaptic current proportional to $I_{\text{DEV}}\left(t\right)$. The resulting signal is integrated by a simulated spiking neuron

(6) $$\frac{du}{dt}=-\frac{u_{\text{mem}}}{\tau_{m}}+\frac{I_{\text{DEV}}}{C_{\text{mem}}}$$

In a realized hardware system, the synapse and neuron dynamics could be implemented with CMOS circuits [85, 86]. The magnitude of $I_{\text{DEV}}\left(t\right)$ directly reflects the temporal, separation between FAC and TRG events, thereby encoding spatiotemporal correlations in the input event streams.

Event‐based data were generated by simulating a moving bar across a 10×10 pixel sensor. This was done for 10 different bar speeds, corresponding to progressively shorter time differences between neighboring events triggered along the direction of motion, see Figure S21. Devices were stimulated using SET pulses of 4.2 V and 20 ms, while READ pulses had an amplitude of 1.4 V with the same pulse width. Experiments were conducted on eight different devices on the same die, each with a size of $100\times 100\;\mu m^{2}$. For the example measurement shown in Figure 7c, SET pulses of 4.5 V and 5 ms were used, and READ pulses had an amplitude of 1.3 V on a device sized $50\times 50\;\mu m^{2}$. The simulated trigger synapse had a time constant $\tau_{\text{TRG}}=5ms$. The simulated LIF neuron had a membrane time constant $\tau_{m}=50ms$, membrane capacitance $C_{\text{mem}}=1pF$, and a spike threshold $u_{\theta }=25mV$.

For the tunability modulation experiments in the context of event‐based sensing, we generated a 10 s sequence of events by drawing time differences from a uniform distribution between 0 and 200 ms. We then measured these sequences on the same device while varying the pulse width of the SET pulse (FAC). The current response was used to stimulate a simulated neuron as described previously. The simulated trigger synapse had a time constant $\tau_{\text{TRG}}=2ms$. The simulated LIF neuron had a membrane time constant $\tau_{m}=50ms$, membrane capacitance $C_{\text{mem}}=1pF$, and a spike threshold $u_{\theta }=50mV$. SET pulses had an amplitude of 4.0 V and READ pulses an amplitude of 1.3 V. For interpretation of the measurements, the sequences were synchronized to account for longer pulse widths; unsynchronized measurements are shown in Figure S22.

## Conflicts of Interest

The authors declare no conflicts of interest.

## Supporting information


**Supporting File**: adma74813‐sup‐0001‐SuppMat.pdf.

## Data Availability

The data that support the findings of this study are available from the corresponding author upon reasonable request.
