# Supplementary material for: Volatile Memristive Devices With Tunable Temporal Dynamics For Event‐Based Sensing
Source: Adv Mater. 2026 Aug 30;38(59):e74813. doi: 10.1002/adma.74813 (PMC13689917; doi:10.1002/adma.74813)
Supplement: Supplementary file 1 [file ADMA-38-e74813-s001.pdf]

Supplementary Material

S1 X-ray Reflectivity

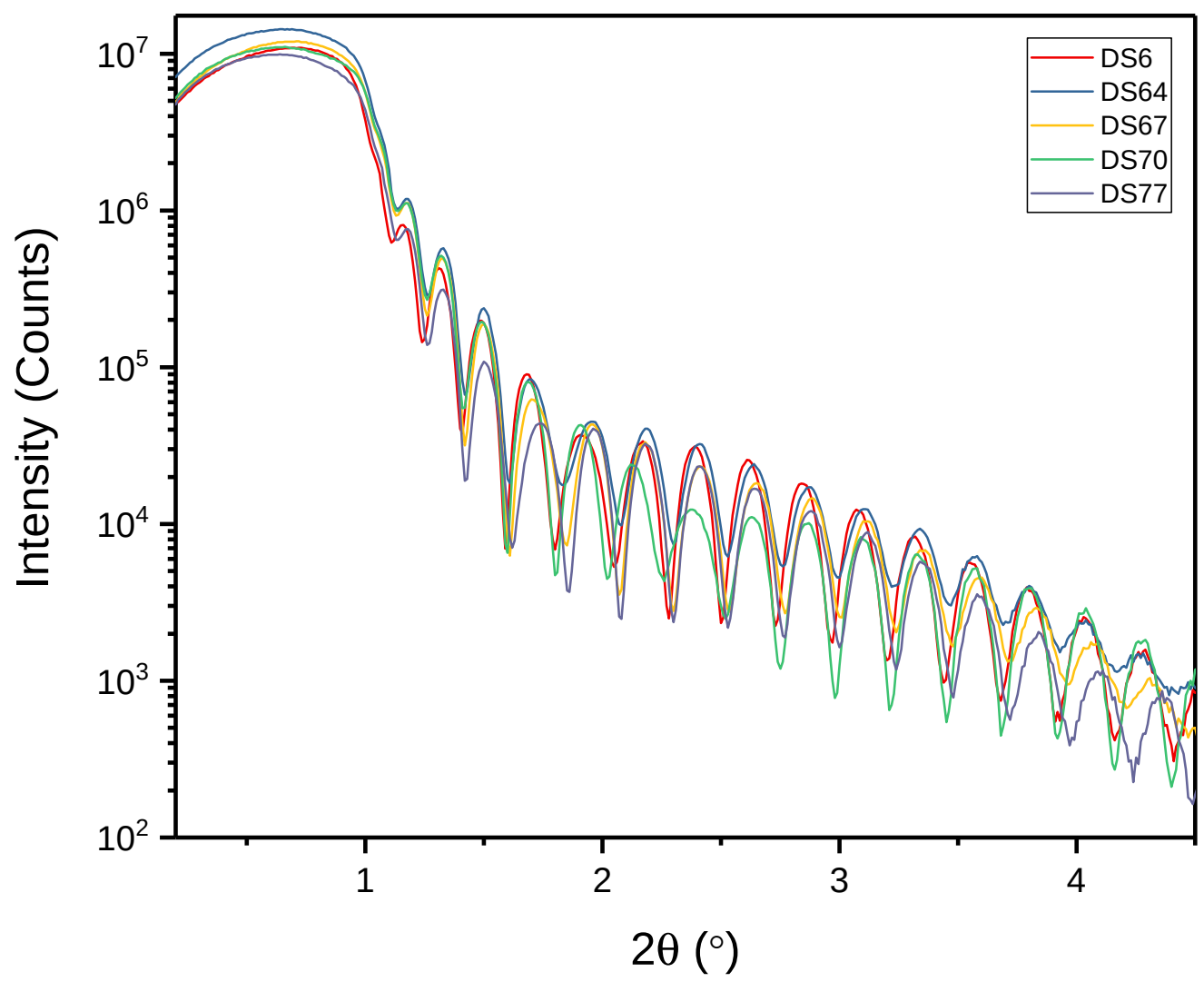

Figure S1: **X-Ray reflectivity.** Measurements from 5 different 5 nm α-STO device samples. The measurement is conducted before the TE deposition because of the high reflectivity of Pt. As it can be seen, all the XRR spectra exhibit similar characteristics, with only minor differences-primarily in the oscillation amplitude-attributed to slight variations in layer roughness. By fitting the measurements, layer thicknesses of approximately 5, 3.5 and 35 nm can be extracted for the α-STO, TaO_x and Ta layers respectively. The samples were fabricated at different times over the span of 1.5 years, demonstrating reproducibility of the fabrication process.

S2 EDX Line profile

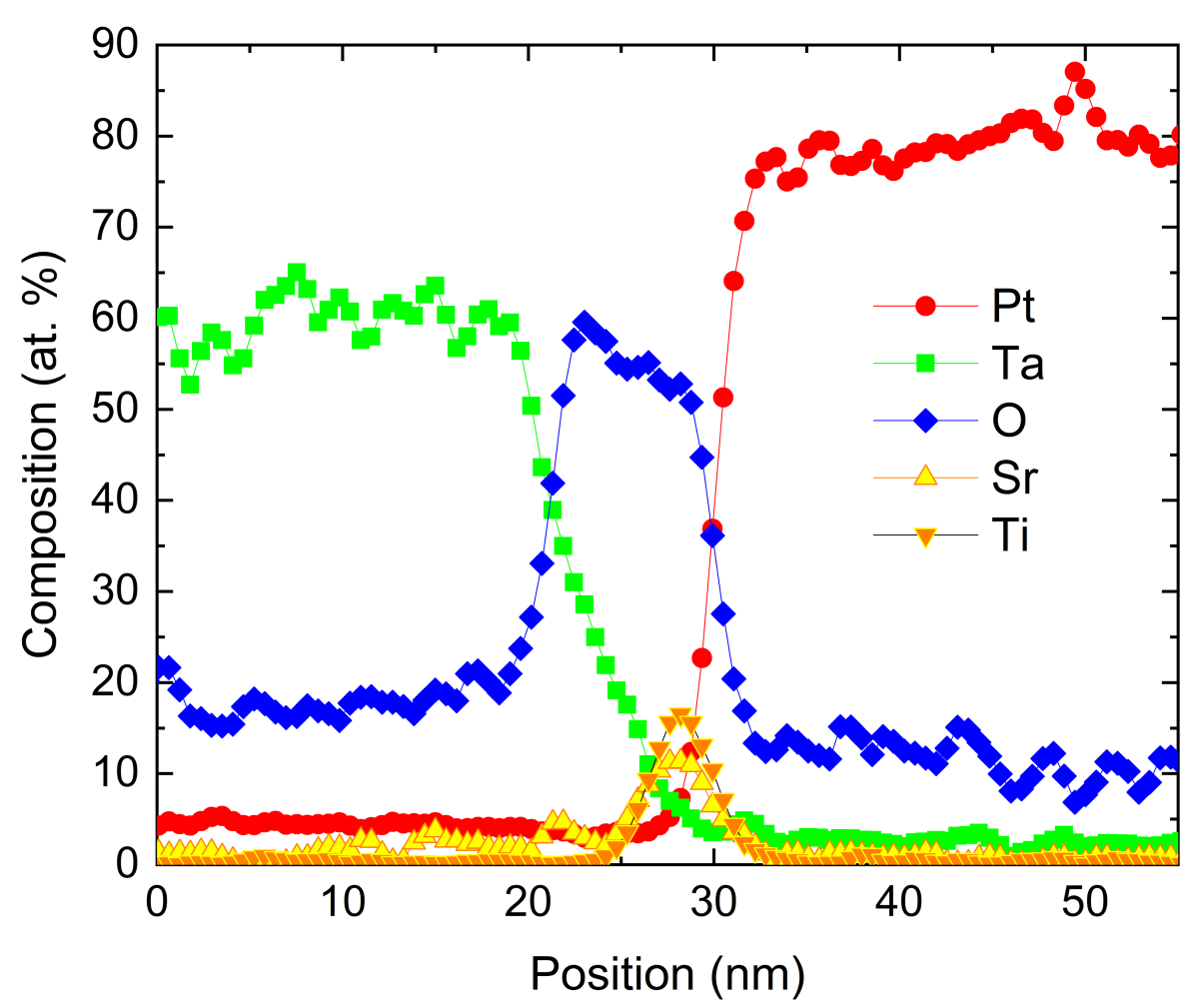

Figure S2: **EDX line profile** Corresponding EDX line profile extracted from Fig. 1c, showing the atomic percentage of Pt, Sr, Ti, Ta, and O as a function of position across the HAADF-STEM image shown in Fig. 1b.

S3 Grazing Incidence X-ray Diffraction

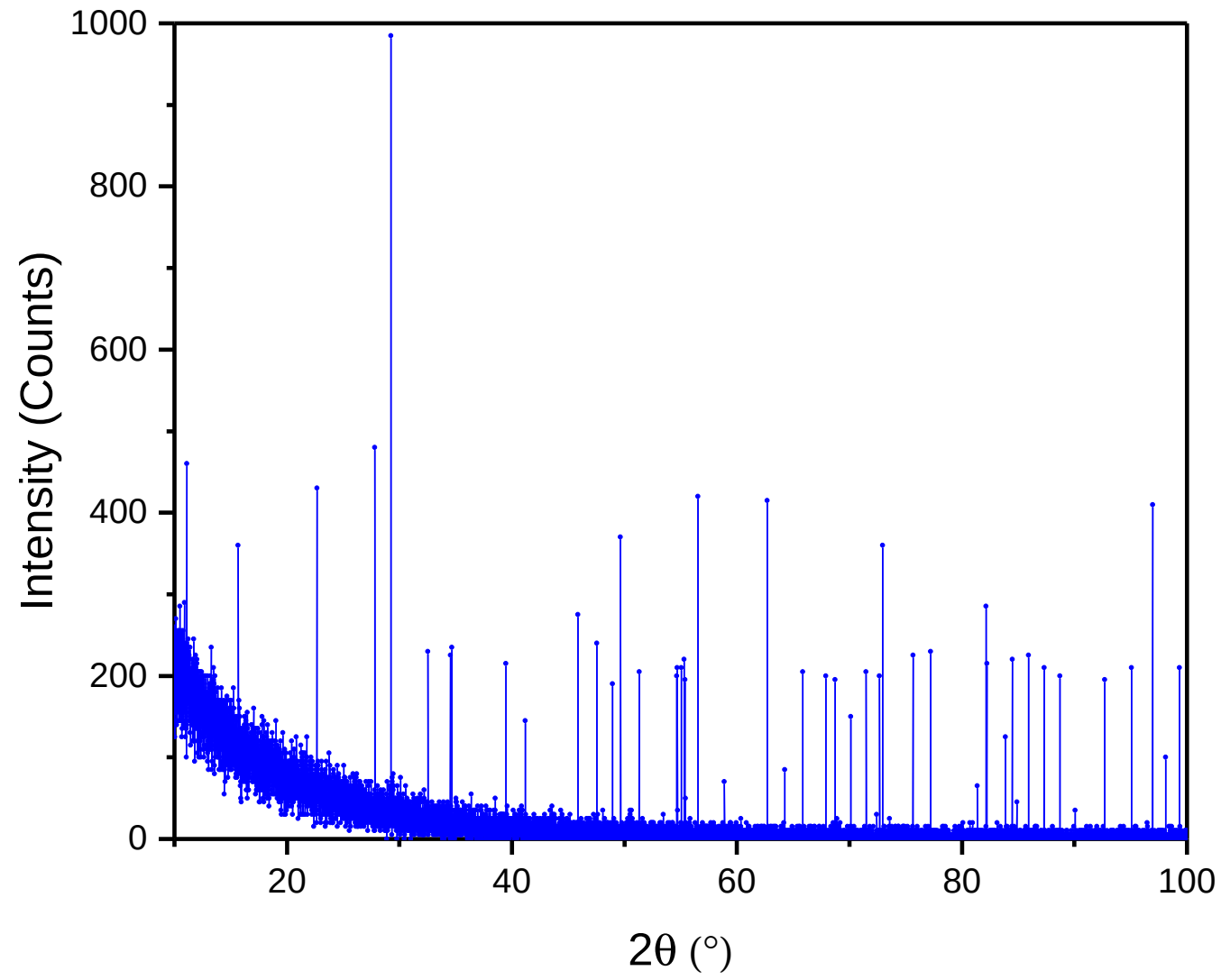

Figure S3: **Grazing Incidence X-ray Diffraction measurement on single α-STO film grown on SiO_2** The amorphous structure is confirmed by the absence of diffraction peaks. The small features observed in the plot are single point noise peaks.

S4 Device-to-device variability

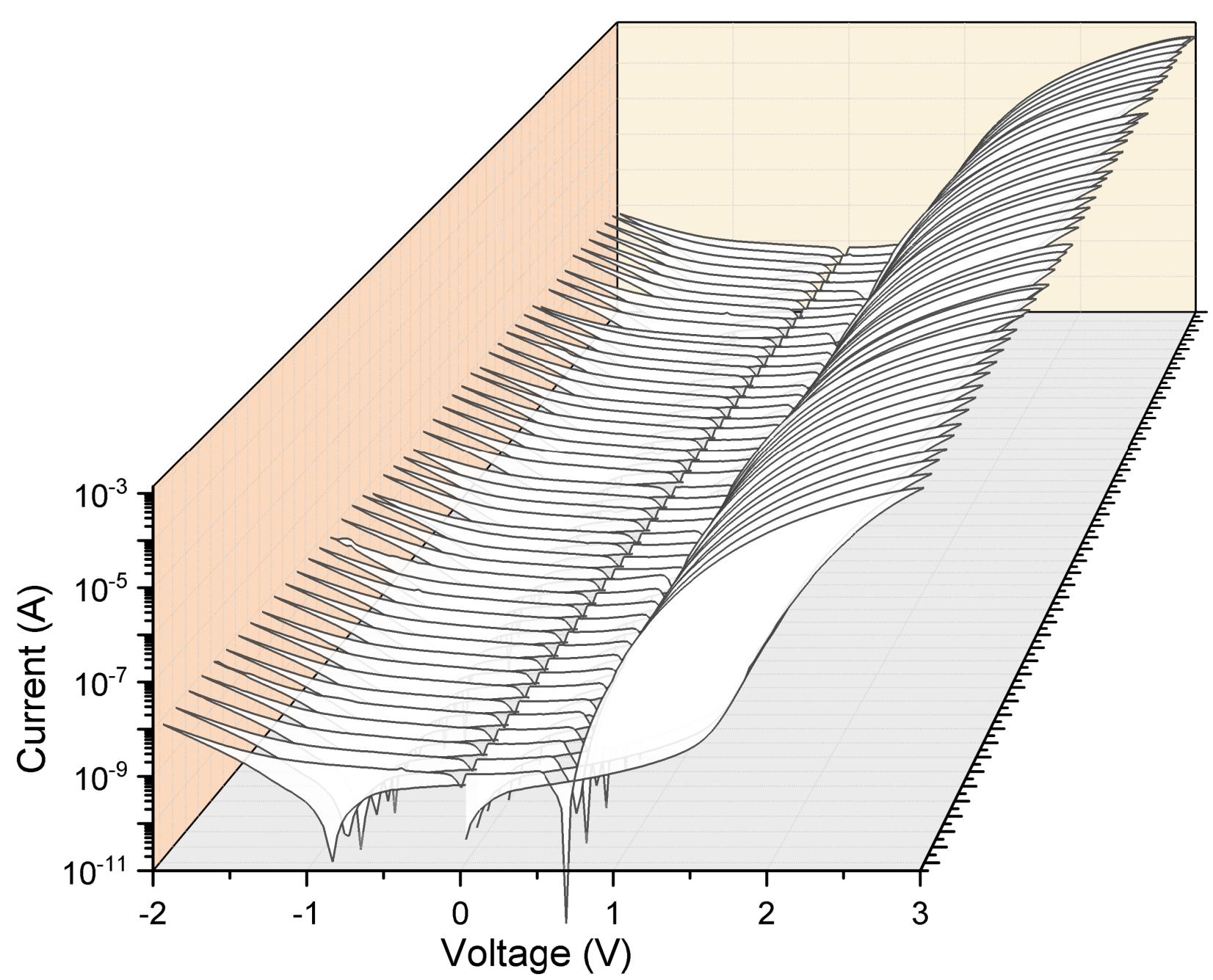

Figure S4: **I-V DC sweeps.** I-V DC sweeps from 50 different devices of $100 \times 100\,\mu m^2$ size. The voltage sweep ($0\,V$ to $3\,V$ to $-2\,V$ to $0\,V$) is applied on the Pt top electrode. The devices show very good uniformity.

S5 Area dependency

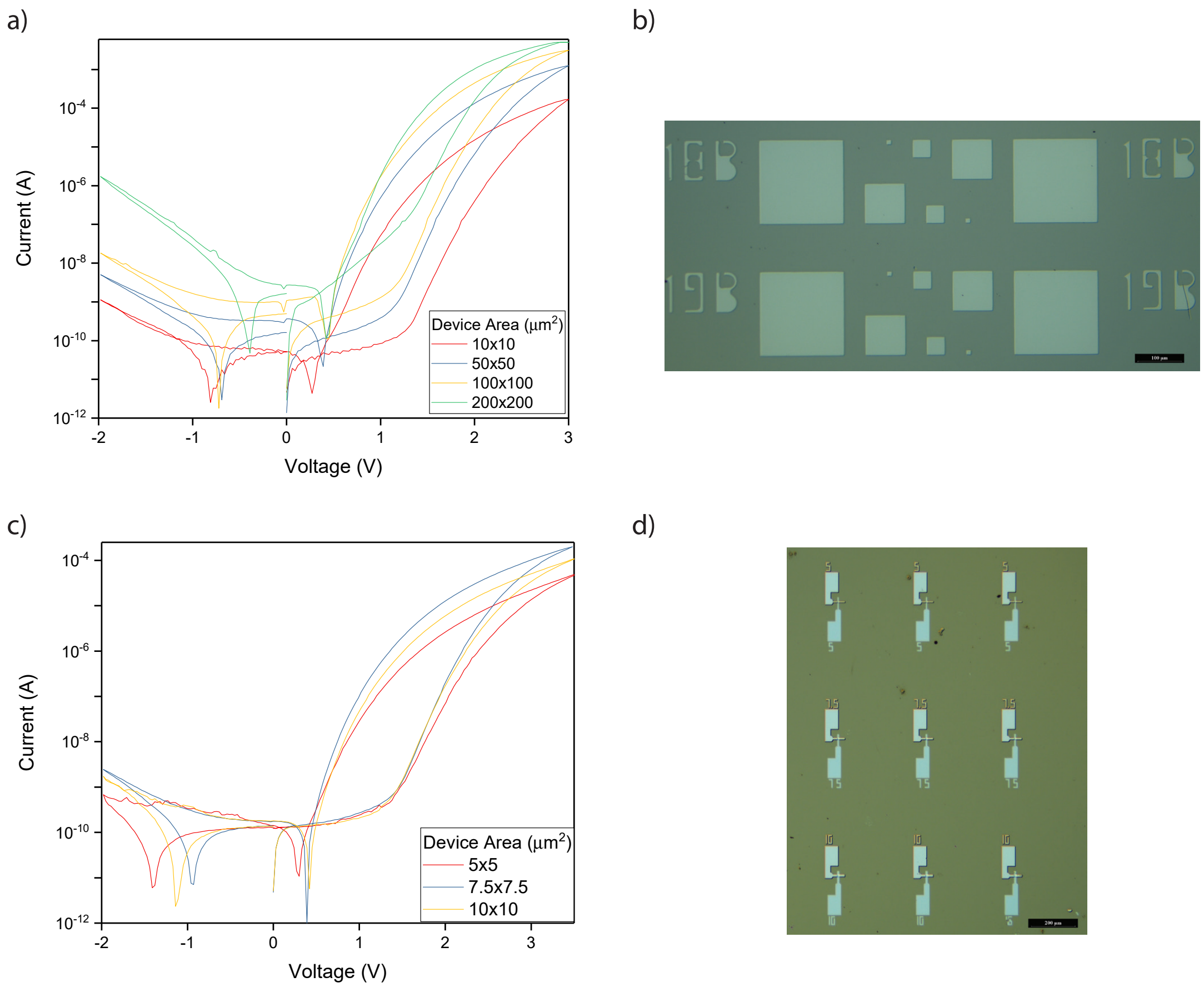

Figure S5: **Area dependent I-V plots.** I-V plots from a) normal pad devices of 10 × 10, 50 × 50, 100 × 100 and 200 × 200 µm^2 device area and from c) single crossbar devices of 5 × 5, 7.5 × 7.5, 10 × 10 µm^2 device area. Optical microscope images of the fabricated b) normal pad device sample and d) single crossbar device sample. The decay measurements presented in this work are conducted on the normal pad devices.

S6 State of the art STO-based memristive devices

Device Stack	STO Crystallinity	Switching	Volatility	Retention	Suggested Mechanism
Cr/Pt/STO/Ti [1]	crystalline	8w, filamentary	volatile	$10^{-1} - 10^{1}$ s	ionic
Al/Pt/STO/Nb:STO [2]	crystalline	8w, filamentary	volatile	$10^{2} - 10^{4}$ s	ionic
Ni/Nb:STO/Ti [3]	crystalline	8w, filamentary	volatile	$10^{3} - 10^{4}$ s	ionic
Au/Cr/STO/Nb:STO [4]	crystalline	8w, filamentary	volatile	$10^{1} - 10^{2}$ s	ionic
Au/Nb:STO [5]	crystalline	8w, area-dependent	volatile	$> 3 \times 10^{4}$	electronic
Pt/Ti/Nb:α-STO/Pt [6]	amorphous	c8w, filamentary	non-volatile	$> 10^{4}$ s	ionic
Pt/Ti/Cr:α-STO/Pt [7]	amorphous	complementary, 8w & c8w, filamentary	non-volatile	$> 10^{5}$ s	ionic
Pt/Nb:STO [8]	crystalline	8w, area-dependent	volatile	$\sim 10^{2}$ s	ionic
Au/STO/LSMO [9]	crystalline	c8w, area-dependent	non-volatile	$> 10^{4}$ s	ionic
Pt/STO/Nb:STO [10]	crystalline	8w, filamentary	volatile	$10^{0} - 10^{1}$ s	ionic
**Pt/α-STO/TaO$_x$/Ta**	**amorphous**	**8w, area-dependent**	**volatile**	$10^{-2} - 10^{1}$ **s**	**ionic**

Table S1: **Comparison of STO-based memristive devices reported in the literature.** Eleven material systems are compared with respect to their device stack, STO crystallinity, switching polarity and mode, retention characteristics, and the physical mechanisms proposed by the authors. Reference entries are listed directly after the stack notation. Here, "8w" denotes a SET event induced by a positive voltage applied to the noble-metal electrode, whereas "c8w" denotes the opposite polarity. Complementary switching refers to threshold switching that exhibits hysteresis only above a certain voltage bias; below this threshold, the hysteresis disappears. The device stack investigated in this work is noted in bold.

S7 Endurance without RESET

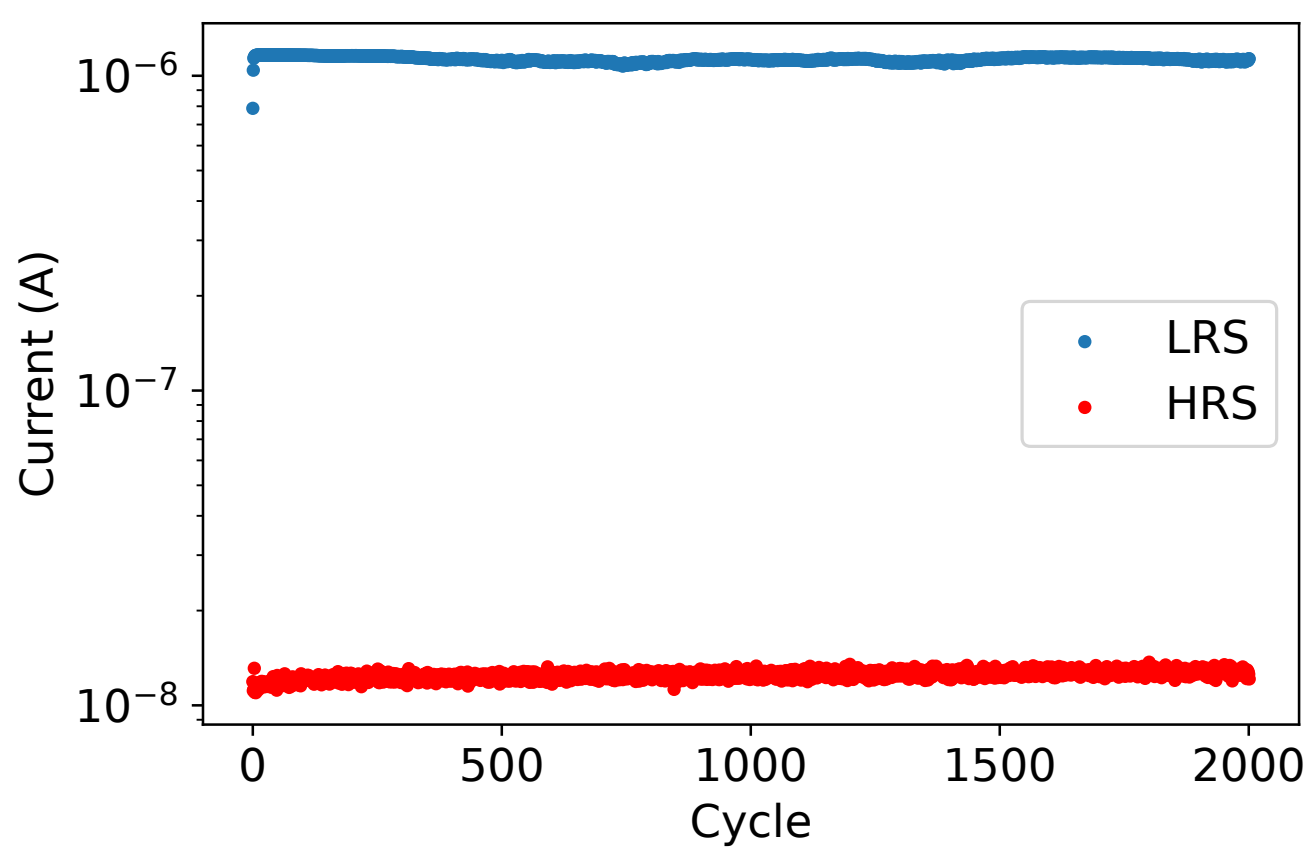

Figure S6: **Endurance measurement without RESET pulses.** Endurance measurement over 2000 cycles consisting of a SET operation followed by complete device relaxation. The SET pulses had an amplitude of 4.0 V and a duration of 30 ms. Read-out was performed at 14 ms after the SET pulse for the LRS and at 1.3 s after the SET pulse for the HRS, using a voltage of 1.2 V.

S8 SET dynamics c-STO

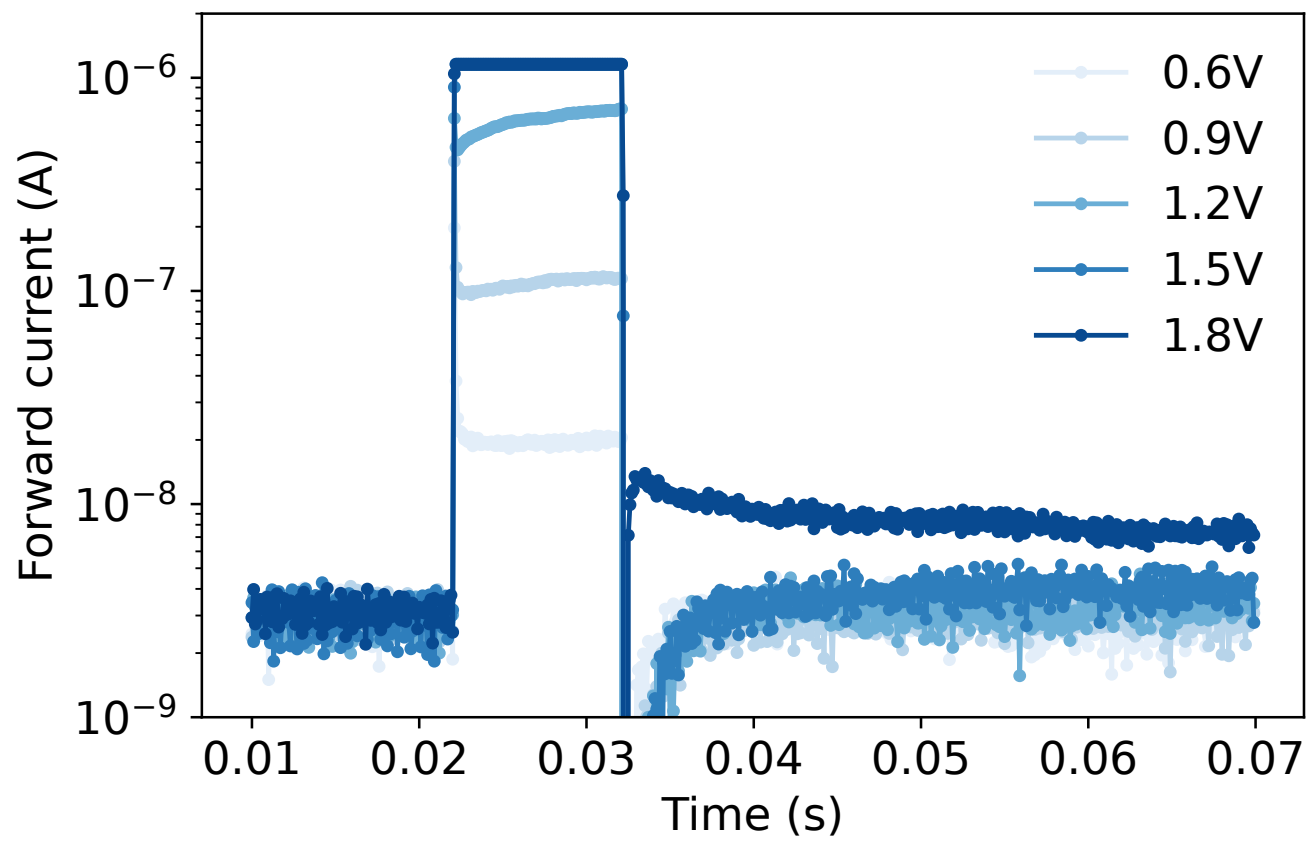

Figure S7: **SET dynamics of the Pt/c-STO/Nb:STO device.** The current during a voltage sequence consisting of a 10 ms READ period of 0.3 V, a 10 ms voltage pulse between 0.6 V up to 1.8 V followed by a 40 ms READ period of 0.3 V. The maximum measuring range is 10^{-6} A.

S9 Bulk conduction of α-STO

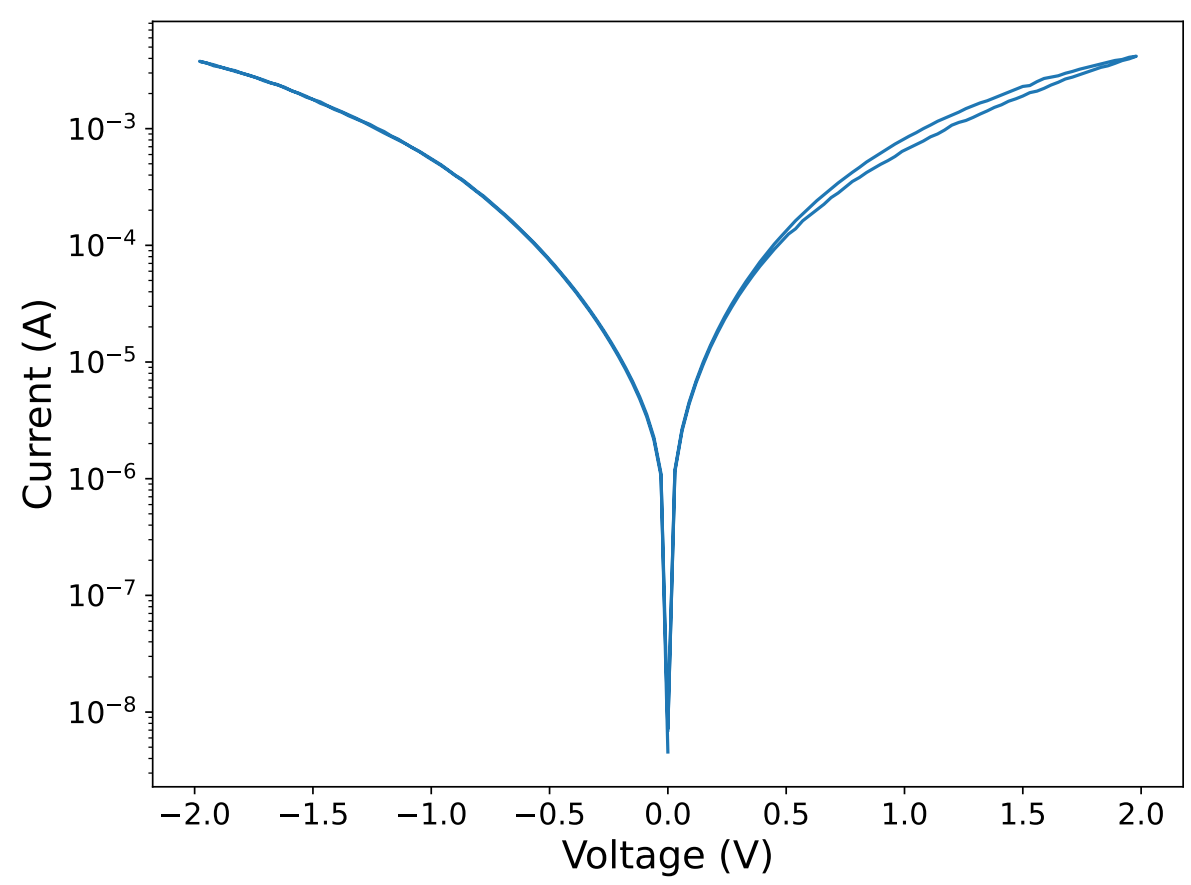

Figure S8: **Characteristic I-V curves from a Pt/Ta/α-STO/TaO_x/Ta device.** The voltage sweep (0 V to 2 V to −2 V to 0 V) is applied on the top electrode of a $100 \times 100\,\mu m^2$ pad size device. The device shows leaky conduction.

S10 Conduction through the STO/TaO_x/Ta barrier

The high oxygen vacancy concentration in the naturally formed $\sim$ 3.5 nm thick TaO_x layer, that act as trap states, facilitate the so-called trap-assisted tunneling (TAT) mechanism through the barrier. TAT is considered to involve a two-step process: electrons tunneling from the injection side of the barrier into a localized trap state, and subsequently from this trap state to the opposite side of the barrier. For the assumed oxygen vacancy trap concentrations and trap depths reported in the TaO_x literature,[11–18] this mechanism is expected to be highly favorable over direct tunneling described by the Tsu-Esaki formalism (S13), as the effective tunneling distance is approximately half of that required for direct tunneling. Indeed the estimated current densities for TAT are orders of magnitude larger than those estimated for direct tunneling as shown in Figure S9.

Hereby the TAT current densities were calculated according to

$$\begin{aligned} J_{TAT} = J_{s\to m} - J_{m\to s} = \int_{E_{min}}^{E_{max}} dE \int_{x_{in}}^{x_{out}} dx\, eC_t D(E_C(x) - E) \cdot \Bigg[\frac{f_s(E)[1-f_m(E)]TC(E)}{f_s(E)P_1(E,x) + [1-f_m(E)]P_2(E,x)} \\ - \frac{f_m(E)[1-f_s(E)]TC(E)}{f_m(E)P_2(E,x_{out}-x) + [1-f_s(E)]P_1(E,x_{out}-x)} \Bigg] \end{aligned} \tag{1}$$

with the electron tunneling energy E bounded by the highest conduction band minimum $E_C(x)$ on both sides of the barrier E_{min} and the maximum barrier height (given by the maximum of the conduction band edge $E_C(x)$ inside the barrier) E_{max}, the DOS of the trap states (available for TAT) $D(E_C(x) - E)$ and the Fermi-Dirac distributions including the bias dependent Fermi level $f_{m,s}(E)$ for the metal and semiconductor (here α-STO) side of the barrier. Hereby, the conduction band minimum $E_C(x)$ is calculated by solving the Poisson equation in steady-state numerically for an arbitrary heterostructure with an arbitrary trap DOS. Additionally the tunneling probabilities into and out of a trap at position x are given by

$$P_1(E,x) = \exp\left(-\frac{2}{\hbar}\int_{x_{in}}^{x} dx \sqrt{2m_t^* \left[E_C(x) - E\right]}\right) \tag{2}$$

$$P_2(E,x) = \exp\left(-\frac{2}{\hbar}\int_{x}^{x_{out}} dx \sqrt{2m_t^* \left[E_C(x) - E\right]}\right) \tag{3}$$

and the corresponding full-barrier tunneling probability is given by

$$TC(E) = P_1(E,x)P_2(E,x) = \exp\left(-\frac{2}{\hbar}\int_{x_{in}}^{x_{out}} dx \sqrt{2m_t^* \left[E_C(x) - E\right]}\right) \tag{4}$$

with the effective tunneling mass m_t^* and the tunneling injection and ejection points x_{in} and x_{out}. The band- and trap-DOS related prefactors, including wavefunction-matching contributions, are captured in the slowly varying function

$$C_t = \left(\frac{m_{bar}^*}{m_{inj}^*}\right)^{5/2} \left(\frac{8E_1^{3/2}}{3\hbar\sqrt{E_C(x) - E - E_1}}\right) \tag{5}$$

where m_{bar}^* denotes the electronic effective mass within the barrier, m_{inj}^* the effective mass on the injection side of the barrier and $E_1 \approx 0.1 - 0.2$ eV the energy spacing of the bound states in the trap assumed to be given by the quantized levels in a Coulomb electrostatic trap well.[19–21]

This newly presented TAT framework represents a significant refinement compared to the classical descriptions,[19–24] as it incorporates arbitrary barrier shapes, thermal activation, and reverse current contributions.

Crucially for the presented work, this model not only demonstrates that TAT governs charge transport across the α-STO/TaO_x/Ta barrier, but also reveals that this barrier is - even at very low voltages ($U < 0.1$ V) - much leakier than the measured total device currents, as calculated in Figure S9 for the parameters summarized in Table S2. This indicates that the resistance associated with electron transport through this interface is negligible. Consequently, the TaO_x barrier is not current limiting and therefore cannot be the origin of the resistive switching.

To experimentally validate the theoretical prediction that the TaO_x layer is highly leaky, we fabricated a Ta/Ta_2O_5/Ta sample with 3.5 nm Ta_2O_5, see Methods. This system is expected to exhibit a barrier profile and band bending similar to those of the α-STO/Ta_2O_5/Ta interface, since the work function of Ta approximately matches both the electron affinity and the work function of STO, as listed in Table S2. The full oxide stoichiometry was chosen as stoichiometries in the range of $1.66 < x < 2.5$ cannot be attained in our system due to target poisoning at higher $p(O_2)$ and at lower sputtering power.[59] Therefore, we chose to have a full Ta oxide, as the $TaO_{1.66}$ has an extremely high oxygen vacancy concentration and this might lead to misinterpretation of the results.

Fitting the I-V characteristics of this device stack in Figure S10 with the TAT demonstrates the accuracy of the model, yielding an effective Ta_2O_5 thickness of $\approx$ 3.6 nm and an oxygen vacancy related trap concentration of $\approx 4 \times 10^{16}$ cm^{-3} near the center of the oxide layer. These values closely match the expected parameters from the deposition process, further confirming the validity of the TAT model.

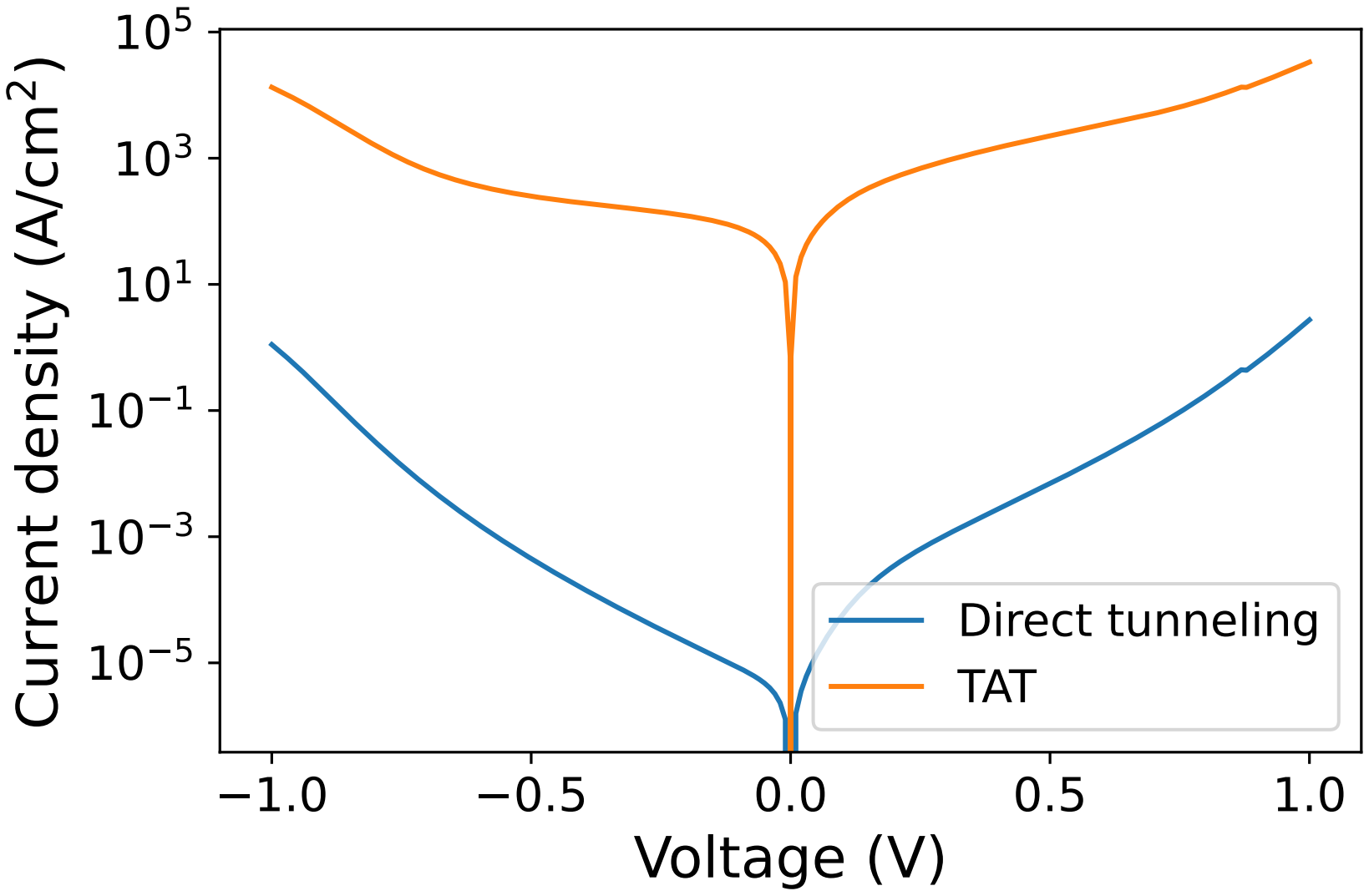

Figure S9: **TAT tunneling calculation.** Comparison of the calculated direct and trap-assisted current densities through the 3 nm TaO_x layer in a Pt/10 nm α-STO/3 nm TaO_x/Ta hetero structure, evaluated using the Tsu–Esaki direct-tunneling model (see Supplementary S13) and the newly developed TAT model. The simulations employ parameter values taken from Table S2, which are based on an extensive review of the relevant literature.

Physical parameter	Range of parameter based on literature	Value for simulation	Sources
Pt work function $e\phi_{m,\mathrm{TE}}$	$5.5\,\mathrm{eV} - 5.8\,\mathrm{eV}$	$5.6\,\mathrm{eV}$	[25]
Ta work function $e\phi_{m,\mathrm{BE}}$	$4.1\,\mathrm{eV} - 4.3\,\mathrm{eV}$	$4.2\,\mathrm{eV}$	[25]
STO dielectric constant $\varepsilon_{r,\mathrm{STO}}$	$20 - 150$	20^*	[10, 26–29]
STO effective mass m^*_{STO}	$1.8m_e - 16m_e$	$5m_e$	[29–33]
STO electron affinity $e\chi_{\mathrm{STO}}$	$4.0\,\mathrm{eV} - 4.3\,\mathrm{eV}$	$4.1\,\mathrm{eV}$	[34–36]
STO shallow trap depth $\phi_{t,\mathrm{STO}}$	$\phi_{t,\mathrm{STO}} \approx 0.15\,\mathrm{eV}$ with $\sigma \approx 0.1\,\mathrm{eV}$		[29, 37, 38]
STO deep trap depth $\phi_{t,\mathrm{STO}}$	$\phi_{t,\mathrm{STO}} \approx 1\,\mathrm{eV}$ with $\sigma \approx 0.1\,\mathrm{eV}$		[29, 37, 38]
STO trap concentration $N_{D,STO}$	$N_{D,STO} \approx 1 \times 10^{20}\,\mathrm{cm}^{-3}$ **		[10, 29, 39–41]
TaO_x dielectric constant $\varepsilon_{r,\mathrm{TaO}_x}$	$3 - 5$	4^*	[17, 42–45]
TaO_x effective mass $m^*_{\mathrm{TaO}_x}$	$0.5m_e - 2m_e$	$1m_e$	[46–50]
TaO_x electron affinity $e\chi_{\mathrm{TaO}_x}$	$3.1\,\mathrm{eV} - 3.3\,\mathrm{eV}$	$3.2\,\mathrm{eV}$	[44, 51, 52]
TaO_x shallow trap depth ϕ_{t,TaO_x}	$0.5\,\mathrm{eV} - 0.8\,\mathrm{eV}$ with $\sigma \approx 0.1\,\mathrm{eV}$	$0.65\,\mathrm{eV}$ with $\sigma = 0.1\,\mathrm{eV}$	[13, 14, 16, 17]
TaO_x deep trap depth 1. ϕ_{t,TaO_x}	$\phi_{t,\mathrm{TaO}_x} \approx 1.2\,\mathrm{eV}$ with $\sigma \approx 0.1\,\mathrm{eV}$		[15, 53]
TaO_x deep trap depth 2. ϕ_{t,TaO_x}	$\phi_{t,\mathrm{TaO}_x} \approx 1.5\,\mathrm{eV}$ with $\sigma \approx 0.1\,\mathrm{eV}$		[13, 18, 54]
TaO_x trap concentration N_{D,TaO_x}	$N_{D,\mathrm{TaO}_x} \approx 1 \times 10^{19}\,\mathrm{cm}^{-3}$		[54]
TaO_x Tunneling mass m^*_{t,TaO_x}	$0.5m_e$ - $2m_e$	$m^*_{\mathrm{TaO}_x}$	[55–58]
STO Tunneling mass $m^*_{t,\mathrm{STO}}$	$1m_e$ - $16m_e$	$1m_e$	[10, 29]
Temperature	$300\,\mathrm{K}$		

Table S2: Table with all the relevant parameters for electronic model simulations. In the table the range of the parameter and the baseline value for the simulations are given. The values marked by * are highly thickness dependent. The values marked by ** are just preliminary and can be changed drastically. Even during switching these values may change a lot. Because detailed analyses of α-STO are lacking, several α-STO–related parameters are estimated using the corresponding values for c-STO.

Although the I-V characteristics of the $Ta/Ta_2O_5/Ta$ device suggest significantly lower current densities than initially anticipated, these results nevertheless indicate that the TaO_x layer in α-STO-based devices is highly leaky. This behavior likely originates from the spontaneous formation of TaO_x during the deposition of α-STO, which promotes a substantially elevated concentration of oxygen vacancies and thereby enhances leakage conduction. Assuming an oxygen vacancy concentration of $1 \times 10^{19}\,\mathrm{cm}^{-3}$, corresponding to approximately 0.02% of oxygen sites being vacant, the estimated current density through the TaO_x layer exceeds both that of the Ta_2O_5/Ta device and the experimentally observed current densities in α-STO–based devices by several orders of magnitude.

Hence, these results provide direct experimental evidence for the highly conductive nature of the TaO_x layer. They further support the conclusion that, in the devices studied here, the TaO_x interface is not current-limiting and therefore cannot directly give rise to resistive switching.

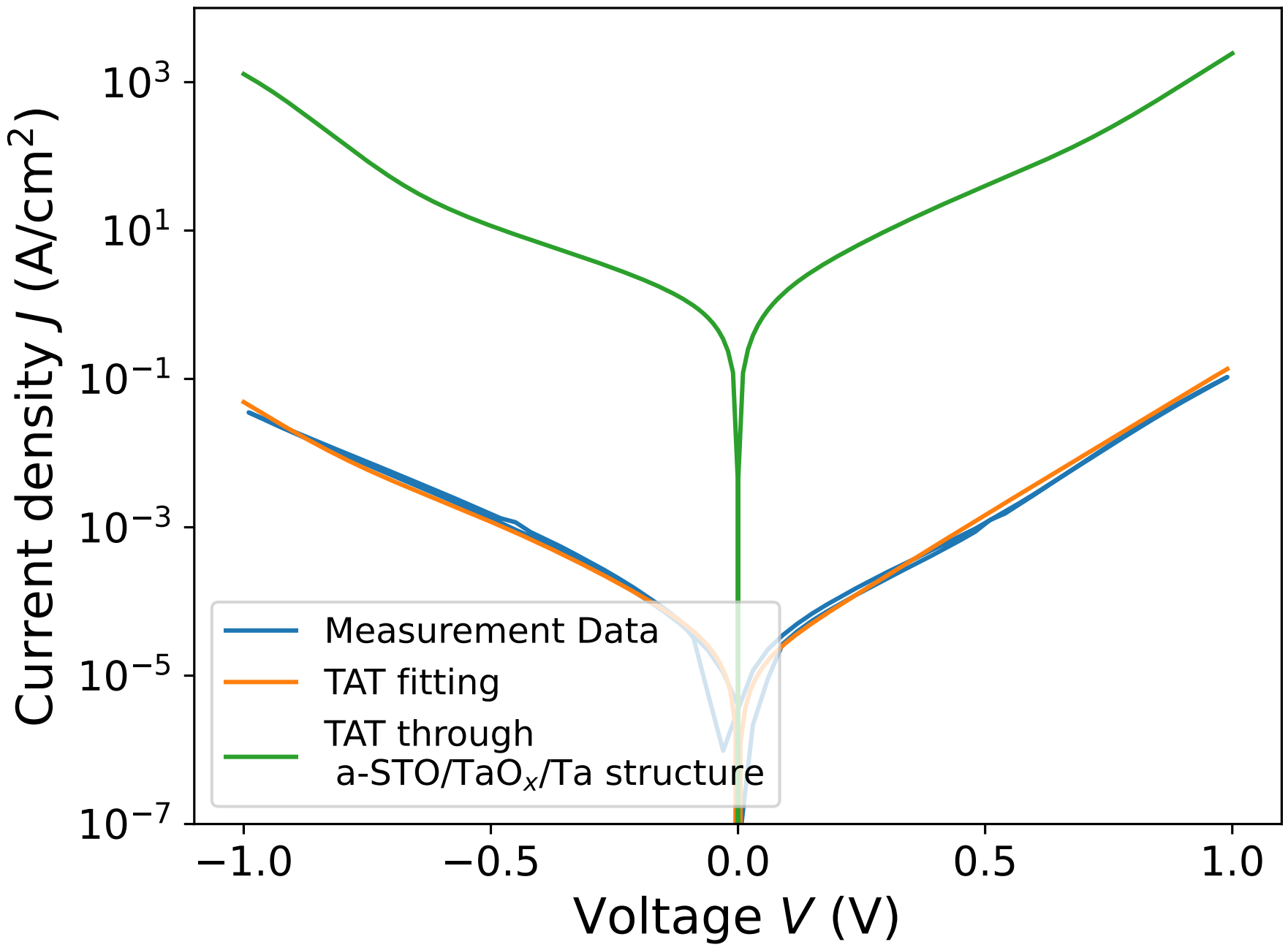

Figure S10: **Measured I-V characteristics of a Ta/(≈ 3 nm) Ta_2O_5/Ta device, together with corresponding fits using the TAT model.** The fits yield an effective oxide layer thickness of ≈ 3.6 nm and an oxygen-vacancy-related trap concentration of $\approx 4 \times 10^{16}\,\mathrm{cm^{-3}}$, in good agreement with the expected parameters based on the deposition conditions. All other parameters for the model were taken from Table S2. In addition, TAT current densities through an α-STO/(≈ 3.5 nm) TaO_x/Ta stack were calculated assuming a higher oxygen vacancy concentration of $\approx 1 \times 10^{19}\,\mathrm{cm^{-3}}$, using the parameters listed in Table S2. The TaO_x layer becomes much leakier with relatively low oxygen vacancy concentration of to demonstrate the leaky nature of $\approx 1 \times 10^{19}\,\mathrm{cm^{-3}}$.

S11 Nb:STO bottom electrode device

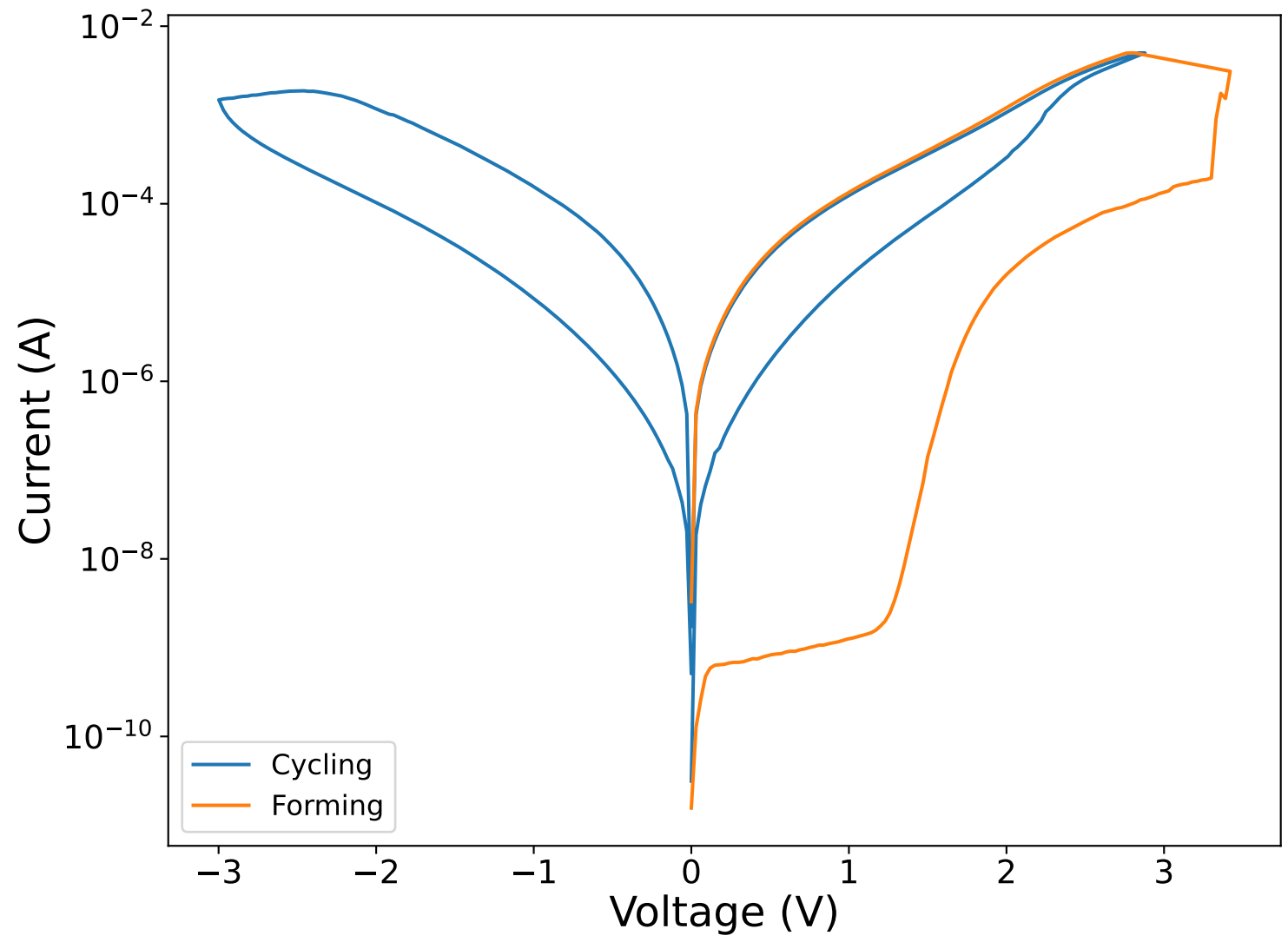

Figure S11: **Characteristic I-V curves from Pt/α-STO/Nb:STO devices with 5 nm α-STO.** Forming of the devices is achieved under around 3.5 V and 5 mA compliance current (orange curve). By applying a voltage sweep of (0 V to 3 V to −3 V to 0 V) with 5 mA compliance current, symmetric filamentary eightwise switching is observed (blue curve). The voltage is applied on the top electrode of a $100 \times 100\,\mu m^2$ pad size device.

S12 Temperature dependent pulsed I-V measurements

As outlined in S10, the Pt/α-STO Schottky barrier (in series with an ohmic resistor) must serve as the current-limiting element and therefore governs the overall current–voltage characteristics, given the high leakage of the TaO_x layer. To evaluate charge transport across the Pt/α-STO interface, we fit the measured $I - V$ behavior using the Tsu–Esaki tunneling model applied specifically to the Pt/α-STO Schottky barrier embedded within the Pt/α-STO/TaO_x/Ta stack. The steady-state $I - V$ characteristics were obtained using a pulsed-measurement protocol designed to isolate purely the electronic steady-state conduction, and thereby eliminating any contributions from trap based charging or ionic motion that would induce resistive switching. For comparatively small applied biases, $V \lesssim 2\,V$ (with the exact value depending on temperature), the steady-state High Resistive State (HRS) $I - V$ response could be measured using a single voltage pulse. The pulse duration ($t_{pulse} \approx 100\,ms$) was selected to be sufficiently long for the current to settle after initial electronic trap exchange-mediated transients. Because the current remained constant once these transients decayed, we can assume that such long pulses did not induce switching at these low voltages, or induced only negligible switching. At higher applied biases this approach no longer sufficed, as switching inevitably occurred during long single pulses. To extract the steady-state HRS $I - V$ characteristics under these conditions, we implemented a more sophisticated pulsing scheme consisting of a long, low-bias pre-pulse ($t_{prepulse} \approx 100\,ms, V \approx 1.8\,V$) immediately followed by a short high-bias read-out pulse ($t_{readout} \approx 1\,ms$). The pre-pulse allowed the dominant trap-related electron-exchange processes to relax almost completely before the read-out step, thereby ensuring rapid current stabilization at the higher bias and enabling measurement before significant switching occurred. Using this pulsing scheme for different temperatures we were able to obtain the steady state current-voltage relations presented in Figure S14.

To achieve a theoretical description consistent with the measured $I-V$ characteristics, we fitted the data using the Tsu–Esaki tunneling model applied to the Pt/α-STO Schottky barrier. As mentioned earlier, this approach is justified because electron transport across the α-STO/TaO_x/Ta stack is effectively resistance-free, and the overall currents are too small for any reasonable series resistance to influence the measurement.

A proper description of electron transport through the Pt/α-STO interface, however, requires more than consideration of an isolated Pt/α-STO Schottky barrier. Instead, the barrier must be treated as an integral part of the full device stack. Within this framework, we find that the continuity of the electric displacement field imposes substantial electric fields within the TaO_x layer, which in turn partially suppresses the field inside the α-STO as seen by comparing the band diagrams of the Pt/α-STO/TaO_x/Ta and Pt/α-STO/Nb:STO stack in Fig. 1d whereby the latter lacks the protective oxide layer. Notably, this effect also reduces the dependence of the Pt/α-STO Schottky barrier shape on the externally applied voltage, revealing a protective role of the TaO_x layer also as seen in S12. This protective behavior of the TaO_x layer influences not only electron transport — by reducing the voltage sensitivity of the current — but also the resistive switching dynamics. The resulting reduction (in the voltage dependence) of the local electric field suppresses the switching rate while enabling self-compliance behavior with larger switching amplitudes and without conductive filament formation. The switching behavior of the α-STO-based devices grown on Nb:STO is thereby shown in Supplementary S11, where the forming and sub-

sequent filamentary switching are presented. In particular, device stacks with the TaO_x protective layer enable more controlled modulation of the oxygen-vacancy concentration.

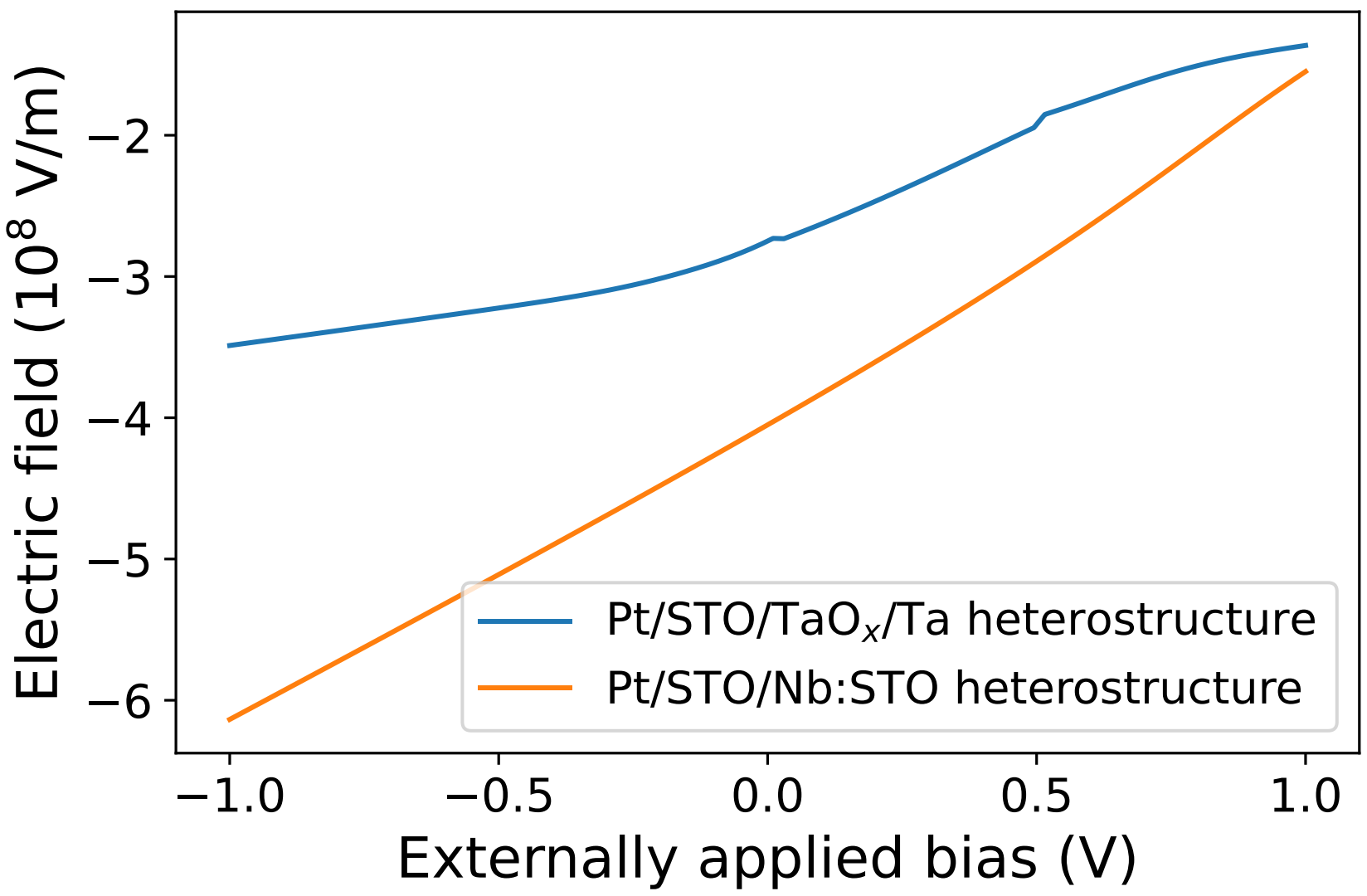

Figure S12: **Electric field calculations.** Comparison of the electric field strength inside the Pt/(5 nm)α-STO/(3 nm)TaO_x/Ta and Pt/(5 nm)α-STO/Nb:STO devices calculated at a position 2 nm away from the Pt/α-STO interface by solving the Poisson equation under an additional externally applied bias, using parameters listed in Table S2, with adjustments to match the fitting parameters reported in Table S3. The STO electron affinity was set to $\chi_{\mathrm{STO}} = 3.4\,\mathrm{eV}$, the relative permittivity to $\varepsilon_r = 10\varepsilon_0$, and the oxygen vacancy concentration in STO to $N_D \approx 5 \times 10^{19}\,\mathrm{cm}^3$.

However, even when accounting for the protective nature of the TaO_x layer, a fully quantitative description of the steady-state $I - V$ characteristics with physically reasonable parameters remained unattainable. To accurately model electron transport through the Pt/α-STO Schottky barrier — including its temperature dependence as shown in Figure S14 — it is essential to incorporate the spatial profile of the steady-state quasi-Fermi level. This sharp sigmoidal profile matching the metal Fermi level close to the interface and the bulk α-STO Fermi level $\gtrsim 2\,\mathrm{nm}$ further away from the interface (as shown in Figure S13) is thereby governed by tunneling-mediated electron exchange between localized trap states created by oxygen vacancies and the metal conduction band, together with thermally activated Shockley–Read–Hall exchange processes. Only upon including the quasi-Fermi level distribution determining the electron supply via the supply function (12) on the α-STO side of the barrier the tunneling current through the Pt/α-STO barrier can be fitted temperature dependently with realistic parameters (c.f. Table S3, as demonstrated in Figure S14. Hereby, for the purposes of fitting, we adopted a simplified functional form for the electron supply function, given by

$$N(E_x, V) = \begin{cases} 0 & \text{if } E_C^{-1}(E_x) \leq a \cdot \max E_C, \\ k_B T \ln \left[\frac{1+\exp\left(-\frac{E_x - eV}{k_B T}\right)}{1+\exp\left(-\frac{E_x - E_{Fm}}{k_B T}\right)} \right] & \text{if } E_C^{-1}(E_x) > a \cdot \max E_C \end{cases} \tag{6}$$

where $E_C^{-1}(E_x) = x \Leftrightarrow E_C(x) = E_x$ defines the inverse conduction-band profile within the barrier and the parameter $a \in [0, 1]$ specifies the energetic position in the potential barrier at which Shockley–Read–Hall exchange begins to dominate over direct tunneling exchange.

Overall, these realistic fitting results shown in Figure S14 demonstrate that the Pt/α-STO interface constitutes the current-limiting barrier, with electron transport accurately captured by Tsu–Esaki tunneling when the spatial profile of the quasi-Fermi level is included. It was further found that the TaO_x layer acts as a protective layer limiting the electric field inside the α-STO layer.

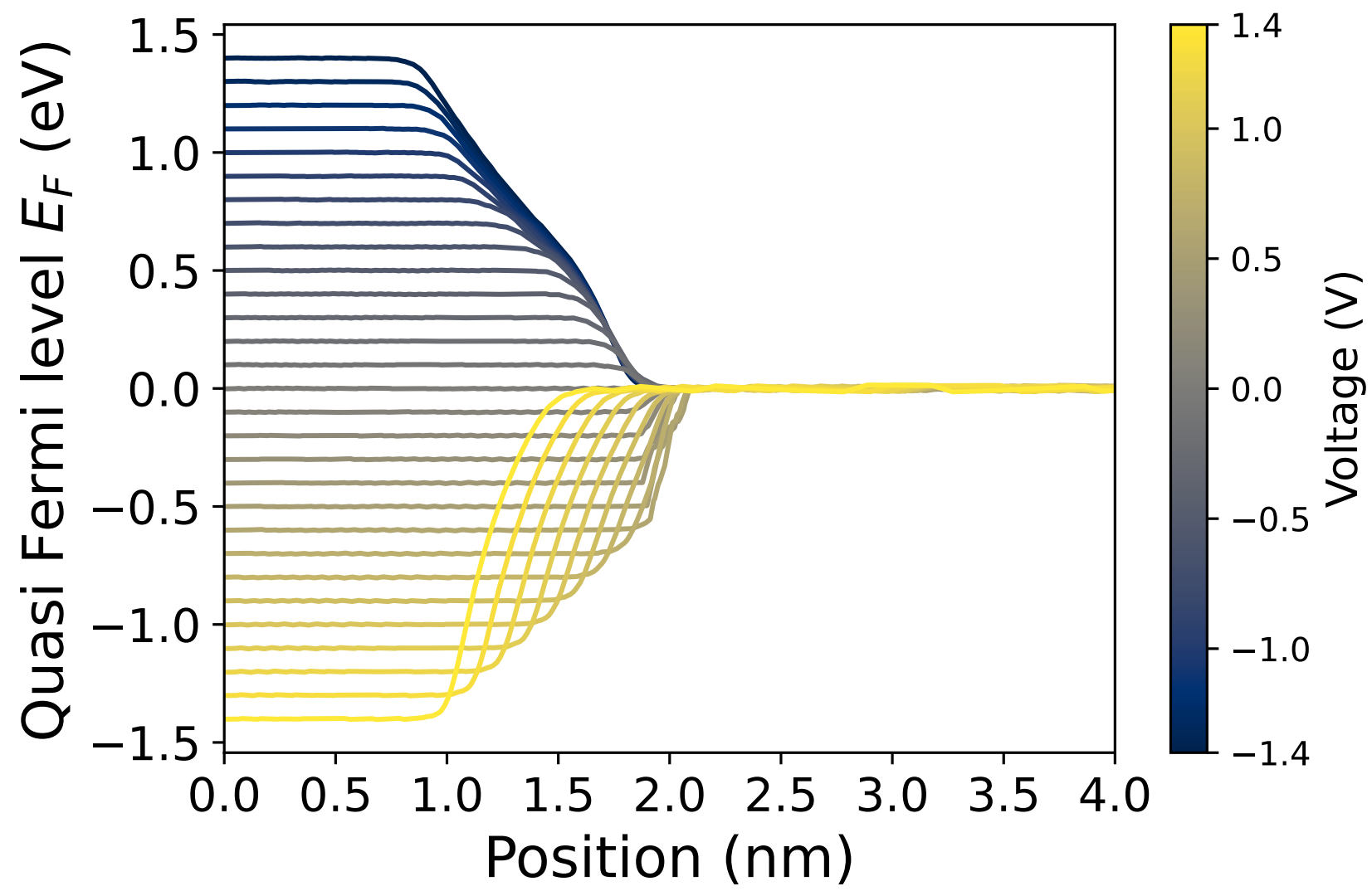

Figure S13: **Quasi-Fermi calculations.** Analysis of the quasi-Fermi level $E_F(x)$ at a Pt/α-STO interface for different applied biases between 1.4 V and −1.4 V. In total, a Pt/α-STO/TaO_x/Ta device with a 10 nm-thick α-STO layer was simulated, with the not-shown parts of the quasi-Fermi level given by $E_F(x \gtrsim 3\,\text{nm}) = 0\,\text{eV}$ for every applied bias. The simulation parameters were taken from Table S2, with the STO electron affinity set to $\chi_{\text{STO}} = 3.4\,\text{eV}$, the relative permittivity set to $\varepsilon_r = 10$, and the oxygen vacancy concentration in STO set to $N_D \approx 5 \times 10^{19}\,\text{cm}^{-3}$ to match the fitting parameters summarized in Table S3.

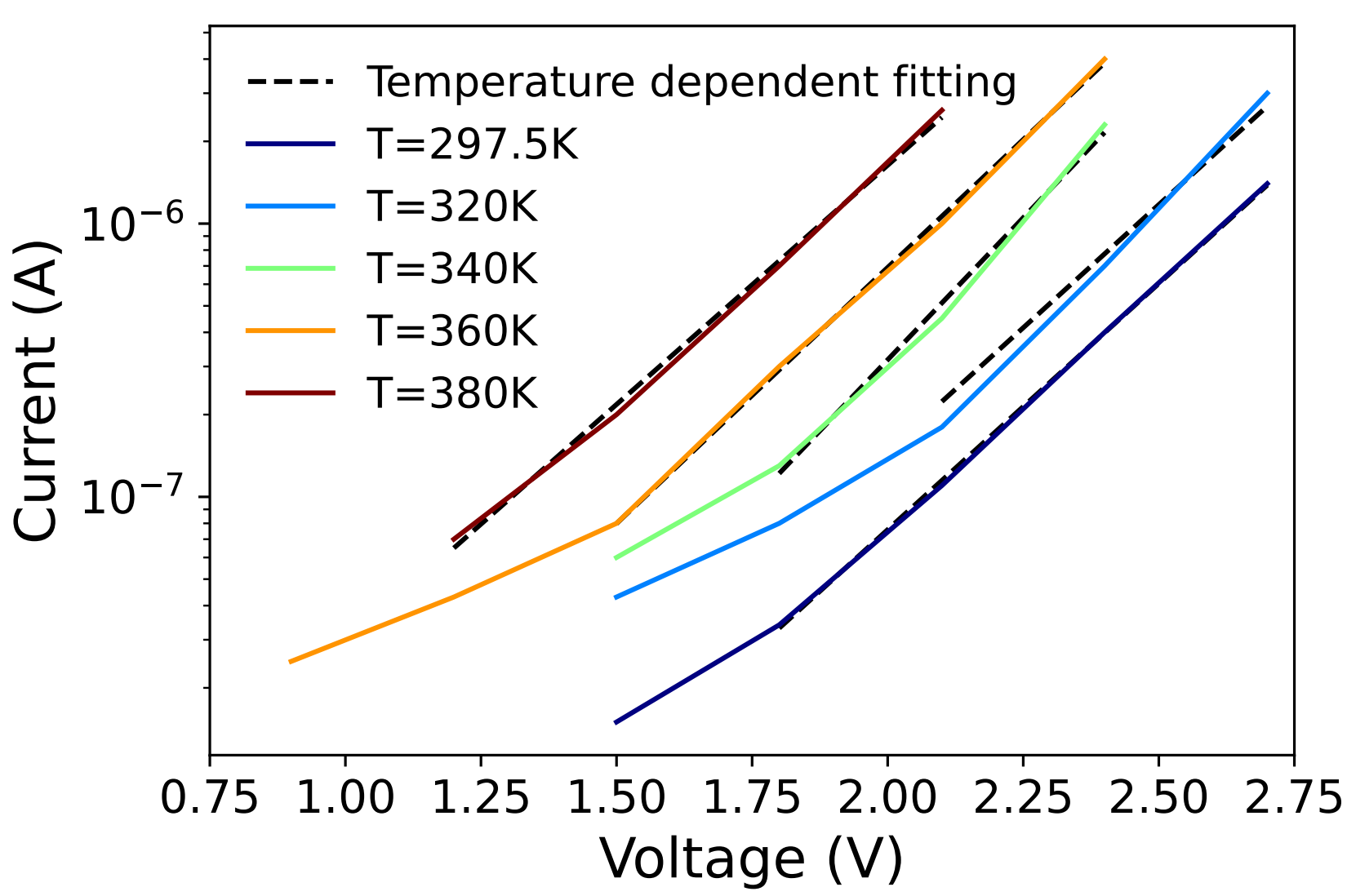

Figure S14: **Temperature-dependent I-V measurements.** Fits of the steady-state $I - V$ characteristics of a Pt/5 nm α-STO/TaO_x/Ta devices using the Tsu–Esaki tunneling model applied to the Pt/α-STO Schottky barrier, incorporating the spatial profile of the quasi-Fermi level. Despite the optimized pulsing protocol, the low-bias regime remains dominated by trap-induced charging of the Schottky barrier and therefore does not reflect steady-state electronic transport. The fitting parameters are summarized in Table S3, while all fixed parameters are taken from Table S2.

Parameter	Symbol	Value
Schottky barrier height	$e\phi_B$	$\approx 2.2\,\mathrm{eV}$
Conduction band offset	ΔE	$\gtrsim 0.22\,\mathrm{eV}$
Oxygen vacancy concentration	N_D	$\approx 5 \times 10^{19}\,\mathrm{cm}^{-3}$
Relative permittivity (STO)	$\varepsilon_{r,\mathrm{STO}}$	10
Cut-off energy parameter	a	$\gtrsim 36\,\%$

Table S3: **Fitting parameters used for the temperature-dependent transport analysis Pt/5 nm α-STO/TaO$_x$/Ta devices shown in Fig. S14**. The symbol $\gtrsim$ indicates that the parameter increases with temperature. All other parameters were assumed to remain constant and were taken from Table S2.

S13 Tsu-Esaki tunneling model

For a homogeneous charge distribution in equilibrium and under depletion approximation the Schottky barrier width L_D is given by:[60]

$$L_D(V) = \sqrt{\frac{2\epsilon_r\epsilon_0(\psi_{Bi} - V)}{e^2(N_D}} \tag{7}$$

Here, V is the applied voltage, ψ_{Bi} the built-in potential and N^+ and N^{++} are the concentration of singly and doubly charged oxygen vacancies, respectively.
The interface potential is given by:

$$\phi(x) = -\frac{qN_d}{2\epsilon_r\epsilon_0}L_{dp}^2(1 - \frac{x}{L_{dp}})^2 \tag{8}$$

and the resulting conduction band mininum $E_c(x)$:

$$E_c(x) = -\phi(x)e + E_{offset} \tag{9}$$

The tunnel current density through the Schottky barrier can be calculated using the Tsu-Esaki Formalism. It is described in detail by Funck et al.[29] The tunneling current density is given by the following expression:

$$j = \frac{A}{k_B^2}\int_{E_{min}}^{E_{max}} T(E_y) \cdot N(E_y, T)dE. \tag{10}$$

Here, $T(E_y)$ is the transmission function and $N(E_y, T)$ the supply function.
The transmission function $T(E_y)$ in WKB approximation is defined as:

$$T = \exp\left(-\frac{2}{\hbar}\int_{x1}^{L_{Ox}} \sqrt{2m_t}\sqrt{E_c(x) - E_c(x_1)}dx\right) \tag{11}$$

and the supply function N(E$_y$,T) as:

$$N = k_BT\ln\left(\frac{1 + \exp(-\frac{E_c - E_{f,n}}{k_BT})}{1 + \exp(-\frac{E_c - E_{f,Pt}}{k_BT})}\right) \tag{12}$$

Equation 10 can be used to calculate the current-voltage behavior and temperature dependence of memristive devices ,[29] accurately.

S14 Cyclic voltammetry

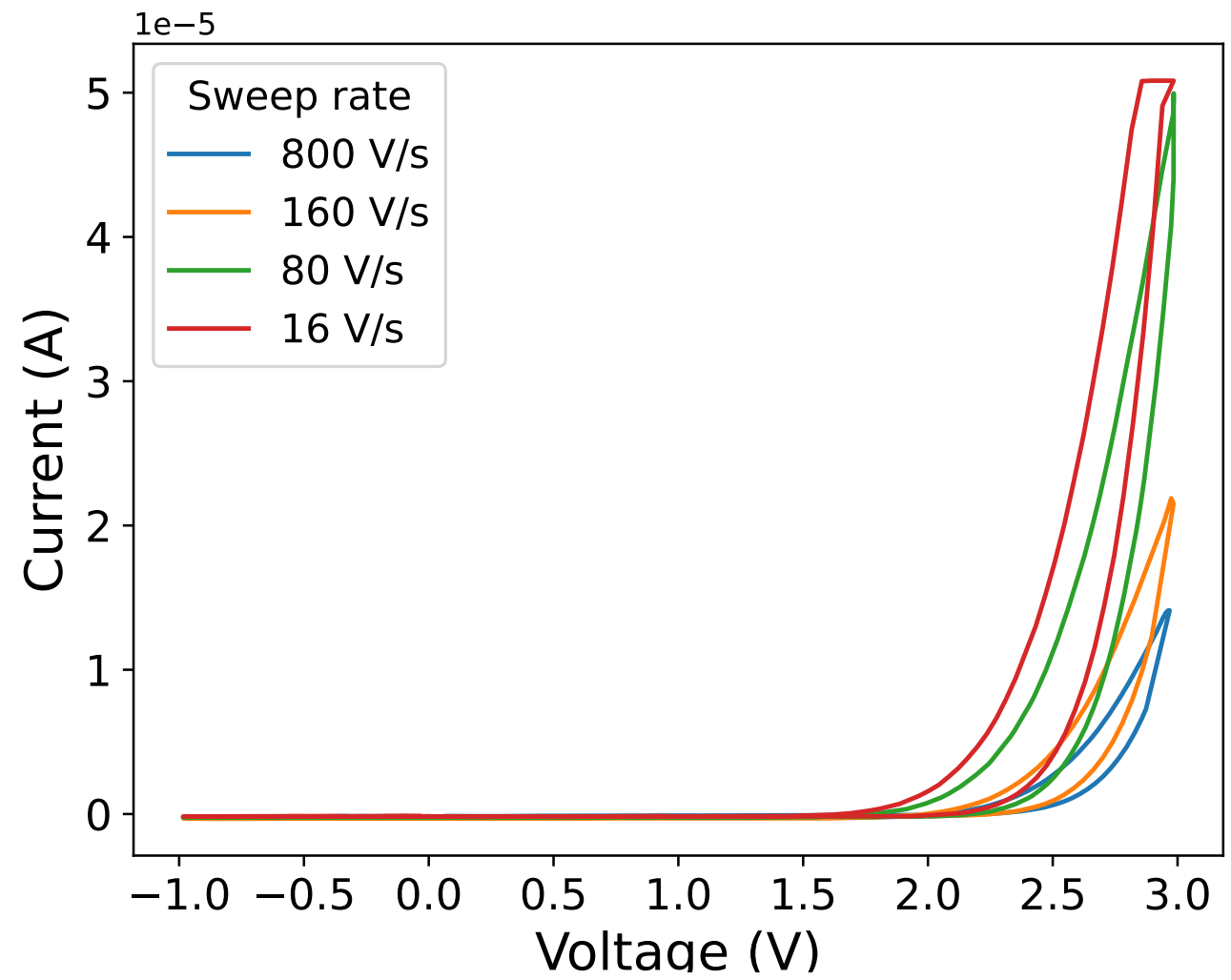

Figure S15: **Cyclic voltammetry I-V curves with different sweep rates**. Shown are sweep rates of 16, 80, 160 and 800 V/s from a 5 nm α-STO device of 100 $\times$ 100 μm^2 pad size. The voltage sweep (0 V to 3 V to −1 V to 0 V) is applied on the Pt top electrode. The window gets smaller with higher sweeping rate. The current range was limited to 5×10^{-5} A.

S15 Laplace Analysis of Time Constants

The inverse Laplace transformation of a current transient is a common method to disentangle multiple time constants originating from thermally activated processes.[61–63] Here, the relaxation is assumed to be described by the Laplace transform of a spectral density function $g(\tau)$:

$$I(t) = \int_0^\infty g(\tau) e^{-t/\tau}\, d\tau. \tag{13}$$

In this study, the inverse Laplace transformation was performed using the `Ridge` module from the Python library `scikit-learn`.[64] In this implementation, the following objective function is minimized:

$$f = \|I - Ag\|_2^2 + \alpha \|w\|_2^2, \tag{14}$$

where I represents the measured current transient, A is the transformation matrix, and w is the weight vector representing the time-constant spectrum. The parameter α denotes the regularization parameter controlling the smoothness of the solution.
The transformation matrix A is defined as

$$A_{ij} = \exp(-t_i/\tau_j). \tag{15}$$

For the time-constant spectra of the temperature-dependent decay measurements, a regularization parameter of $\alpha = 80$ was used.

S16 Arrhenius plot

The linear fit of the temperature induced peak shift was obtained using the python module *scipy.optimize.curvefit*. Here, the following equation was fitted to the data:

$$\ln(\frac{1}{\tau}) = -\ln(\tau_0) + \frac{E_a}{k_B T} \tag{16}$$

From this fit, $E_a = -0.127\,\mathrm{eV}$ and $\tau_0 = 0.002\,\mathrm{s}$ were obtained.

S17 Ionic hopping model of current decay

In the ionic hopping model the variation of oxygen vacancy concentration within the depletion region is mapped onto a change in current using the Tsu–Esaki formalism S13 to describe the current decay. Instead of treating the donor concentration of the Schottky contact as static as in Eq. 8, it is introduced here as a time-dependent variable $N_D(t)$. In the model it is calculated as the average of the three slabs:

$$N_D(t) = \frac{N_R(t) + N_S(t) + \alpha N_T(t)}{2+\alpha}, \tag{17}$$

where the transition layer T extends over more than one unit cell. The factor α accounts for the effective number of unit cells covered by this layer. As a result, the depletion width introduced in S13 evolves dynamically during reoxidation:

$$L_{dp}(t) = \sqrt{\frac{2\epsilon_0\epsilon_r\left(\psi_{bi} - \phi_{\text{applied}}\right)}{eN_D(t)}}. \tag{18}$$

The transient change in $L_{dp}(t)$ modulates the interface potential $\phi(t)$, thereby enhancing or suppressing the tunneling probability within the Tsu–Esaki framework.

To calculate the temporal evolution of the concentration of the individual slabs during the decay, three coupled ordinary differential equations (ODEs) can be derived for the oxygen vacancy concentration in each layer of the depletion zone. The differential equation for the recombination layer R is given by

$$\frac{dN_R}{dt} = \begin{cases} (R_{MR} + R_{SR}) \cdot (N_R - N_{\text{HRS}}), & N_R \geq N_{\text{HRS}}, \\ -(R_{RM} + R_{RS}) \cdot (N_{\text{HRS}} - N_R), & N_R < N_{\text{HRS}}, \end{cases} \tag{19}$$

for the supply layer S by

$$\frac{dN_S}{dt} = \begin{cases} (R_{RS} + R_{BS}) \cdot (N_S - N_{\text{HRS}}), & N_S \geq N_{\text{HRS}}, \\ -(R_{SR} + R_{SB}) \cdot (N_{\text{HRS}} - N_S), & N_S < N_{\text{HRS}}, \end{cases} \tag{20}$$

and for the transition layer T by

$$\frac{dN_T}{dt} = \begin{cases} (R_{ST} + R_{BT}) \cdot (N_T - N_{\text{HRS}}), & N_T \geq N_{\text{HRS}}, \\ -(R_{TS} + R_{TB}) \cdot (N_{\text{HRS}} - N_T), & N_T < N_{\text{HRS}}. \end{cases} \tag{21}$$

The parameter R_{ij} denotes the hopping rate from the i-th to j-th slab. The HRS oxygen vacancy concentration N_{HRS} at the readout voltage V_{read} is obtained by matching the concentration to the final value of the experimentally measured decay current.

Further details on the ionic hopping model can be found in the paper by Hellwig et al. [10] The parameters used in the simulations of Fig. 3 are listed in Table S4.

**Parameter**	**Symbol**	**Value**
Schottky barrier height	E_B	2.0 eV
Dielectric constant	ϵ_r	10
Oxygen vacancy concentration (HRS)	$N_{D,\text{HRS}}$	$1 \times 10^{25}\,\text{m}^{-3}$
Saddle-point energy (metal interface)	E_M	0.68 eV
Saddle-point energy (R region)	E_R	0.76 eV
Saddle-point energy (S region)	E_S	0.78 eV
Saddle-point energy (bulk region)	E_T	0.90 eV
Attempt frequency	ν_0	$1 \times 10^{13}\,\text{s}^{-1}$
Lattice parameter	a	0.39 nm

Table S4: **Ionic hopping model parameters.** The model describes oxygen vacancy exchange between three coupled regions (R, S, and T) within the depletion region of the Pt/α-STO Schottky contact. Migration barriers define the thermally activated hopping rates between regions, while the vacancy concentrations determine the depletion width and the resulting tunneling current through the Schottky barrier.

S18 Read-out voltage dependent current decays

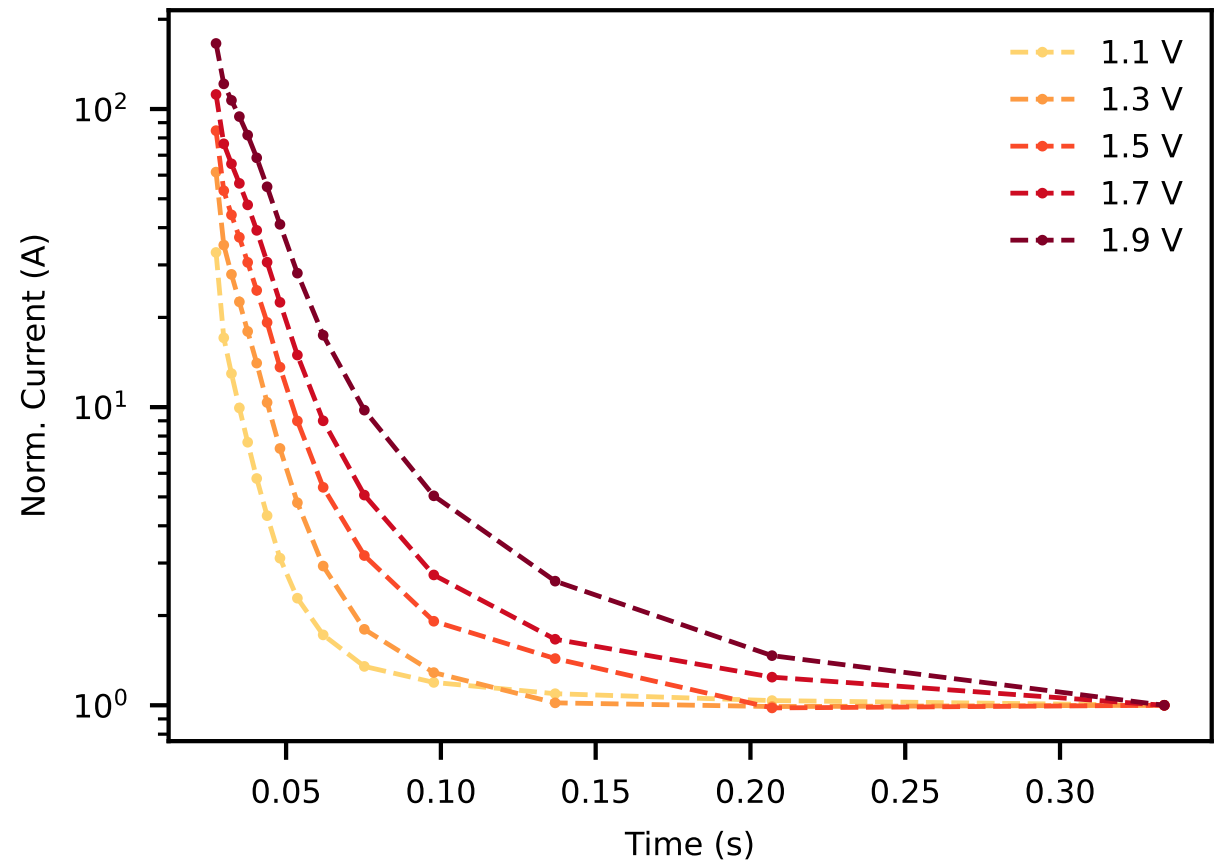

Figure S16: **Normalized READ sequence.** READ sequence dependent volatility for varying read-out voltage amplitude 0.9 V to 1.9 V with a fixed amount of read-out pulses. The normalized currents are presented here to make the difference in total decay time clear.

S19 Band diagram and Electric field simulations for 20 nm α-STO device

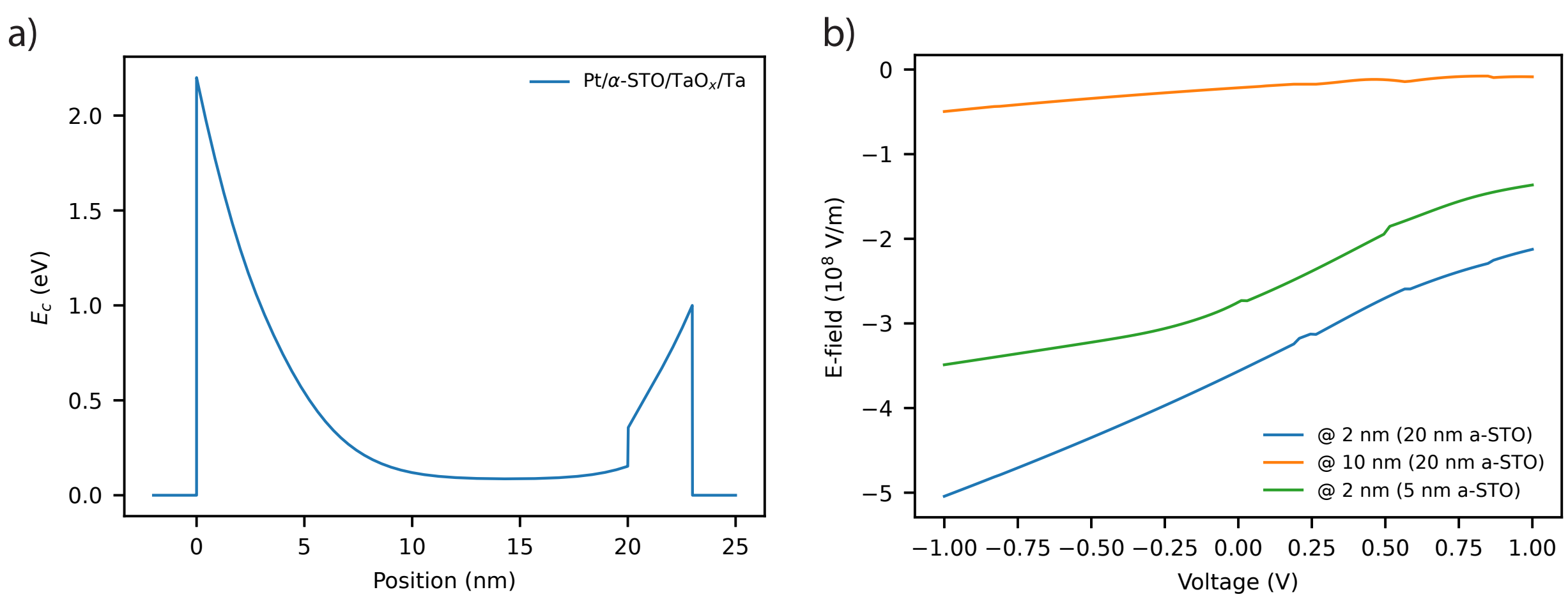

Figure S17: **Band diagram and electric field simulations.** a) Energy band diagram of the Pt/α-STO/TaO_x/Ta device stack with 20 nm α-STO calculated by solving the Poisson equation in equilibrium using parameters listed in Supplementary Table S2, with adjustments to match the fitting parameters reported in Supplementary Table S3. b) Comparison of the electric field strength inside the 5 nm and 20 nm α-STO devices, calculated at 2 nm and 10 nm away from the Pt/α-STO interface by solving the Poisson equation under an additional externally applied bias, using parameters listed in Supplementary Table S2, with adjustments to match the fitting parameters reported in Supplementary Table S3. The STO electron affinity was set to $\chi_{\text{STO}} = 3.4\,\text{eV}$, the relative permittivity to $\varepsilon_r = 10\varepsilon_0$, and the oxygen vacancy concentration in STO to $N_D \approx 5 \times 10^{19}\,\text{cm}^3$.

S20 Top electrode engineering

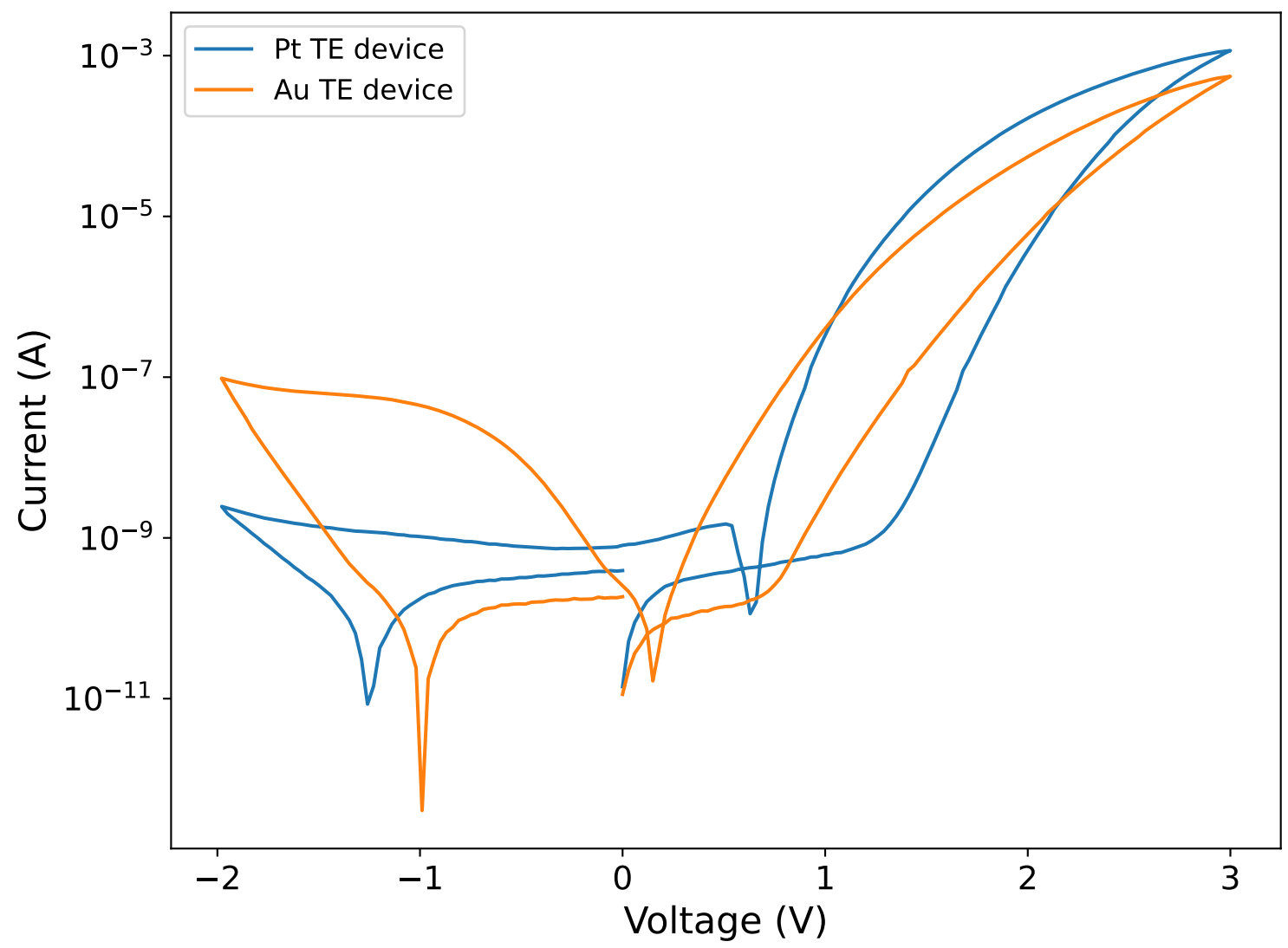

Figure S18: **Top electrode engineering.** Characteristic I-V curves from Au and Pt top electrode devices. The voltage sweep (0 V to 3 V to −2 V to 0 V) is applied on the top electrode of $100 \times 100\,\mu m^2$ pad size devices.

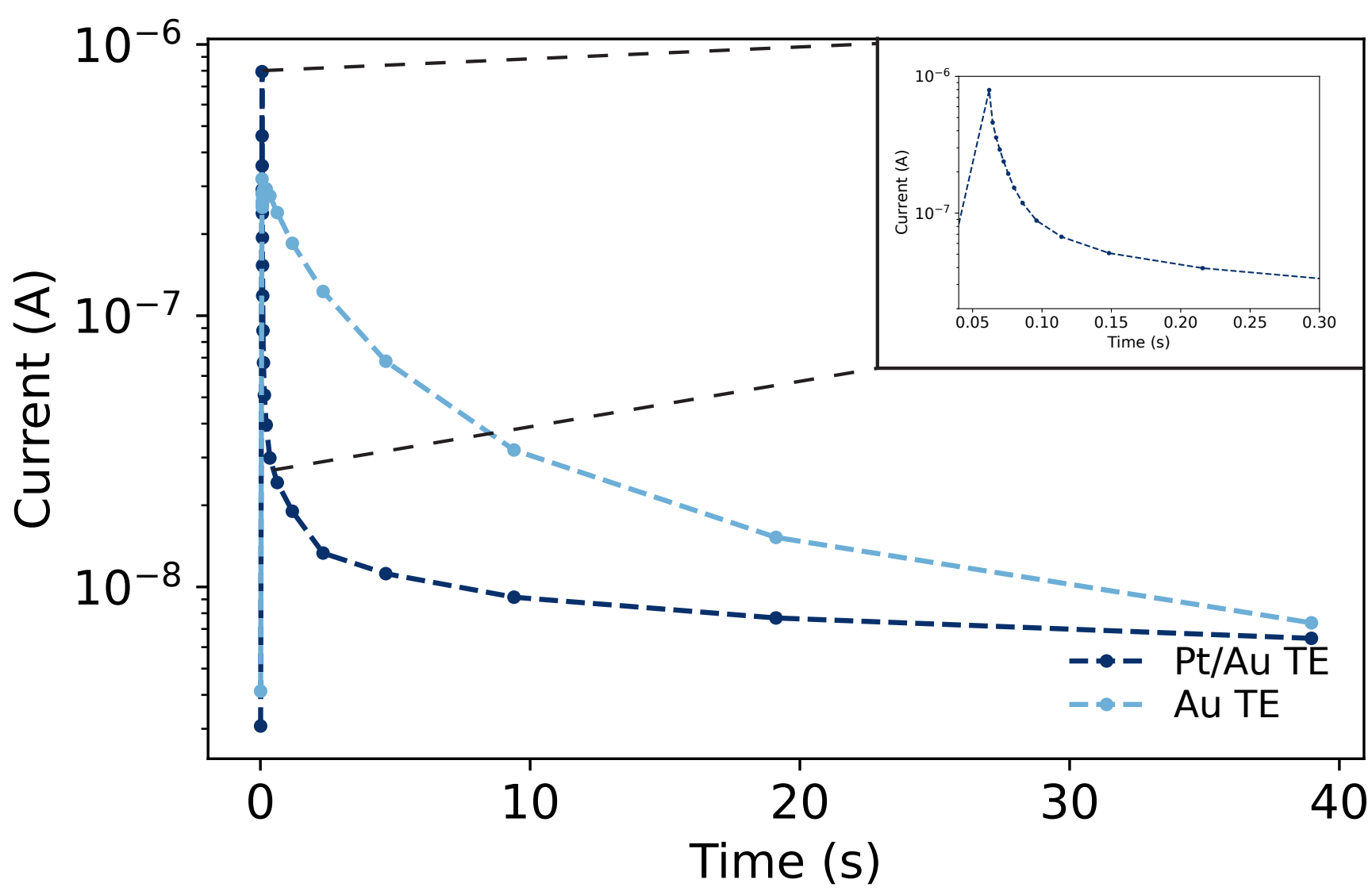

Figure S19: **Decay from Au/Pt top electrode device.** Current decays from 5 nm α-STO devices with i) 30 nm Au and ii) 1 nm Pt and 30 nm Au as a TE. The applied SET pulse consists of 3.5 V and 50 ms and the read-out voltage is 1.0 V in both cases. The Au TE device exhibits a decay of ∼ 40 s which is much longer than the usual Pt TE devices. Conversely, the decay from the Pt/Au TE device reflects a combined behavior from both of them. As it can be seen in the inset, it begins with rapid decay which lasts for ∼ 200 ms, like in the Pt TE devices, and then it enters a subsequent longer decay regime which lasts for ∼ 40 s like in the Au TE devices.

S21 C2C true decay variability

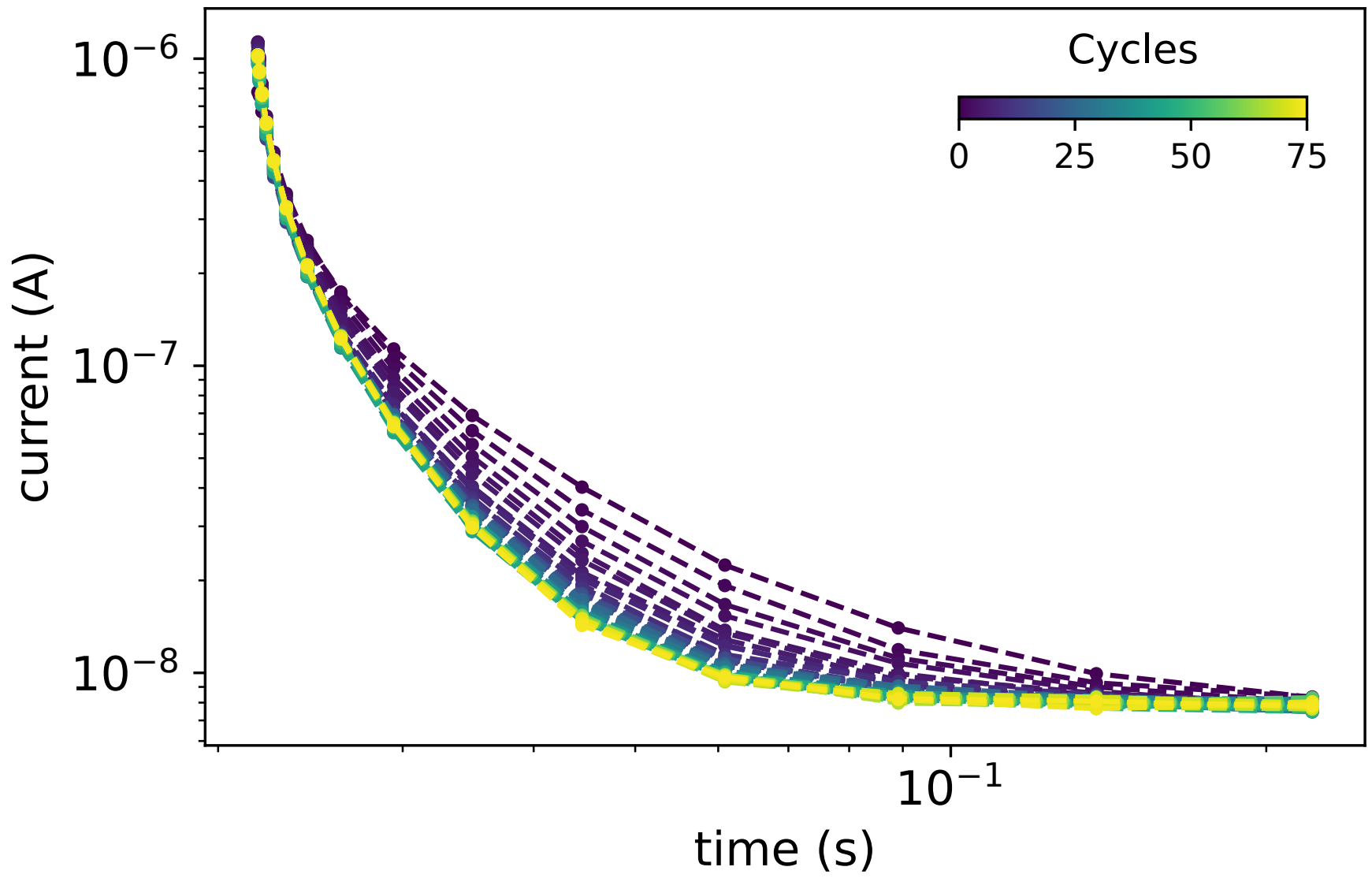

Figure S20: **Variability.** C2C true decay variability with precycling. During the precycling of 10 true decay cycles, the decay gradually drifts and becomes faster until it reaches a stable operation. The read-out voltage is set at 1.2 V.

S22 Variable decay demonstration

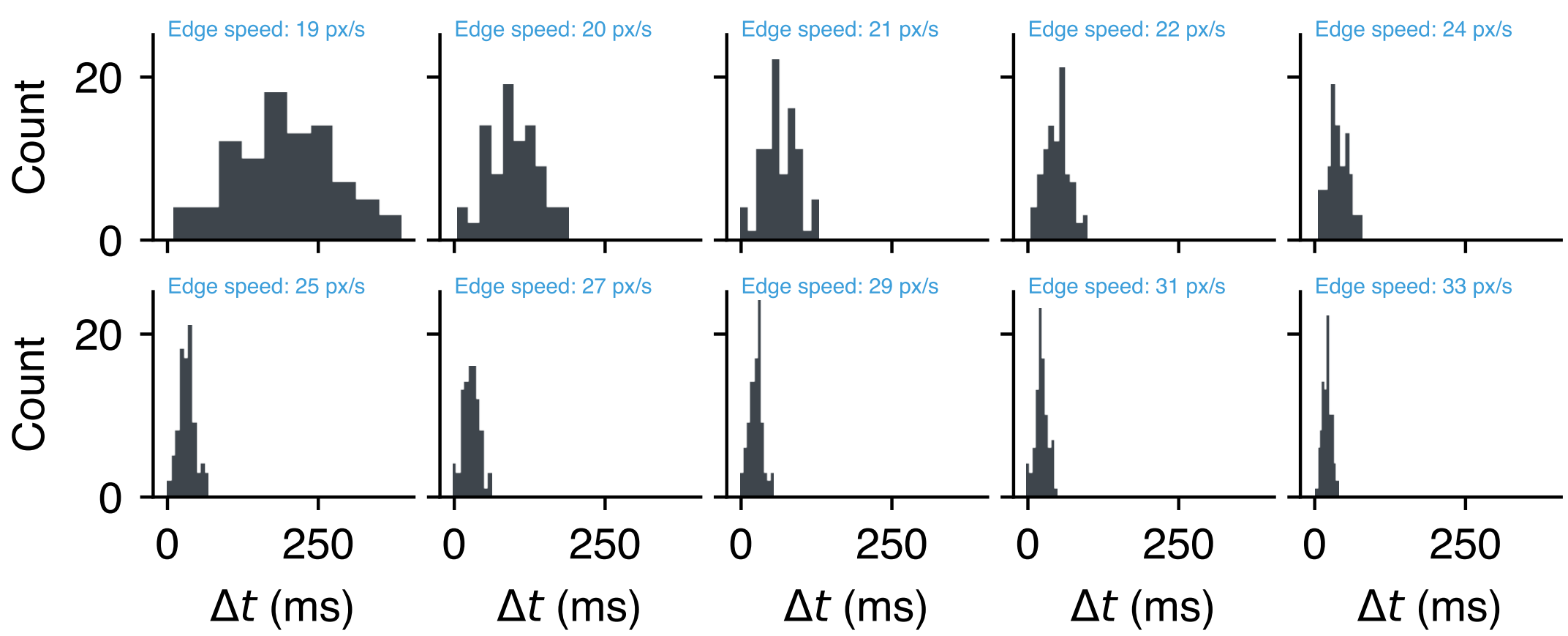

Figure S21: **Distribution of time differences (Δt) for different simulated moving bar speeds.**

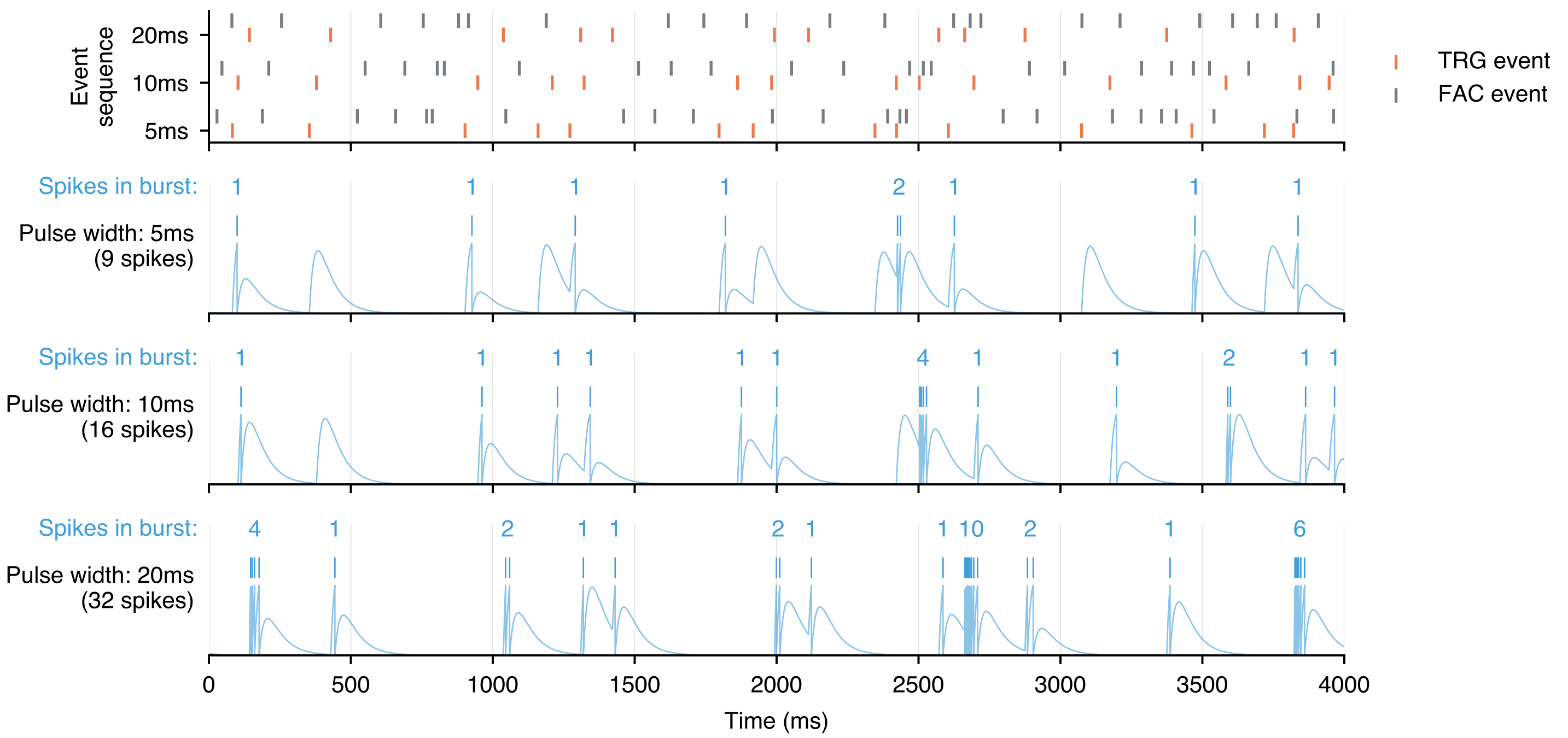

Figure S22: **Measurements of device time constant tunability.** Same data as Fig. 8d, presented without synchronizing the input sequences to account for longer pulse widths.

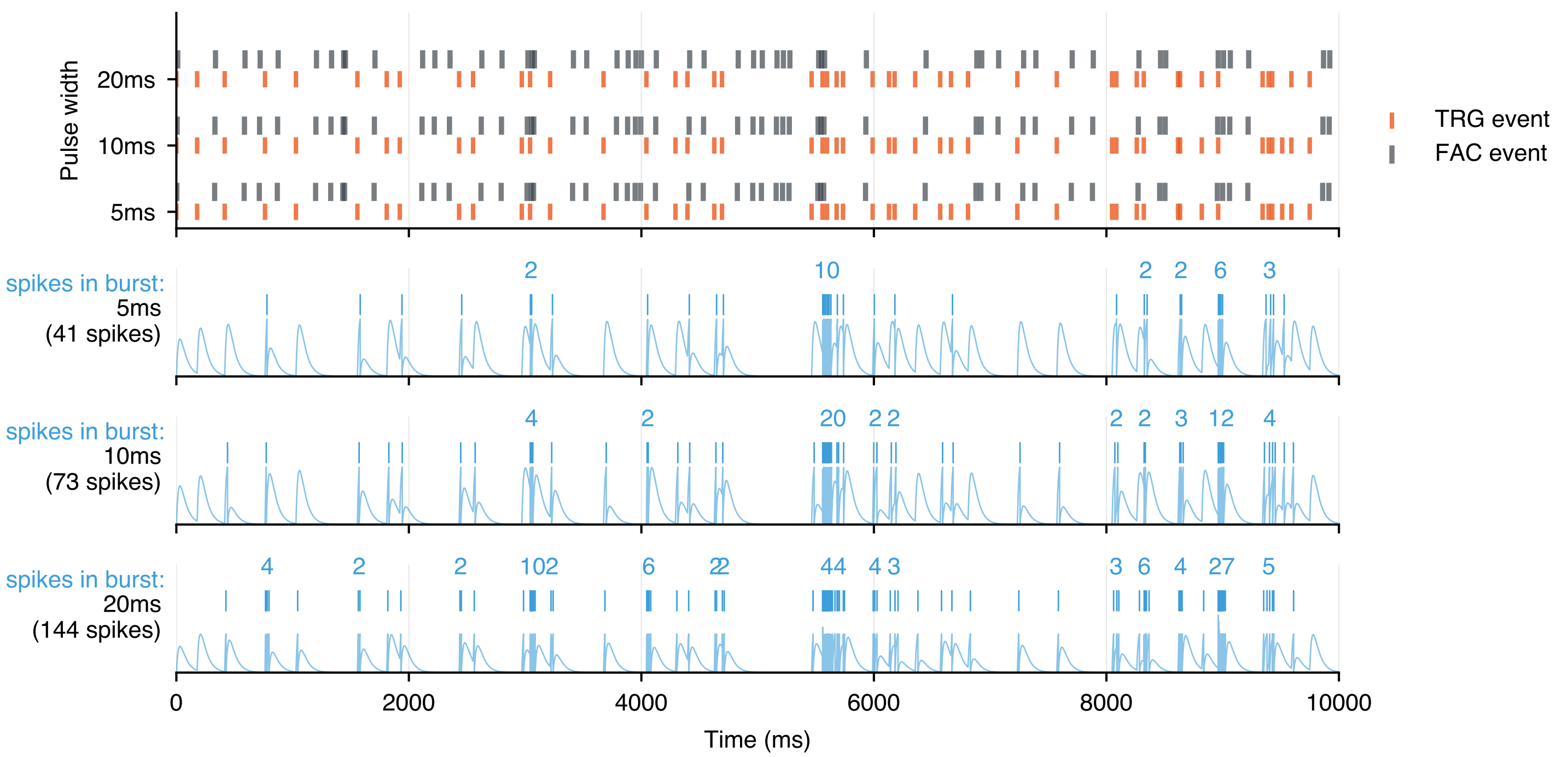

Figure S23: **Measurements of device time constant tunability.** Same data as Fig. 8d with full 10 second sequence.

Supplementary References

[1] C. Weilenmann et al., "Single neuromorphic memristor closely emulates multiple synaptic mechanisms for energy efficient neural networks," en, *Nat Commun*, vol. 15, no. 1, p. 6898, Aug. 2024, ISSN: 2041-1723. DOI: 10.1038/s41467-024-51093-3.

[2] J. Xiong et al., "Bienenstock, Cooper, and Munro Learning Rules Realized in Second-Order Memristors with Tunable Forgetting Rate," en, *Advanced Functional Materials*, vol. 29, no. 9, p. 1 807 316, 2019, _eprint: https://advanced.onlinelib ISSN: 1616-3028. DOI: 10.1002/adfm.201807316.

[3] X.-B. Yin et al., "Mimicking the brain functions of learning, forgetting and explicit/implicit memories with SrTiO 3 -based memristive devices," en, *Physical Chemistry Chemical Physics*, vol. 18, no. 46, pp. 31 796–31 802, 2016. DOI: 10.1039/C6CP06049H.

[4] F. Nie et al., "Ultrathin SrTiO3-based oxide memristor with both drift and diffusive dynamics as versatile synaptic emulators for neuromorphic computing," en, *Mater. Futures*, vol. 2, no. 3, p. 035 302, Jul. 2023, ISSN: 2752-5724. DOI: 10.1088/2752-5724/ace3dc.

[5] S. Kunwar et al., "An Interface-Type Memristive Device for Artificial Synapse and Neuromorphic Computing," en, *Advanced Intelligent Systems*, vol. 5, no. 8, p. 2 300 035, 2023, _eprint: https://advanced.onlinelibrary.wiley.com/doi/pdf/10.10 ISSN: 2640-4567. DOI: 10.1002/aisy.202300035.

[6] H. Nili et al., "Donor-induced performance tuning of amorphous srtio3 memristive nanodevices: Multistate resistive switching and mechanical tunability," *Advanced Functional Materials*, vol. 25, no. 21, pp. 3172–3182, Jun. 2015, ISSN: 1616-301X. DOI: 10.1002/adfm.201501019.

[7] T. Ahmed et al., "Inducing tunable switching behavior in a single memristor," *APPLIED MATERIALS TODAY*, vol. 11, pp. 280–290, Jun. 2018, ISSN: 2352-9407. DOI: 10.1016/j.apmt.2018.03.003.

[8] E. Mikheev, B. D. Hoskins, D. B. Strukov, and S. Stemmer, "Resistive switching and its suppression in pt/nb:srtio3 junctions," *Nature Communications*, vol. 5, Jun. 2014, ISSN: 2041-1723. DOI: 10.1038/ncomms4990.

[9] H. Tan, S. Majumdar, Q. Qin, J. Lahtinen, and S. van Dijken, "Mimicking neurotransmitter release and long-term plasticity by oxygen vacancy migration in a tunnel junction memristor," *Advanced Intelligent Systems*, vol. 1, no. 2, Jun. 2019. DOI: 10.1002/aisy.201900036.

[10] J. Hellwig et al., "Resolving the relaxation of volatile valence change memory," *Advanced Electronic Materials*, vol. 10, no. 12, p. 2 400 062, 2024. DOI: 10.1002/aelm.202400062.

[11] R. Fleming et al., "Defect dominated charge transport in amorphous ta2o5 thin films," *Journal of Applied Physics*, vol. 88, pp. 850–862, 2 2000. DOI: 10.1063/1.373747.

[12] J. Lee, W. Lu, and E. Kioupakis, "Electronic and optical properties of oxygen vacancies in amorphous ta2o5 from first principles," *Nanoscale*, vol. 9, pp. 1120–1127, 2016. DOI: 10.1039/C6NR07892C.

[13] W. Lau, L. Leong, T. Han, and N. P. Sandler, "Detection of oxygen vacancy defect states in capacitors with ultrathin ta2o5 films by zero-bias thermally stimulated current spectroscopy," *Applied physics letters*, vol. 83, pp. 2835–2837, 14 2003. DOI: 10.1063/1.1616990.

[14] V. Mikhaelashvili, Y. Betzer, I. Prudnikov, M. Orenstein, D. Ritter, and G. Eisenstein, "Electrical characteristics of metal-dielectric-metal and metal-dielectric-semiconductor structures based on electron beam evaporated y2o3, ta2o5 and al2o3 thin film," *Journal of applied physics*, vol. 84, pp. 6747–6752, 12 1998. DOI: 10.1063/1.369002.

[15] Y. Guo and J. Robertson, "Comparison of oxygen vacancy defects in crystalline and amorphous ta2o5," *Microelectronic Engineering*, vol. 147, pp. 254–259, 2015. DOI: 10.1016/j.mee.2015.04.065.

[16] H. Sawada and K. Kawakami, "Electronic structure of oxygen vacancy in ta2o5," *Journal of Applied Physics*, vol. 86, pp. 956–959, 2 1999. DOI: 10.1063/1.370831.

[17] N. Alimardani, J. McGlone, J. Wager, and J. Conley, "Conduction processes in metal–insulator–metal diodes with ta2o5 and nb2o5 insulators deposited by atomic layer deposition," *Journal of Vacuum Science & Technology A: Vacuum, Surfaces, and Films*, vol. 32, 01A122, 1 2014. DOI: 10.1116/1.4843555.

[18] J. Thomas III, "Defect photoconductivity of anodic ta2o5 films," *Applied Physics Letters*, vol. 22, pp. 406–408, 8 1973. DOI: 10.1063/1.1654692.

[19] S. Fleischer, P. Lai, and Y. Cheng, "Simplified closed-form trap-assisted tunneling model applied to nitrided oxide dielectric capacitors," *Journal of applied physics*, vol. 72, no. 12, pp. 5711–5715, 1992. DOI: 10.1063/1.351923.

[20] C. Svensson and I. Lundström, "Trap-assisted charge injection in mnos structures," *Journal of Applied Physics*, vol. 44, no. 10, pp. 4657–4663, 1973. DOI: 10.1063/1.1662016.

[21] X. Cheng, Y. Cheng, and B. Liu, "Nitridation-enhanced conductivity behavior and current transport mechanism in thin thermally nitrided sio2," *journal of applied physics*, vol. 63, no. 3, pp. 797–802, 1988. DOI: 10.1063/1.340072.

[22] W. J. Chang, M. P. Houng, and Y. H. Wang, "Simulation of stress-induced leakage current in silicon dioxides: A modified trap-assisted tunneling model considering gaussian-distributed traps and electron energy loss," *Journal of Applied Physics*, vol. 89, no. 11, pp. 6285–6293, 2001. DOI: 10.1063/1.1367399.

[23] W. J. Chang, M. P. Houng, and Y. H. Wang, "Electrical properties and modeling of ultrathin impurity-doped silicon dioxides," *Journal of Applied Physics*, vol. 90, no. 10, pp. 5171–5179, 2001. DOI: 10.1063/1.1389079.

[24] M. P. Houng, Y. H. Wang, and W. J. Chang, "Current transport mechanism in trapped oxides: A generalized trap-assisted tunneling model," *Journal of applied physics*, vol. 86, no. 3, pp. 1488–1491, 1999. DOI: 10.1063/1.370918.

[25] H. Kawano, "Effective work functions for ionic and electronic emissions from mono- and polycrystalline surfaces," *Progress in Surface Science*, pp. 1–165, 1-2 2008. DOI: 10.1016/j.progsurf.2007.11.001.

[26] N. Kim and J. Lee, "High-k amorphous srtio3 thin films for tactile displays," *Science of Advanced Materials*, vol. 7, no. 8, pp. 1530–1534, 2015. DOI: 10.1166/sam.2015.2370.

[27] M. N. Kamalasanan, N. D. Kumar, and S. Chandra, "Structural, optical, and dielectric properties of sol-gel derived srtio3 thin films," *Journal of Applied Physics*, vol. 74, pp. 679–686, 1 1993. DOI: 10.1063/1.355230.

[28] H. K. Chung et al., "Low temperature crystallization of atomic-layer-deposited srtio3 films with an extremely low equivalent oxide thickness of sub-0.4nm," *Applied Surface Science*, vol. 664, p. 160 243, 2024, ISSN: 0169-4332. DOI: 10.1016/j.apsusc.2024.160243.

[29] C. Funck et al., "Comprehensive model for the electronic transport in pt/srtio3 analog memristive devices," *Physical Review B*, vol. 102, p. 035 307, 3 2020. DOI: 10.1103/PhysRevB.102.035307.

[30] W. Gong, H. Yun, Y. B. Ning, J. E. Greedan, W. R. Datars, and C. V. Stager, "Oxygen-deficient srti$O_{3\text{-}x}$, x = 0.28, 0.17, and 0.08. crystal growth, crystal structure, magnetic, and transport properties," *Journal of Solid State Chemistry*, vol. 90, 2 1991. DOI: 10.1016/0022-4596(91)90149-C.

[31] H. P. R. Frederikse and W. R. Hosler, "Hall mobility in srtio3," *Physical Review*, vol. 161, pp. 822–827, 3 1967. DOI: 10.1103/PhysRev.161.822.

[32] M. Ahrens, R. Merkle, B. Rahmati, and J. Maier, "Effective masses of electrons in n-type srtio3 determined from low-temperature specific heat capacities," *Physica B: condensed Matter*, vol. 393, pp. 239–248, 2007. DOI: 10.1016/j.physb.2007.01.008.

[33] H. P. R. Frederikse, W. R. Thurber, and W. R. Hosler, "Electronic transport in strontium titanate," *Physical Review*, vol. 134, A442–A445, 2A 1964. DOI: 10.1103/PhysRev.134.A442.

[34] R. Schafranek, S. Li, F. Chen, W. Wu, and A. Klein, "$Pbtio_3$/$srtio_3$ interface: Energy band alignment and its relation to the limits of fermi level variation," *Physical Review B*, vol. 84, p. 045 317, 4 2011. DOI: 10.1103/PhysRevB.84.045317.

[35] Y. Hahn and D. Kim, "Structural and electrical properties of srtio 3 thin films prepared by plasma enhanced metalorganic chemical vapor deposition," *Journal of Vacuum Science & Technology A: Vacuum, Surfaces, and Films*, vol. 17, pp. 1982–1986, 4 1999. DOI: 10.1116/1.581714.

[36] G. Campet, M. Carrère, M. Puprichitkun, S. Wen, J. Salardennem, and J. Claverie, "N-type srtio3 thin films: Electronic processes and photoelectrochemical behavior," *Journal of Solid State Chemistry*, vol. 69, pp. 267–273, 2 1987. DOI: 10.1016/0022-4596(87)90083-1.

[37] C. Baeumer et al., "In-gap states and band-like transport in memristive devices," *Nano letters*, vol. 19, pp. 54–60, 2019. DOI: 10.1021/acs.nanolett.8b03023.

[38] J. Hellwig, C. Wittberg, D. Spithouris, and R. Dittmann, " two coexisting pathways to volatility in valence-change memory," unpublished research article, 2025.

[39] R. Dittmann, S. Menzel, and R. Waser, "Nanoionic memristive phenomena in metal oxides: The valence change mechanism," *Advances in Physics*, vol. 70, no. 2, pp. 155–349, 2021. DOI: 10.1080/00018732.2022.2084006.

[40] R. Waser, *Nanoelectronics and information technology: advanced electronic materials and novel devices*. John Wiley & Sons, 2012.

[41] D. Ielmini and R. Waser, *Resistive switching: from fundamentals of nanoionic redox processes to memristive device applications*. Wiley-VCH, 2016.

[42] N. Rausch and E. Burte, "Thin ta2o5 films prepared by low pressure metal organic cvd," *Microelectronics journal*, vol. 24, pp. 421–426, 4 1993. DOI: 10.1016/0026-2692(93)90048-J.

[43] S. Zaima, T. Furuta, Y. Yasuda, and M. Iida, "Preparation and properties of ta2o5 films by lpcvd for ulsi application," *Journal of the Electrochemical Society*, vol. 137, no. 4, p. 1297, 1990. DOI: 10.1149/1.2086651.

[44] J. Robertson, "Band offsets of wide-band-gap oxides and implications for future electronic devices," *Journal of Vacuum Science & Technology B: Microelectronics and Nanometer Structures*, vol. 18, pp. 1785–1791, May 2000. DOI: 10.1116/1.591472.

[45] I. Nemr Noureddine, N. Sedghi, I. Z. Mitrovic, and S. Hall, "Barrier tuning of atomic layer deposited ta2o5 and al2o3 in double dielectric diodes," *Journal of Vacuum Science & Technology B*, vol. 35, 1 2017. DOI: 10.1116/1.4974219.

[46] S. Zaima, T. Furuta, Y. Koide, Y. Yasuda, and M. Iida, "Conduction mechanism of leakage current in ta2 o 5 films on si prepared by lpcvd," *Journal of the Electrochemical Society*, vol. 137, no. 9, p. 2876, 1990. DOI: 10.1149/1.2087091.

[47] M. Houssa et al., "Trap-assisted tunneling in high permittivity gate dielectric stacks," *Journal of Applied Physics*, vol. 87, pp. 8615–8620, 12 2000. DOI: 10.1063/1.373587.

[48] P. L. Young, "Dc electrical conduction in thin ta2o5 films. ii. highly imperfect films," *Journal of applied physics*, vol. 47, pp. 242–247, 1 1976. DOI: /10.1063/1.322355.

[49] V. A. Shvets et al., "Electronic structure and charge transport properties of amorphous ta2o5 films," *Journal of Non-Crystalline Solids*, vol. 354, pp. 3025–3033, 26 2008. DOI: 10.1016/j.jnoncrysol.2007.12.013.

[50] M. Ivanov, T. Perevalov, A. Vladimir, V. Gritsenko, and V. Kaichev, "Electronic structure of δ-ta2o5 with oxygen vacancy: Ab initio calculations and comparison with experiment," *Journal of Applied Physics*, vol. 110, p. 024 115, 2011. DOI: 10.1063/1.3606416.

[51] R. Southwick III, A. Sup, A. Jain, and B. Knowlton, "An interactive simulation tool for complex multilayer dielectric devices," *Device and Materials Reliability, IEEE Transactions on*, vol. 11, pp. 236–243, 2011. DOI: 10.1109/TDMR.2011.2129593.

[52] S. Chakrabarti et al., "Evolution of resistive switching mechanism through h2o2 sensing by using taox - based material in w/al2o3/taox/tin structure," *Applied Surface Science*, vol. 433, pp. 51–59, 2017. DOI: 10.1016/j.apsusc.2017.10.072.

[53] A. Markosyan, R. Route, M. Fejer, D. Patel, and C. Menoni, "Study of spontaneous and induced absorption in amorphous ta2o5 and sio2 dielectric thin films," *Journal of Applied Physics*, vol. 113, 13 2013. DOI: 10.1063/1.4799415.

[54] R. Fleming et al., "Defect dominated charge transport in amorphous ta 2 o 5 thin films," *Journal of Applied Physics*, vol. 88, no. 2, pp. 850–862, 2000. DOI: 10.1063/1.373747.

[55] S. S. Sylvia, H.-H. Park, M. A. Khayer, K. Alam, G. Klimeck, and R. K. Lake, "Material selection for minimizing direct tunneling in nanowire transistors," *IEEE transactions on electron devices*, vol. 59, no. 8, pp. 2064–2069, 2012. DOI: 10.1109/TED.2012.2200688.

[56] S. M. Sze and K. K. Ng, *Physics of Semiconductor Devices*, 3rd ed. John Wiley & Sons, 2006.

[57] A. Gehring and S. Selberherr, "Tunneling models for semiconductor device simulation," *Handbook of theoretical and computational nanotechnology*, vol. 10, pp. 469–543, 2006.

[58] A. Messiah, *Quantum mechanics*. Courier Corporation, 2014.

[59] T. Heisig, "Spectromicroscopic investigation of local redox processes in resistive switching transition metal oxides," CitationKey: Heisig2021001 RefType: phdthesis LocalLink: file://X:/emrl/Pool/LitBib/Heisig/Heisig2021001.pdf, Ph.D. dissertation, RWTH Aachen University, 2021.

[60] M. Tyagi, *Physics of Schottky Barrier Junctions. In: Sharma, B.L. (eds) Metal-Semiconductor Schottky Barrier Junctions and Their Applications*. 1984, CitationKey: Tyagi1984001 Owner: JH RefType: book.

[61] D. K. Schroder, *Defects, in Semiconductor Material and Device Characterization*. 2006, CitationKey: Schroder2006001001 Owner: JH RefType: book.

[62] L. Schnorr, T. Heinzel, S. Scholz, A. Ludwig, and A. D. Wieck, "Laplace deep level transient spectroscopy on self-assembled quantum dots," *Journal of Applied Physics*, vol. 124, no. 10, p. 104 301, Sep. 2018, ISSN: 0021-8979. DOI: 10.1063/1.5028319. eprint: https://pubs.aip.org/aip/jap/article-pdf/doi/10.1063/1.5028319/13042235/104301_1_online.pdf.

[63] S. Reichert, J. Flemming, Q. An, Y. Vaynzof, J.-F. Pietschmann, and C. Deibel, "Ionic-Defect Distribution Revealed by Improved Evaluation of Deep-Level Transient Spectroscopy on Perovskite Solar Cells," *Physical Review Applied*, vol. 13, no. 3, p. 034 018, Mar. 2020, Publisher: American Physical Society. DOI: 10.1103/PhysRevApplied.13.034018.

[64] *Linear least squares with l2 regularization*, https://scikit-learn.org/stable/modules/generated/sklearn.linear_model.Ridge.html, Accessed: 2025-07-22.
